# BRD4 degradation blocks expression of MYC and multiple forms of stem cell resistance in Ph^+^ chronic myeloid leukemia

**DOI:** 10.1002/ajh.26650

**Published:** 2022-07-18

**Authors:** Barbara Peter, Gregor Eisenwort, Irina Sadovnik, Karin Bauer, Michael Willmann, Thomas Rülicke, Daniela Berger, Gabriele Stefanzl, Georg Greiner, Gregor Hoermann, Alexandra Keller, Dominik Wolf, Martin Čulen, Georg E. Winter, Thomas Hoffmann, Ana‐Iris Schiefer, Wolfgang R. Sperr, Johannes Zuber, Jiří Mayer, Peter Valent

**Affiliations:** ^1^ Ludwig Boltzmann Institute for Hematology and Oncology Medical University of Vienna Vienna Austria; ^2^ Department of Internal Medicine I, Division of Hematology & Hemostaseology Medical University of Vienna Vienna Austria; ^3^ Department for Companion Animals and Horses, University Clinic for Small Animals, Internal Medicine Small Animals University of Veterinary Medicine Vienna Vienna Austria; ^4^ Institute of Laboratory Animal Science University of Veterinary Medicine Vienna Vienna Austria; ^5^ Department of Laboratory Medicine Medical University of Vienna Vienna Austria; ^6^ MLL Munich Leukemia Laboratory Munich Germany; ^7^ Department of Hematology and Oncology Innsbruck Medical University Innsbruck Austria; ^8^ Department of Hematology, Oncology and Rheumatology, Center of Integrated Oncology Cologne Bonn University Hospital of Bonn Bonn Germany; ^9^ Department of Internal Medicine, Hematology and Oncology, Faculty of Medicine Masaryk University Brno Czech Republic; ^10^ Department of Internal Medicine, Hematology and Oncology University Hospital Brno Brno Czech Republic; ^11^ CeMM‐Research Center for Molecular Medicine of the Austrian Academy of Sciences Vienna Austria; ^12^ Research Institute of Molecular Pathology (IMP) Vienna BioCenter (VBC) Vienna Austria; ^13^ Department of Pathology Medical University of Vienna Vienna Austria; ^14^ Medical University of Vienna Vienna BioCenter (VBC) Vienna Austria

## Abstract

In most patients with chronic myeloid leukemia (CML) clonal cells can be kept under control by BCR::ABL1 tyrosine kinase inhibitors (TKI). However, overt resistance or intolerance against these TKI may occur. We identified the epigenetic reader BRD4 and its downstream‐effector MYC as growth regulators and therapeutic targets in CML cells. BRD4 and MYC were found to be expressed in primary CML cells, CD34^+^/CD38^−^ leukemic stem cells (LSC), and in the CML cell lines KU812, K562, KCL22, and KCL22^T315I^. The BRD4‐targeting drug JQ1 was found to suppress proliferation in KU812 cells and primary leukemic cells in the majority of patients with chronic phase CML. In the blast phase of CML, JQ1 was less effective. However, the BRD4 degrader dBET6 was found to block proliferation and/or survival of primary CML cells in all patients tested, including blast phase CML and CML cells exhibiting the T315I variant of BCR::ABL1. Moreover, dBET6 was found to block MYC expression and to synergize with BCR::ABL1 TKI in inhibiting the proliferation in the JQ1‐resistant cell line K562. Furthermore, BRD4 degradation was found to overcome osteoblast‐induced TKI resistance of CML LSC in a co‐culture system and to block interferon‐gamma‐induced upregulation of the checkpoint antigen PD‐L1 in LSC. Finally, dBET6 was found to suppress the *in vitro* survival of CML LSC and their engraftment in NSG mice. Together, targeting of BRD4 and MYC through BET degradation sensitizes CML cells against BCR::ABL1 TKI and is a potent approach to overcome multiple forms of drug resistance in CML LSC.

## 1 INTRODUCTION

Chronic myeloid leukemia (CML) is a stem cell‐derived hematopoietic neoplasm defined by the balanced chromosome translocation t(9;22).^1^, ^2^, ^3^ The resulting fusion gene, *BCR::ABL1*, encodes a 210 kDa oncoprotein (BCR::ABL1) that serves as a driver of disease initiation and evolution. BCR::ABL1 exhibits constitutive tyrosine kinase (TK) activity and triggers a series of downstream signaling pathways and molecules, leading to subsequent expression of survival factors and cell cycle regulators.^1^, ^2^, ^3^ These growth regulators and signaling cascades are supposed to act together to drive disease evolution and proliferation of neoplastic cells in patients with CML.

The BCR::ABL1 TK inhibitor (TKI) imatinib remains a gold‐standard in the treatment of CML.^4^, ^5^, ^6^, ^7^, ^8^ Indeed, most patients with chronic phase (CP) CML achieve a complete cytogenetic response (CCyR) and enter long‐term disease‐free survival when being treated continuously with imatinib.^4^, ^5^, ^6^, ^7^ However, in a substantial subset of patients, resistance or intolerance against imatinib occurs.^8^, ^9^, ^10^, ^11^, ^12^ In these cases, mutations in *BCR::ABL1* and/or in other relevant genes are known underlying causes of imatinib resistance in leukemic (stem) cells.^9^, ^10^, ^11^, ^12^ Imatinib‐resistant patients may respond to a second‐ or third‐generation BCR::ABL1 TKI, such as nilotinib, dasatinib, bosutinib, or ponatinib.^13^, ^14^, ^15^, ^16^, ^17^, ^18^, ^19^, ^20^ Several of these patients even enter continuous CCyR.^15^, ^16^ However, in other patients, resistance against second‐generation BCR::ABL1 TKI develops.^15^, ^16^ A particular problem is the T315I mutation in *BCR::ABL1*. This mutant mediates resistance against most BCR::ABL1 TKI, except ponatinib and asciminib.^18^, ^19^, ^20^, ^21^, ^22^ However, in advanced CML, neoplastic cells may even be resistant against ponatinib or asciminib, especially when these cells exhibit complex molecular aberration‐patterns or *BCR::ABL1* compound mutations.^23^, ^24^, ^25^ In addition, BCR::ABL1‐independent pathways and molecules or niche‐related factors may trigger growth and survival of leukemic stem cells (LSC) in CML and thereby contribute to drug resistance.^26^, ^27^, ^28^ Therefore, novel therapeutic targets and strategies are currently developed for the treatment of advanced CML to overcome resistance.^28^, ^29^, ^30^

During the past few years, several chromatin regulators have emerged as potential targets of human malignancies.^31^, ^32^, ^33^ One promising class of targets are bromodomain‐containing proteins.^32^, ^33^, ^34^ We have identified the “epigenetic reader” bromodomain‐containing protein 4 (BRD4) as a new therapeutic target in acute myeloid leukemia (AML).^34^, ^35^ In addition, we and others have shown that expression of MYC is regulated by BRD4 as well as by BRD4‐independent pathways in leukemic cells, and that re‐activation of MYC expression through the WNT‐pathway is associated with resistance against BRD4‐targeting drugs.^36^, ^37^

The present study aimed to explore whether CML cells express BRD4 and MYC and whether these antigens would serve as “druggable” targets in these patients. In addition, we asked whether BRD4/MYC‐inhibition is able to overcome multiple forms of LSC resistance in CML.

## 2 MATERIALS AND METHODS

### 2.1 Reagents

Reagents are described in Appendix S1. The BRD4‐targeting drugs dBET1 and dBET6 which induce degradation of BRD4, were either purchased or produced in‐house as described.^38^, ^39^

### 2.2 Cell lines and isolation of primary CML cells

Cell lines used in this study were KU812, K562, KCL22, KCL22^T315I^, Ba/F3 cells with BCR::ABL1^WT^, Ba/F3 cells containing BCR::ABL1^T315I^, and CAL‐72 cells. Primary CML cells were isolated from peripheral blood (PB) or bone marrow (BM) using Ficoll. Patients' characteristics are shown in Table S1. Magnetic cell sorting (MACS) was performed to enrich CD34^+^ cells and T cell‐depleted mononuclear cells (MNC). A detailed description is provided in Appendix S1.

### 2.3 Detection of BRD4 and MYC in CML cells

Quantitative real‐time polymerase chain reaction (qPCR) experiments were performed on CML cell lines and primary CML cells, including purified LSC essentially as reported.^35^, ^40^, ^41^ Primer sequences are shown in Table S2. Immunocytochemistry (ICC) was performed on cell lines and primary CML cells essentially as described.^35^, ^40^, ^41^ Immunohistochemistry (IHC) was performed on patient‐derived BM biopsy specimens following published protocols.^35^, ^40^, ^41^ Antibodies used in ICC and IHC experiments are shown in Table S3. Western blot experiments were performed essentially as reported.^41^ Technical details are provided in Appendix S1.

### 2.4 Knockdown of BRD4 and MYC in CML cells

KU812 and K562 cells were transduced with short hairpin RNAs (shRNAs) targeting BRD4 or MYC as described.^40^, ^41^ Details are provided in Appendix S1. shRNA sequences are shown in Table S4.

### 2.5 ^3^H‐thymidine uptake experiments and evaluation of apoptosis in CML cells

CML cells were incubated in medium with or without JQ1, dBET1, dBET6, or OTX‐015 for 48 hours (h). Thereafter, ^3^H‐thymidine uptake was measured and/or Annexin V staining experiments (for determining apoptosis) were performed essentially as described.^35^, ^40^, ^41^ A detailed description of methods is provided in Appendix S1.

### 2.6 Mouse xenotransplantation experiments

Primary CML cells (CD34^+^ cells from 2 CP patients and T cell‐depleted MNC from one patient with blast phase [BP] CML) were pre‐incubated in medium containing JQ1 (1 μM) or dBET6 (1 μM) at 37°C for 4 h, and were then injected into the tail veins of NOD.Cg‐*Prkdc* ^*scid*^ *Il2rg* ^*tm1Wjl*^ (NSG) mice (BP) or NSG_SCF_ mice (CP). After a maximum period of 10 weeks (BP) or 6 months (CP), engrafted cells were obtained from the BM of NSG/NSG_SCF_ mice and analyzed by flow cytometry. A detailed description is provided in Appendix S1.

### 2.7 Co‐culture experiments with osteoblast‐like cells and CML cells

Primary osteoblasts or the osteoblast‐like osteosarcoma cell line CAL‐72 were co‐cultured with primary CML cells (LSC) or with the cell lines K562 or KU812. Drug‐induced apoptosis was examined by flow cytometry. A detailed description of methods is provided in Appendix S1.

### 2.8 Evaluation of expression of resistance‐related checkpoint molecules on CML LSC

To examine whether BRD4‐targeting drugs counteract interferon‐gamma (IFN‐G)‐induced expression of PD‐L1 and other checkpoint‐molecules on CML cells, flow cytometry experiments were performed. A detailed description of methods is provided in Appendix S1.

### 2.9 Statistics

To determine significance, the two‐tailed paired or unpaired Student's *t*‐test was applied, unless otherwise stated. Results were considered significant when *p* < .05.

## 3 RESULTS

### 3.1 CML cells express BRD4 and MYC

**FIGURE 1:**
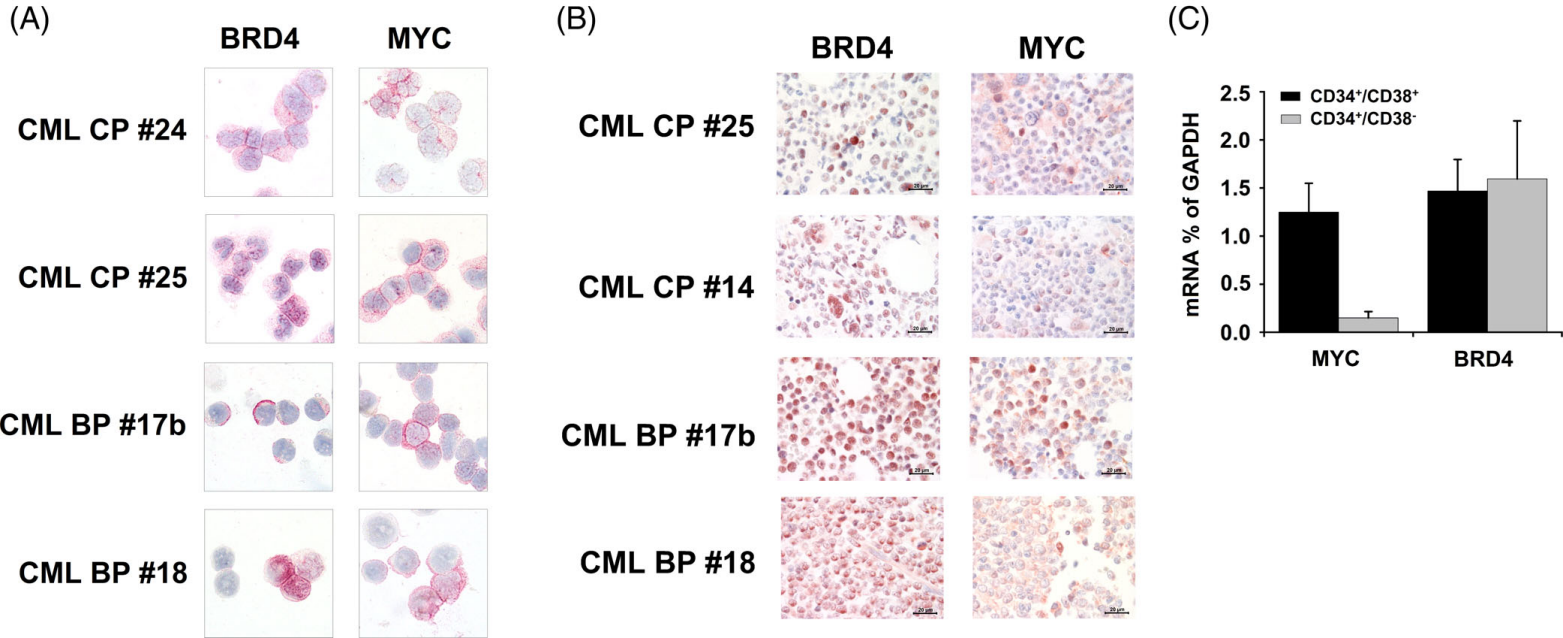
Expression of BRD4 and MYC in CML cells. (A) Immunocytochemical evaluation of BRD4 and MYC expression in primary CML MNC isolated from 2 patients with CML in chronic phase (CP) and 2 patients with CML blast phase (BP) was performed using a polyclonal antibody against BRD4 and a monoclonal antibody directed against MYC. Original magnification, ×100. (B) Immunohistochemical detection of BRD4 (left panels) and MYC (right panels) in CML in bone marrow biopsy sections in 2 patients with CML CP and 2 patients with CML BP. Original magnification, ×60. Slides were investigated using an Olympus DP21 camera connected to an Olympus BX50F4 microscope equipped with ×60/0.90 UPlanFL (IHC) or ×100/1.35 UPlanAPO (Oil Iris; ICC) objective lenses. Images were adjusted by Adobe Photoshop CS5. C: qPCR was performed using sorted CD34^+^/CD38^+^ and CD34^+^/CD38^−^ cells from patients with CP CML (*n* = 3). Results are expressed as BRD4 and MYC mRNA levels relative to (as percent of) GAPDH mRNA levels and represent the mean ± SD from three independent experiments. [Color figure can be viewed at wileyonlinelibrary.com]

As assessed by ICC and IHC, primary CML cells expressed substantial amounts of BRD4 and MYC in most patients examined without major differences in staining intensity when comparing CP and BP samples (Figure 1A,B). The CML cell lines tested (KU812, K562) were also found to express BRD4 and MYC (Figure S1A). BRD4 was detected in the nuclei as well as in the cytoplasm of CML cells (Figure 1A,B and Figure S1A). In qPCR experiments, BRD4 mRNA and MYC mRNA were detected in primary CML cells, KU812, K562 (Figure S1B,C), highly purified CD34^+^/CD38^−^ CML LSC, and CD34^+^/CD38^+^ CML progenitors (Figure 1C).

### 3.2 BCR::ABL1 TKI downregulate MYC expression in CML cells

To investigate whether MYC expression is BCR::ABL1‐dependent, KU812, K562, KCL22, and KCL22^T315I^ cells were incubated with BCR::ABL1 TKI. As determined by qPCR, imatinib, nilotinib, dasatinib, bosutinib, and ponatinib decreased the expression of MYC mRNA levels in KU812 cells and K562 cells (Figure S2A). Furthermore, all TKI were found to suppress expression of the MYC protein in these cells (Figure S2B). With the exception of imatinib, all the TKI tested also decreased MYC mRNA and protein expression levels in KCL22 cells, and, as expected, only ponatinib suppressed the expression of MYC mRNA and protein levels in KCL22^T315I^ cells (Figure S2A,B). Moreover, BCR::ABL1 TKI also slightly decreased BRD4 mRNA levels in KU812 cells and K562 cells, but such effects were not observed in KCL22 or KCL22^T315I^ cells (Figure S2C). To define signaling pathways downstream of BCR::ABL1, we applied drugs targeting MEK (refametinib = RDEA119, PD0325901, trametinib) or PI3‐kinase and mTOR (BEZ235). Whereas the MEK inhibitors suppressed the expression of MYC in KU812 and K562 cells, BEZ235 did not modulate MYC expression in these cells (Figure S2D).

### 3.3 BRD4 and MYC regulate growth of CML cells

In cell‐mixing experiments, shRNA against BRD4 and shRNA against MYC were found to counteract growth of KU812 and K562 cells (Figure S3A,B). The shRNA‐induced knockdown of BRD4 or of MYC resulted in a “growth‐disadvantage” compared to non‐transfected cells whereas no effects were seen with a control shRNA (Figure S3A,B). Knockdown of *BRD4* and *MYC* expression was confirmed by qPCR (Figure S3A,B).

### 3.4 Effects of BRD4‐targeting drugs on proliferation of CML cells

**FIGURE 2:**
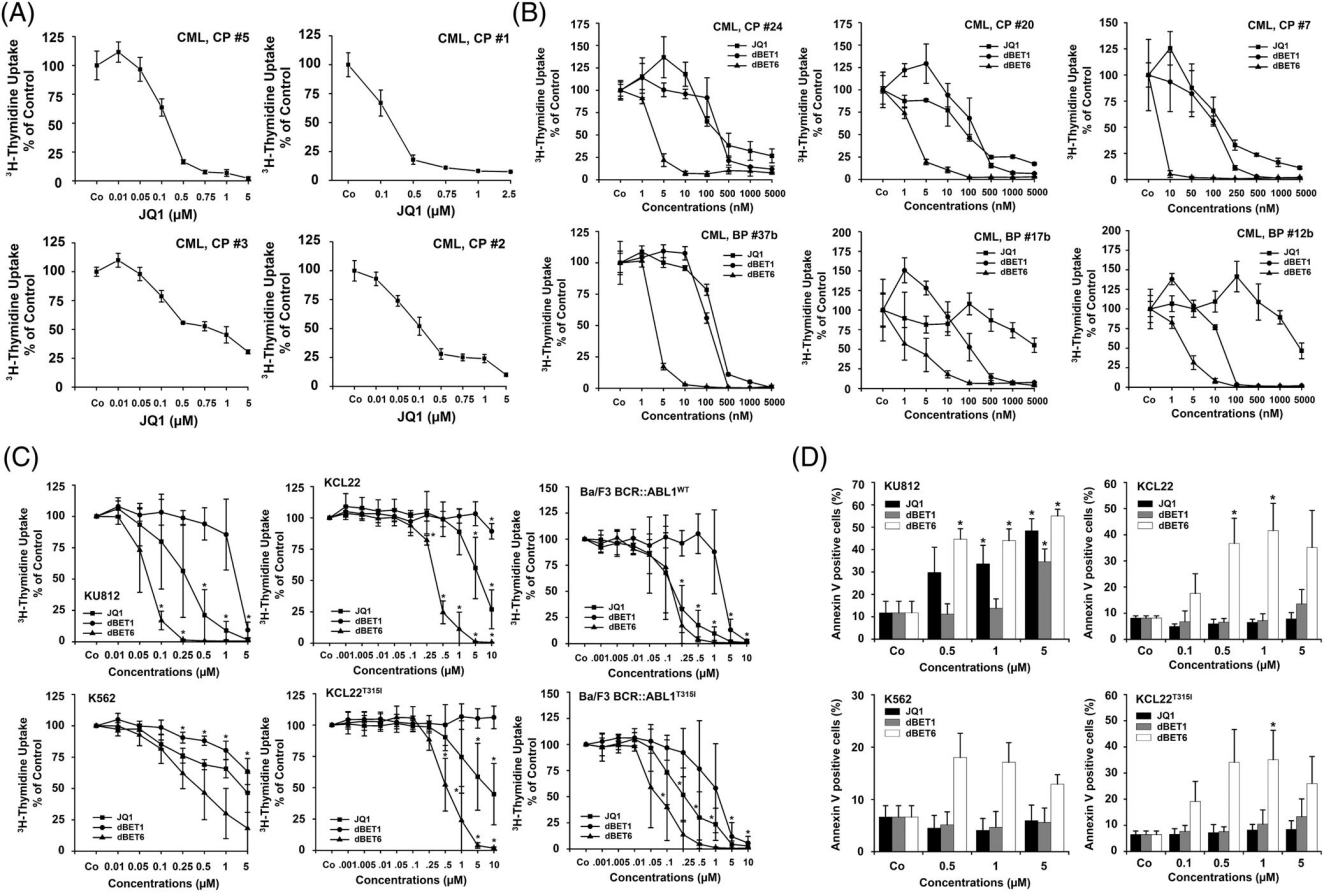
Effects of BET inhibitors/degraders on proliferation and survival of CML cells. (A) Primary chronic phase (CP) CML mononuclear cells (MNC; *n* = 4) were incubated in control medium (Co) or various concentrations of JQ1 at 37°C for 48 h. (B) MNC of 3 patients with CML CP (upper panel) and 3 with CML blast phase (BP, lower panel) were incubated in control medium (Co) or in various concentrations of JQ1, dBET1, or dBET6 for 48 h. (C) KU812, K562, KCL22, KCL22^T315I^, Ba/F3 BCR::ABL1^WT^ or Ba/F3 BCR::ABL1^T315I^ were incubated in control medium (Co) or various concentrations of JQ1, dBET1 or dBET6 for 48 h. Then, ^3^H‐thymidine uptake was measured. Results in “A and B” represent the mean ± SD from triplicates. Results in “C” are expressed as percent of control and represent the mean ± SD from at least four independent experiments. Asterisk: *p* < .05 compared to Co. D: KU812, K562, KCL22, and KCL22^T315I^ cells were incubated in control medium (Co) or various concentrations of JQ1, dBET1 and dBET6 for 48 h. Thereafter, Annexin V postitive cells (%) were analyzed by flow cytometry. Results represent the mean ± SD from three independent experiments. Asterisk: *p* < .05 compared to Co.

JQ1 was found to inhibit ^3^H‐thymidine uptake and thus proliferation of KU812 cells, with IC_50_ values of 0.25–0.75 μM (Figure 4A). By contrast, no substantial growth‐inhibitory effects of JQ1 were seen in K562 cells (IC_50_ > 5 μM) (Figure 4A). We were also able to show that JQ1 inhibits the growth of primary MNC obtained from patients with CML. However, IC_50_ values were found to vary among patients, ranging between 0.01 and 5 μM (Figure 2A,B, Table S5), and similar growth‐inhibitory effects on CML cells were seen with OTX‐015 (Figure S4B,C and Table S5). In most BP/AP CML samples tested, JQ1 did not exert major growth‐inhibitory effects (Figure 2B, Table S5). By contrast, in the majority of the CML CP samples tested (18/23) leukemic cells were JQ1‐sensitive (IC_50_: <750 nM) (Figure 2A,B, Table S5). Previously published data suggest that the BRD4 degraders dBET1 and dBET6 are more potent BRD4‐targeting drugs than JQ1.^38^, ^39^ We found that dBET1 and dBET6 inhibit proliferation of primary CML MNC, including BP cells. In all samples tested, lower IC_50_ values were obtained with dBET6 compared to JQ1 (Figure 2B, Table S5). We also found that dBET6 inhibits proliferation of K562 and KU812 cells (Figure 2C). However, dBET1 did not exert substantial growth‐inhibitory effects in these cells (Figure 2C). In addition, KCL22^T315I^ cells and the parental cell line KCL22 were found to be resistant against JQ1, whereas JQ1 suppressed the proliferation of Ba/F3 BCR::ABL1^WT^ and Ba/F3 BCR::ABL1^T315I^ cells (Figure 2C). dBET6 was found to inhibit proliferation in all these cell lines, whereas no substantial growth‐inhibitory effects were observed with dBET1 (Figure 2C).

### 3.5 Effects of BRD4‐targeting drugs on survival of CML cells

In Annexin V staining experiments, JQ1 induced apoptosis in KU812 cells in a dose‐dependent manner (Figure 2D). By contrast, JQ1 was not able to induce substantial apoptosis in K562, KCL22 or KCL22^T315I^ cells (Figure 2D). Similar results were obtained with OTX‐015 in KU812 cells and K562 cells (Figure S5). dBET1 also failed to induce apoptosis in all CML cell lines at pharmacologically meaningful concentrations (Figure 2D). By contrast, dBET6 induced apoptosis in KU812, K562, KCL22, and KCL22^T315I^ cells (Figure 2D).

### 3.6 HOXB4 and CCND2 mRNA are preferentially expressed in JQ1‐resistant cell lines and JQ1‐resistant primary CML cells

To explore mechanisms underlying JQ1 resistance, we compared mRNA expression levels of MYC and BRD4 as well as HOXB4 and CCND2 in JQ1‐sensitive KU812 cells and JQ1‐resistant K562, KCL22, and KCL22^T315I^ cells. No major differences in expression of BRD4 and MYC mRNA levels were found in JQ1‐resistant versus JQ1‐sensitive cell lines. By contrast, whereas HOXB4 and CCND2 mRNA levels were not detectable in JQ1‐sensitive KU812 cells, these WNT‐pathway targets were expressed in the JQ1‐resistant cell lines K562, KCL22, and KCL22^T315I^ (Figure S6A). Moreover, higher HOXB4 and CCND2 mRNA levels were detected in leukemic cells obtained from CML BP/AP patients compared to cells obtained from CML CP patients (Figure S6B). CCND2 and HOXB4 mRNA levels were also overexpressed in primary CML cells obtained from TKI‐resistant patients compared to CML cells obtained from TKI‐naïve patients (Figure S6C). Finally, we were able to demonstrate that JQ1‐resistant primary CML cells (IC_50:_ JQ1 > 750 nM) express higher MYC, CCND2, and HOXB4 mRNA levels compared to JQ1‐sensitive CML cells (IC_50_: <750 nM) (Figure S6D). These data confirm the important role of WNT signaling in BET inhibitor resistance in leukemic cells.^36^, ^37^

### 3.7 Effects of BET inhibition on survival of primary CML LSC

**FIGURE 3:**
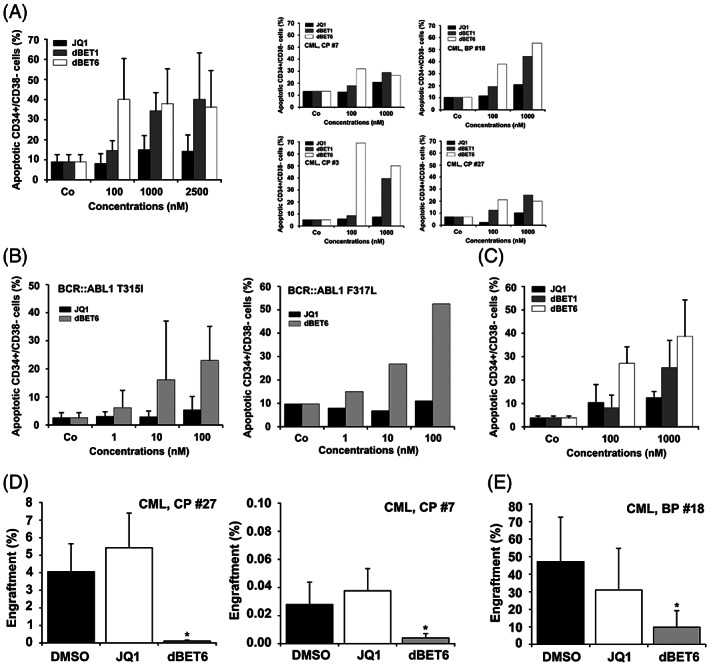
Effects of JQ1, dBET1 and dBET6 on survival and engraftment of CML LSC. (A) Primary CML MNC from three patients with CML CP and one with CML BP were incubated with various concentrations of JQ1, dBET1, or dBET6 at 37°C for 48 h. LSC were defined as CD34^+^/CD38^−^ cells and the percentage of Annexin V+ (apoptotic) cells were analyzed among DAPI‐negative cells by flow cytometry. Results represent the mean ± SD from four independent experiments (left panel). The right panels show single experiments in individual CML samples. (B) Primary CML MNC from three patients with BCR::ABL1 T315I+ CML (left panel) and one with BCR::ABL1 F317L+ CML (right panel) were incubated with various concentrations of JQ1 or dBET6 for 48 h. Then, the percentage of Annexin V+ LSC (CD34^+^/CD38^−^) among DAPI‐negative cells (apoptotic LSC) were analyzed by flow cytometry. Results in the left panel represent the mean ± SD from three independent experiments. (C) Normal BM MNC were incubated with various concentrations of JQ1, dBET1, or dBET6 at 37°C for 48 h. Thereafter, normal stem cells were defined as CD34^+^/CD38^−^ cells and the percentage of Annexin V positive cells (apoptotic cells) were analyzed among DAPI‐negative cells by flow cytometry. Results represent the mean ± SD from three independent experiments. (D) CD34^+^ CML CP cells were incubated in medium with DMSO (0.01%), 1 μM JQ1 or 1 μM dBET6 at 37°C for 4 h. Thereafter cells were harvested, washed and injected i.v. into NSG_SCF_ mice. After a maximum period of 6 months mice were sacrificed. BM was flushed and engraftment of human myeloid CD45^+^/CD33^+^/CD19^−^ cells determined by flow cytometry. Results are expressed as percentage of human engrafted myeloid cells and represent the mean ± SD from 4 to 5 mice per group. Asterisk: *p* < .05 compared to DMSO. E: T‐cell‐depleted CML BP MNC were incubated in medium containing DMSO (0.01%), 1 μM JQ1 or 1 μM dBET6 for 4 h. Then, cells were harvested, washed and injected i.v. into NSG mice. After 10 weeks mice were sacrificed. BM was flushed and engraftment of human CD45^+^ cells determined by flow cytometry. Results are expressed as percentage of human engrafted cells (CD45^+^) and represent the mean ± SD from 3 to 5 mice per group. Asterisk: *p* < .05 compared to DMSO.

JQ1 produced only little if any apoptosis in CD34^+^/CD38^−^ CML LSC suggesting that these cells are resistant (Figure 3A). However, the BET degraders dBET1 and dBET6 induced substantial apoptosis in CML LSC (Figure 3A). As expected, dBET6 was a more potent inhibitor of LSC survival compared to dBET1. Notably, dBET6 induced apoptosis in CML LSC in all samples tested, including one patient with BP (Figure 3A). Moreover, dBET6 induced apoptosis in CML LSC obtained from patients with TKI‐resistant CML exhibiting *BCR::ABL1* T315I or *BCR::ABL1* F317L (Figure 3B). dBET1 and dBET6 also induced apoptosis in normal CD34^+^/CD38^−^ bone marrow (BM) stem cells with slightly lower potency at 100 nM dBET6 compared to LSC (Figure 3C).

### 3.8 Degradation of BRD4 inhibits the ability of CML LSC to engraft in NSG mice

We next examined whether JQ1 and dBET6 interfere with engraftment of CML LSC in NSG_SCF_ mice (CP CML) or NSG mice (BP CML). Whereas pre‐incubation with JQ1 did not interfere substantially with engraftment of CP CML cells (at 6 months) or BP CML cells (at 10 weeks), dBET6 was found to block engraftment of CML LSC in all experiments (Figure 3D,E). These data suggest that BRD4 degradation by dBET6 is a potent approach to eliminate CML LSC.

### 3.9 Effects of BRD4‐targeting drugs on expression of MYC in CML cells

JQ1 was found to decrease the expression of MYC mRNA in KU812 cells (Figure S7A). Surprisingly, JQ1 also downregulated MYC mRNA expression in K562 cells as well as KCL22 and KCL22^T315I^ cells, although JQ1 did not induce growth‐inhibition in these cells (Figure S7A). Moreover, JQ1 decreased the expression of the MYC protein in K562, KCL22, KCL22^T315I^, and KU812 cells (Figure S7B,C). Corresponding results were obtained with OTX‐015 in KU812 and K562 cells (Figure S7D). JQ1 was also found to downregulate MYC in primary CML cells (Figures S7E,F). Finally, dBET1 and dBET6 decreased expression of MYC mRNA and protein in all cell lines tested (Figure S7A,C).

### 3.10 BRD4‐targeting drugs synergize with BCR::ABL1 TKI in inhibiting the proliferation of CML cells

Next, we asked whether BRD4‐targeting drugs can augment the effects of BCR::ABL1 TKI on CML cells. We found that JQ1 synergizes with all TKI in producing growth inhibition in KU812 cells (Figure S8A). Moreover, combined targeting by JQ1 and BCR::ABL1 TKI was found to overcome resistance against JQ1 in K562 cells (Figure S8A). Synergistic growth‐inhibitory effects in CML cell lines were also obtained when combining OTX‐015 with BCR::ABL1 TKI (Figure S8B). Furthermore, we were able to demonstrate synergistic anti‐neoplastic effects of JQ1 and BCR::ABL1 TKI in primary CML cells (Figure S8C). In addition, dBET6 was found to synergize with BCR::ABL1 TKI in producing growth inhibition in K562 cells (Figure S8D). Moreover, we were able to demonstrate that dBET6 synergizes with ponatinib in producing growth inhibition in KCL22^T315I^ cells (Figure S8E). Previous data suggested that combined inhibition of MEK, ERK‐, and MYC leads to cooperative growth‐inhibitory effects in CML cells.^42^ Therefore, we asked whether combined targeting of MEK and MYC would result in synergistic anti‐neoplastic effects. Indeed, our data show that combined inhibition of MEK and MYC leads to synergistic growth‐inhibitory effects in KU812 cells (Figure S9A,B). By contrast, no synergistic effects were observed when combining JQ1 with MEK inhibitors in the JQ1‐resistant cell line K562 (not shown).

### 3.11 BRD4‐targeting drugs override niche‐mediated TKI resistance in CML cells

**FIGURE 4:**
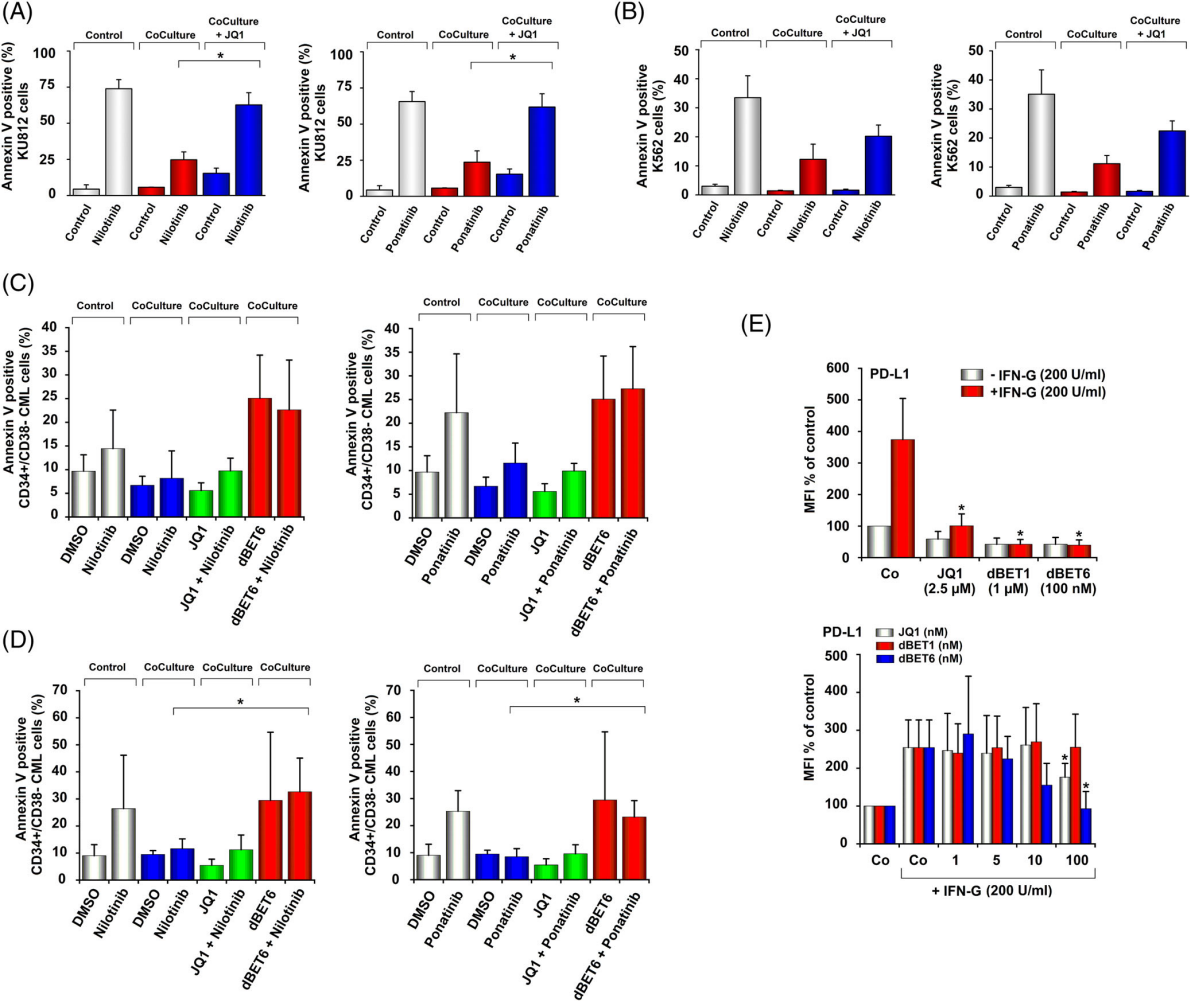
Effects of JQ1 on niche‐induced TKI‐resistance of CML cells and IFN‐G‐induced upregulation of PD‐L1. (A) and (B) KU812 cells and K562 cells were incubated in control medium (Control) or medium plus nilotinib (50 nM for KU812 cells, 100 nM for K562), ponatinib (10 nM), JQ1 (1 μM for KU812 and 2.5 μM for K562) or a combination of JQ1 and these TKI in the absence (Control) or presence (Coculture) of CAL‐72 cells at 37°C for 48 h. Thereafter, Annexin V+ cells were quantified among DAPI‐negative cells by flow cytometry. Results are expressed as Annexin V+ cells (%) and represent the mean ± SD from three experiments. Asterisk: *p* < .05 compared to TKI‐treated cells in co‐culture. C and D: Primary CML CP MNC were incubated in medium (+0.05% DMSO), nilotinib (5000 nM), ponatinib (500 nM), JQ1 (2500 nM), dBET6 (100 nM) or drug combinations (TKI + BET inhibitors) in the absence (Control) or presence (Coculture) of CAL‐72 cells (C) or primary osteoblasts (D) for 48 h. Thereafter, the percentages of CD34^+^/CD38^−^/Annexin V+ cells were measured among DAPI‐negative cells by flow cytometry. Results are expressed as Annexin V+ cells (%) and represent the mean ± SD from four independent experiments. Asterisk: *p* < .05 compared to TKI‐treated LSC in co‐culture. E: Primary CML CP MNC were incubated in control medium (Co) or medium containing 200 U/mL IFN‐G in the absence or presence of JQ1, dBET1 or dBET6 for 24 h. Then, CD34^+^/CD38^−^ LSC were analyzed for PD‐L1 expression by flow cytometry. Results are expressed as MFI (mean fluorescence intensity) in percent of control (Co without IFN‐G) and represent the mean ± SD from 3 (left panel) or 5 (right panel) independent experiments. Asterisk: *p* < .05 compared to IFN‐G treated control. [Color figure can be viewed at wileyonlinelibrary.com]

In the absence of osteoblasts, nilotinib and ponatinib induced apoptosis in KU812, K562, and primary CML LSC in most donors (Figure 4A–D). When cultured in the presence of CAL‐72 cells (osteoblast‐like osteosarcoma cell line) the effects of nilotinib and ponatinib on survival of KU812, K562, and CML LSC were no longer demonstrable suggesting niche‐induced resistance (Figure 4A–C). Addition of JQ1 partly restored the TKI effects in K562 cells, and completely restored TKI effects in KU812 cells in our co‐culture system (Figure 4A,B). However, unexpectedly, JQ1 was unable to restore TKI effects in primary LSC in these co‐cultures (Figure 4C). We next applied dBET6. As shown in Figure 4C, dBET6 was able to overcome osteoblast‐induced resistance against nilotinib and ponatinib in primary CML LSC. We also examined drug effects in co‐cultures containing primary osteoblasts. Again, osteoblasts were found to induce resistance in primary CML LSC, and dBET6 (but not JQ1) was found to overcome niche‐induced resistance (Figure 4D). dBET6 did not induce apoptosis in CAL‐72 cells in the presence or absence of CML LSC (Figure S10A) and, correspondingly, no growth‐inhibitory effects of 100 nM dBET6 were seen in CAL‐72 cells (Figure S10B). To study the mechanism of resistance induced by CAL‐72 cells in more detail, we analyzed mRNA levels of MYC, BRD4, and WNT signaling molecules in KU812 and K562 cells in the absence or presence of CAL‐72 cells by qPCR. However, we did not find major differences in expression levels of MYC, BRD4, HOXB4, and CCND2 mRNA when comparing purified CML cells (KU812, K562) obtained from cultures prepared with or without additional CAL‐72 cells (Figure S11).

### 3.12 BET‐targeting drugs inhibit interferon‐gamma (IFN‐G)‐induced expression of PD‐L1 in CML LSC

Finally, we examined resistance‐related molecules, including CD47 (IAP), CD243 (MDR‐1), and CD274 (PD‐L1) on CML LSC. Untreated CML LSC expressed CD47 and PD‐L1, but did not express MDR‐1 (Table S6). After incubation with IFN‐G, CML LSC expressed higher levels of PD‐L1 (Figure 4E, Table S6). JQ1 and both BET degraders suppressed IFN‐G‐induced expression of PD‐L1 in these cells (Figure 4E). By contrast, the BET inhibitors did not alter the expression of CD47 or MDR‐1 in CML LSC (Figure S12). IFN‐G failed to induce expression of CD47 or MDR‐1 in CML cells (Figure S12, Table S6). Other checkpoint molecules, including CD28 (TP44), CD86 (B7‐2), CD273 (PD‐L2), CD279 (PD‐1), and CD366 (TIM3) were not detected on resting or IFN‐G‐exposed CML LSC (Table S6) and IFN‐G failed to induce expression of any of these checkpoint molecules in KU812 or K562 cells (Table S6).

## 4 DISCUSSION

In most patients with Ph^+^ CML the disease can be kept under control with imatinib or with second‐ or third generation BCR::ABL1 TKI.^4^, ^5^, ^6^, ^7^, ^8^, ^9^, ^10^, ^11^, ^12^, ^13^, ^14^, ^15^, ^16^, ^18^, ^19^, ^20^ However, despite the availability of novel potent TKI, many patients develop resistance and progress to AP and BP which remains a challenge in clinical hematology. One critical point is that CML LSC exhibit multiple forms of drug resistance.^26^, ^27^, ^28^, ^43^, ^44^, ^45^ During the past few years, a number of attempts have been made to detect novel promising therapeutic targets in CML (stem) cells and to overcome TKI resistance with new drugs.^27^, ^28^, ^29^, ^30^ We here show that the epigenetic reader BRD4 and its downstream effector MYC are expressed in CML cells, including LSC, and that both targets mediate cell growth and LSC resistance in CML cells. Moreover, we provide evidence that the next generation BRD4 degrader dBET6 overcomes multiple forms of drug resistance in CML LSC.

We and others have shown that BRD4 is expressed in neoplastic cells and serves as a therapeutic target in AML.^34^, ^35^ In this study, we show that BRD4 is expressed in CML cells, including CML cell lines and primary CML cells. However, the levels of BRD4 mRNA varied in the CML cell lines examined, with higher levels found in KU812 compared to K562 and KCL22 cells. As expected, the BRD4‐downstream target MYC was expressed in primary CML cells and in all CML cell lines tested, with higher MYC mRNA levels found in KU812 cells than in K562 cells.

So far, it is unknown how BRD4‐ and MYC‐expression is regulated in CML (stem) cells. We found that BCR::ABL1 TKI downregulate the expression of MYC mRNA in K562, KCL22, and KU812 cells. As expected, only ponatinib, but not the other TKI tested, was found to downregulate expression of MYC mRNA levels in KCL22^T315I^ cells. These data suggest that BCR::ABL1 is involved in the regulation of MYC expression in CML cells. Next, we examined BCR::ABL1‐downstream molecules, including MEK and the PI3‐kinase‐AKT–mTOR pathway.^46^, ^47^, ^48^ We found that several MEK inhibitors, including refametinib and trametinib, decrease MYC expression in CML cells whereas the PI3‐kinase/mTOR blocker BEZ235 showed no effects.

Previous data have shown that BRD4 serves as a novel therapeutic target in AML.^34^, ^35^ In the current study, we found that BRD4 shRNA induce growth‐inhibition in KU812 and K562 cells and that the BRD4‐targeting drugs JQ1 and OTX‐015 suppress proliferation of KU812 and primary CML cells. The effects of these drugs were dose‐dependent, but IC_50_ values varied from donor to donor, and in some patients, CML cells appeared to be largely resistant. Interestingly, CML cells obtained from CP CML patients usually showed a good response, whereas in a majority of patients with BP, leukemic cells appeared to be resistant. Similarly, KU812, a cell line exhibiting multi‐lineage differentiation, showed a good response to JQ1, whereas no effect of JQ1 was seen in the more immature cell line K562 as well as in KCL22 and KCL22^T315I^ cells.

So far, little is known about the mechanisms of resistance of leukemic cells against BRD4 inhibitors.^36^, ^37^ We found that KU812 cells express higher levels of BRD4‐ and MYC mRNA compared to K562 cells. These data suggest that the increased sensitivity of KU812 cells against JQ1 may be related to more abundant target expression. On the other hand, the JQ1‐resistant cell lines KCL22 and KCL22^T315I^ cells also expressed substantial amounts of MYC. An alternative explanation would be that K562 and KCL22 cells are more capable of upregulating WNT signaling genes involved in rapid restoration of MYC expression and thus resistance against JQ1.^36^, ^37^ To test this hypothesis, we examined two critical WNT pathway‐associated genes, HOXB4 and CCND2. The observation that these two genes were only detectable in JQ1‐resistant CML cell lines but not in KU812 cells confirm the hypothesis that the WNT pathway is involved in resistance against JQ1. In line with this assumption, JQ1‐resistant primary CML cells displayed higher HOXB4 and CCND2 mRNA levels compared to JQ1‐responsive cells.

To overcome the resistance of CML cells against JQ1 and other conventional BET inhibitors, we followed two strategies. First, we applied drug combinations and found that JQ1 and OTX‐015 exert synergistic growth‐inhibitory effects on CML cells when combined with BCR::ABL1 TKI. These effects were not only seen in KU812, but also in JQ1‐resistant K562 cells. In addition, we found that JQ1 and BCR::ABL1 TKI can produce cooperative growth‐inhibitory effects in primary CML cells. This is of particular interest as such novel agents, including birabresib (OTX‐015) are currently being tested for anti‐neoplastic effects in clinical trials in patients solid tumors, multiple myeloma, and acute leukemia. In the context of CML, we believe that one reasonable approach would be to examine the effects of these drugs alone and in combination with BCR::ABL1 TKI in TKI‐resistant non‐transplantable patients or in patients who have drug‐resistant CML BP.

Next, we applied two BRD4 degraders, dBET1 and dBET6.^38^, ^39^ Both degraders were found to induce growth inhibition in primary CML cells in all samples tested, including BP cells. In these experiments, dBET6 was a more potent drug compared to dBET1 or JQ1. A similar observation was made in various CML cell lines. In all cell lines tested, dBET6 produced clear growth‐inhibitory effects whereas no substantial effects were seen with dBET1. These data confirm previously published results on lymphatic leukemia cells.^39^ In this study, the differential anti‐leukemic potency of dBET1 and dBET6 is explained by improved cellular target engagement of dBET6, likely caused by elevated membrane permeability.^39^

A number of previous and more recent studies have shown that LSC are a critical target of therapy.^12^, ^43^, ^44^, ^45^, ^49^, ^50^ In fact, drugs and drug combinations may only exert curative effects when eliminating or completely suppressing most or all LSC in a given neoplasm. Therefore, we were interested to see whether BET‐targeting drugs can exert apoptosis‐inducing effects on CD34^+^/CD38^−^ CML LSC. In a first step, we applied JQ1 and found that this drug does not induce apoptosis in LSC. However, the two BET degraders applied (dBET1 and dBET6) were found to induce apoptosis in CML LSC *in vitro*. In all patients tested, dBET6 was the more potent drug. Apoptosis‐inducing effects of dBET6 on LSC were seen in patients with CP CML, BP CML, and TKI‐resistant CML cells expressing *BCR::ABL1* T315I or *BCR::ABL1* F317L. These data suggest that dBET6 also overcomes acquired, mutation‐induced, resistance. Finally, pre‐incubation of CML cells with dBET6 resulted in an almost complete depletion of NSG‐engrafting LSC in CP samples and a major decrease in engraftment of BP cells in NSG mice. Together, these data suggest that dBET6 is a potent inhibitor of growth and survival of LSC.

A number of studies suggest that niche cells contribute to resistance of CML LSC.^28^, ^51^, ^52^ We found that primary osteoblasts and/or the osteoblast‐like osteosarcoma cell line CAL‐72 induce TKI resistance in CML LSC and in the CML cell lines KU812 and K562. In these cell lines, JQ1 was found to overcome niche‐induced resistance against nilotinib and ponatinib. However, JQ1 was unable to overcome niche‐mediated TKI resistance in primary CML LSC. Therefore, we applied dBET6. Indeed, the more potent BET degrader dBET6 was found to overcome osteoblast‐mediated resistance of CML LSC against nilotinib and ponatinib. So far, the mechanisms of osteoblast‐induced TKI resistance and how dBET6 overcomes resistance remains unknown. One possible explanation could be that dBET6 is able to alter niche cell function and their ability to protect CML cells from TKI effects. Whether this protective effect of osteoblasts on CML (stem) cells is mediated via factors influencing (stabilizing) the BRD4‐MYC pathway remains unknown. In the current study, we were not able to show direct growth‐inhibitory effects of dBET6 on CAL‐72 cells.

Several different studies have shown that various surface antigens detectable on CML (stem) cells mediate TKI resistance.^43^, ^53^ These surface antigens include, among others, CD47 (IAP), MDR‐1 (CD243), and the checkpoint PD‐L1 (CD274). We examined the expression of these antigens on CML LSC. In these experiments, IFN‐G augmented the expression of PD‐L1 on CML LSC which confirmed previous data published by Hogg et al.^54^ We also found that JQ1 and the other BRD4‐targeting drugs tested, including the BRD4 degraders dBET1 and dBET6, inhibit IFN‐G‐induced upregulation of this checkpoint‐target in CML LSC. This was an expected results, as *PD‐L1* is a target gene of BRD4.^54^

In summary, our data show that CML cells express BRD4 and MYC and that these molecules may serve as potential new therapeutic targets in TKI‐resistant CML. We also show that the second‐generation BET degrader dBET6 overcomes multiple mechanisms of LSC resistance in CML. Moreover, IFN‐G‐induced PD‐L1 expression in CML LSC was found to be disrupted by BET inhibition or BET degradation. Whether these observations can be exploited and translated *in vivo* to patients with TKI‐resistant CML remains unknown at present.

## AUTHOR CONTRIBUTIONS

Barbara Peter, Thomas Rülicke and Peter Valent designed research. Barbara Peter, Gregor Eisenwort, Irina Sadovnik, Karin Bauer, Michael Willmann, Thomas Rülicke, Daniela Berger, Gabriele Stefanzl, Georg Greiner, Gregor Hoermann, and Alexandra Keller performed research. Georg E. Winter, Thomas Hoffmann, Johannes Zuber contributed vital new reagents. Dominik Wolf, Martin Čulen, and Jiří Mayer collected data. Barbara Peter, Ana‐Iris Schiefer, Wolfgang R. Sperr, and Peter Valent analyzed and interpreted data. Barbara Peter and Peter Valent wrote the manuscript.

## FUNDING INFORMATION

This study was supported by the Austrian Science Fund (FWF): SFB grants F4701‐B20, F4704‐B20, F4710‐B20 and the Herzfelder'sche Familienstiftung: P30625‐B28. The research was funded in part by Celgene Corporation, Summit, NJ via a research grant. The authors independently developed, directed, and are fully responsible for all content for this manuscript.

## CONFLICT OF INTEREST

Peter Valent received honoraria from Celgene/BMS, Pfizer, Novartis, Incyte, Blueprint, and AOP Orphan and research grants from Celgene/BMS, and AOP Orphan. Gregor Hoermann received a research grant from Novartis, honoraria from Novartis, Roche, Beckman Coulter, Pfizer, Celgene, and Bristol‐Myers Squibb. Jiří Mayer received a research grant from Novartis and travel support from Novartis. Wolfgang R. Sperr received honoraria from AbbVie, Amgen, Celgene, Daiichi Sankyo, Deciphera, Incyte, Jazz, Novartis, Pfizer, Thermo Fisher, and travel support from Pfizer and Roche. Dominik Wolf received research grants from Bristol‐Myers Squibb‐Celgene and Novartis. The other authors have no other conflict of interest in this study.

## Supporting information


**Appendix S1.** Supporting information.

## Data Availability

The data that support the findings of this study are available from the corresponding author upon reasonable request.
