# Supplementary material for: BRD4 degradation blocks expression of MYC and multiple forms of stem cell resistance in Ph+ chronic myeloid leukemia
Source: Am J Hematol. 2022 Jul 18;97(9):1215–25. doi: 10.1002/ajh.26650 (PMC9546315; doi:10.1002/ajh.26650)
Supplement: Supplementary file 1 — Appendix S1. Supporting information. [file AJH-97-1215-s001.doc]

Supplemental Information to Peter et al.:

**BRD4 Degradation Blocks Expression of MYC and Multiple Forms of Stem Cell Resistance in Ph+ Chronic Myeloid Leukemia**

Barbara Peter, Gregor Eisenwort, Irina Sadovnik, Karin Bauer,

Michael Willmann, Thomas Rülicke, Daniela Berger, Gabriele Stefanzl, Georg Greiner, Gregor Hoermann, Alexandra Keller, Dominik Wolf,

Martin Čulen, Georg E. Winter, Thomas Hoffmann, Ana-Iris Schiefer,

Wolfgang R. Sperr, Johannes Zuber, Jiří Mayer, Peter Valent

**Supplemental Material and Methods**

**Reagents**

Imatinib, nilotinib, dasatinib, dBET1, and the MEK inhibitors PD0325901 and refametinib (RDEA119) were purchased from ChemieTek (Indianapolis, IN, USA). The BET inhibitors OTX-015 and JQ1, the MEK inhibitor trametinib, ponatinib and the PI3-kinase inhibitor BEZ235 were purchased from SelleckChem (Houston, TX, USA). dBET6 was purchased from Aobious (Gloucester, MA, USA) and bosutinib was purchased from LC Laboratories (Woburn, MS). Stock solutions of drugs were prepared by dissolving in DMSO (Sigma-Aldrich, St. Louis, MO, USA). RPMI 1640 medium was purchased from Lonza (Verviers, Belgium), fetal calf serum (FCS) and DMEM medium from Gibco life technologies (Gaithersburg, MD, USA), ^3^H-thymidine from Perkin Elmer (Boston, MA, USA), 4′,6‐diamidino‐2‐phenylindole (DAPI) and propidium iodide (PI) from Sigma-Aldrich. Interferon-gamma (IFN-G) was obtained from Roche Diagnostics (Mannheim, Germany).

**Cell lines**

KU812 cells were kindly provided by Dr. Kenji Kishi (Niigata University, Niigata, Japan), and K562 were kindly provided by Dr. Michael Deininger (University of Utah, Salt Lake City, UT). KCL22 and CAL-72 cells were purchased from Leibniz-Institute DSMZ, German Collection of Microorganism and Cell Cultures (Braunschweig, Germany). KCL22, KU812 and K562 cells were maintained in RPMI 1640 medium with 10% FCS. The osteoblast-like osteosarcoma cell line CAL-72 was kept in DMEM medium with 10% FCS. Primary osteoblasts were purchased from PromoCell (Heidelberg, Germany) and kept in osteoblast growth medium (PromoCell). Cell lines have been authenticated by the DSMZ using the Authentication of Human Cell Lines Service. Cell lines were periodically tested for mycoplasma contamination by conventional PCR using the Venor GeM Classic Mycoplasma Detection Kit (Minerva Biolabs, Berlin, Germany). KCL22 cells harboring *BCR::ABL1*^T315I^ (KCL22^T315I^) were generated by culturing with imatinib and dasatinib essentially as described previously.^1^ Ba/F3 cells containing *BCR::ABL1*^WT^ (Ba/F3 BCR::ABL1^WT^) or *BCR::ABL1*^T315I^ (Ba/F3 BCR::ABL1^T315I^) were kindly provided by Dr. Michael Deininger.^2^ KCL22^T315I^ and Ba/F3 cells were maintained in RPMI 1640 medium plus 10% FCS and KCL22^T315I^ cells were kept in the presence of 5 µM imatinib.

**Isolation of primary CML cells**

Peripheral blood (PB) and/or bone marrow (BM) cells of patients with CML (chronic phase, CP, n=26; accelerated phase, AP, n=4; blast phase, BP, n=13), were obtained during routine investigations. BCR::ABL1 mRNA levels of CML patients were quantified according to the International Scale (IS) for standardized quantification as reported.^3-6^ The patients´ characteristics are summarized in Supplemental Table S1. In control experiments, normal BM cells from lymphoma patients without BM involvement (n=3) were used. Heparinized PB or BM cells were layered over Ficoll to isolate mononuclear cells (MNC). Patient samples were stored in a local biobank. All patients gave written informed consent before PB or BM samples were collected. The study was approved by the ethics committee of the Medical University of Vienna and conducted in accordance with the declaration of Helsinki.

**Enrichment of CD34+ cells and T cell-depleted MNC by magnetic-activated cell sorting (MACS)**

In samples from 3 CML CP patients, leukocytes were enriched for CD34+ cells by MACS using a CD34 MicroBead Kit from Miltenyi Biotec (Bergisch Gladbach, Germany) before being injected into NSG_SCF_ mice or before leukemic stem cells (LSC) were purified by cell sorting for qPCR experiments. As assessed by flow cytometry the purity of the CD34+ fractions was >95%. In one BP sample, CML MNC were T cell-depleted by MACS using CD3 MicroBeads (Miltenyi Biotec) before being injected into NSG mice. T cell depletion was confirmed by flow cytometry. MACS was performed according to the instructions of the manufacturer (Miltenyi Biotec).

**Detection of mRNA by quantitative real-time PCR (qPCR)**

For qPCR analysis, RNA was isolated from cell lines, primary CML cells, or purified (sorted) CD34^+^/CD38^−^ CML LSC using the RNeasy MinElute Clean Up Kit, RNeasy Plus Micro Kit or RNeasy Plus Mini Kit (Qiagen, Hilden, Germany). cDNA was synthesized using Moloney murine leukemia virus reverse transcriptase and random primers (both from Invitrogen, Carlsbad, CA). Primers specific for *BRD4*, *MYC, GUSB, HOXB4, CCND2* or *GAPDH* (Supplemental Table S2) were applied. qPCR was performed on a QuantStudio 3 Real-Time PCR System (Thermo Fisher Scientific, Waltham, MA, USA) using iTaq Universal SYBR Green Supermix (Bio-Rad, Hercules, CA, USA) and plasmid standards.^7-9^ MYC mRNA copy numbers, HOXB4 copy numbers, CCND2 copy numbers and BRD4 mRNA copy numbers were normalized to GUSB mRNA or GAPDH mRNA copy numbers and expressed as percent of GUSB mRNA or GAPDH mRNA. In a separate set of experiments, primary CML cells or cell lines were incubated in medium or medium containing various concentrations of JQ1 (0.1-5 µM), dBET1, dBET6 (each 0.5-5 µM), or the BCR-ABL tyrosine kinase inhibitors (TKI) imatinib, nilotinib, dasatinib, bosutinib, or ponatinib (each 1 µM) at 37°C for 24 hours before being analyzed for expression of BRD4 and/or MYC mRNA expression levels by qPCR.

**Detection of BRD4 and MYC in CML cells by immunocytochemistry (ICC) and immunohistochemistry (IHC)**

ICC was performed on cytospin-slides prepared with KU812 cells, K562 cells and primary CML MNC, essentially as described.^7-9^ Cytospin slides were incubated with a polyclonal rabbit anti-human BRD4 antibody (Sigma-Aldrich; work-dilution: 1:100 for cell lines and 1:50 for primary cells) or a monoclonal rabbit anti-MYC (Y69) antibody (Abcam, Cambridge, MA, USA, work dilution 1:100 for cell lines and 1:50 for primary cells) for 20 hours. Slides were then washed and incubated with biotinylated goat-anti-rabbit IgG (Biocare Medical, Pacheco, CA, USA) for 30 minutes. Then, cells were incubated with streptavidin-conjugated alkaline phosphatase (Biocare Medical, Pacheco, CA, USA) for 30 minutes. Antibody-reactivity was made visible by using New Fuchsin Substrate Kit (Histofine, Nichirei Bioscience Inc., Tokyo, Japan). IHC was performed on sections prepared from paraffin-embedded, formalin-fixed, BM biopsy specimens using the indirect immunoperoxidase staining technique as reported.^7-9^ Prior to staining, BM sections were pretreated by microwave oven. Slides were incubated with anti-BRD4 antibody (dilution: 1:100) or anti-MYC antibody (dilution: 1:50) at 4°C for 20 hours. Then, slides were washed and incubated with biotinylated anti-rabbit IgG (Vector, Burlingame, CA, USA) for 30 minutes, washed, and then exposed to Vectastain ABC KIT (30 minutes). AEC was used as substrate. A specification of antibodies is provided in Supplemental Table S3. ICC and IHC slides were examined using an Olympus DP21 camera connected to an Olympus BX50F4 microscope (Olympus Corporation; Shinjuku, Tokyo, Japan) equipped with 60x/0.90 UPlanFL (IHC) or 100x/1.35 UPlanAPO (Oil Iris; ICC) objective lenses. Images were adjusted by Adobe Photoshop CS5 (Adobe Systems, San Jose, CA, USA). Antibodies used in ICC and IHC experiments are shown in Supplemental Table S3.

**Western blot experiments**

CML cells were incubated in medium or in medium containing various concentrations of JQ1, OTX-015, dBET1, dBET6, imatinib, nilotinib, dasatinib, bosutinib, ponatinib, BEZ235, trametinib, PD0325901 or refametinib (RDEA119) (0.01-5 µM) at 37°C for 24 hours. Thereafter, cells were harvested and Western blotting was performed essentially as described^9^ using a monoclonal anti-MYC antibody (D83C12, Cell Signaling, Danvers, MA, USA) and anti-β-Actin antibody or anti-Actin antibodies (all from Santa Cruz Technology, Santa Cruz, CA, USA). A detailed description of antibodies is provided in Supplemental Table S3. Antibody reactivity was made visible with a donkey anti-rabbit IgG, a sheep anti-mouse IgG (both from GE Healthcare, Buckinghamshire, UK), or a donkey anti-goat IgG (Santa Cruz Technology) and ECL Plus Western Blotting Substrate (Thermo Scientific, Rockford, IL, USA). Western Blot experiments using cell lines were performed at least two times.

**shRNA-mediated knockdown of BRD4 and MYC in CML cells**

shRNAs targeting MYC were cloned into SGEP, a lentiviral vector that co-expresses shRNAs from the miR-E backbone linked to the green fluorescent protein (GFP).^9^ For knock-down experiments of BRD4, pRRL-SFFV-GFP-mir-E was constructed based on pRRL-SGEP^10^ by removing the PGK-Puro cassette.^8^ Renilla luciferase (Ren.713) was used in the respective vectors as a control shRNA. Sequences of shRNA are shown in Supplemental Table S4. VSV-G pseudotyped lentiviral particles were produced and transduced into KU812 and K562 cells as previously described.^8,9^ After transduction (8 days) of KU812 cells and K562 cells with shRNA directed against BRD4, GFP+ cells were purified by flow cytometry and cell sorting. Untransduced KU812 and K562 cells were also run through the sorter to provide equal conditions for both cell fractions. Knockdown of *BRD4* was confirmed by qPCR. Subsequently, transduced and untransduced cells were mixed at 1:1 and cultured for 7 days. Then, the percentage of GFP+ cells was measured by flow cytometry at defined time intervals (days 0, 2, 5 and 7). *MYC* knockdown was confirmed by qPCR in GFP-sorted cells. Mixing experiments were performed using unsorted MYC shRNA-transduced cells and unsorted untransduced cells at a GFP rate of 50% (50% shRNA-transduced cells present) and cultured for 7 days. The percentage of GFP+ cells was measured by flow cytometry at defined time intervals (days 0, 2, 5 and 7).

**^3^H-thymidine uptake experiments and evaluation of apoptosis in CML cells**

For ^3^H-thymidine uptake experiments, CML cell lines, and primary CML MNC were incubated in medium or in medium containing various concentrations of JQ1 (1-10,000 nM), dBET1 (1-10,000 nM), dBET6 (1-10,000 nM), or OTX-015 (10-10,000 nM) at 37°C for 48 hours. Then, ^3^H-thymidine was added for 16 hours and ^3^H-thymidine uptake was measured as described previously.^7-9^ To determine cooperative drug-effects, CML cells were incubated medium or medium containing various concentrations of BRD4 blockers alone or in combination with BCR::ABL1 TKI (imatinib, nilotinib, dasatinib, bosutinib, or ponatinib) at a fixed ratio of drug concentrations. In another set of experiments, JQ1 or dBET6 was applied alone or in combination with the MEK inhibitors refametinib or PD0325901 at a fixed ratio of drug concentrations. Drug combination activities (synergistic, antagonistic) were determined by calculating combination index (CI) values using Calcusyn software (Ferguson, MO).^9^ In a separate set of experiments, CAL-72 were incubated in medium or medium containing various concentrations of dBET6 (1-1,000 nM) at 37°C for 48 hours. Then, ^3^H-thymidine uptake was measured as described previously.^7-9^ All ^3^H-thymidine uptake experiments were performed in triplicates. For apoptosis evaluation, KU812, K562, KCL22 and KCL22^T315I^ cells were incubated in medium or in medium containing various concentrations of JQ1, dBET1, dBET6, or OTX-015 (0.5-5 µM) at 37°C for 48 hours. Then, apoptotic cells were quantified by Annexin V/PI staining (KU812 and K562 cells) and flow cytometry essentially as reported.^7,9^ In brief, cells were washed, resuspended in Annexin V binding buffer, and incubated with FITC-labeled Annexin V (eBioscience, Vienna, Austria) for 15 minutes. Then, cells were washed and 4 µL PI were added to each tube. Apoptotic cells were defined as Annexin V positive cells. The percentages of Annexin V positive cells were determined by flow cytometry on a BD FACSCalibur (BD Biosciences, San Jose, CA, USA). For evaluating apoptosis in KCL22 and KCL22^T315I^ cells, Annexin V/DAPI staining was performed. In brief, cells were harvested after drug exposure, washed in PBS and incubated with FITC-labeled Annexin V (eBioscience) for 15 minutes. Thereafter, cells were washed and DAPI (1 µg/mL) was added. Apoptotic cells were defined as Annexin V positive cells. The percentages of Annexin V positive cells were determined by flow cytometry on a FACSCanto (BD Biosciences, San Jose, CA, USA). In addition, apoptosis of CD34^+^/CD38^−^ CML LSC as well as normal CD34^+^/CD38^−^ stem cells was determined after culturing CML MNC or normal BM MNC in the absence or presence of JQ1, dBET1 and dBET6 (1-2500 nM) for 48 hours by Annexin V/DAPI staining and multi-color flow cytometry on a FACSCantoII (BD Biosciences). Cells were gated on the CD45^dim^/CD34^+^/CD38^−^ population and the percentages of Annexin V positive cells among the DAPI negative cells were analyzed by flow cytometry. Antibodies used are shown in Table Supplemental S3.

**Mouse xenotransplantation experiments**

NSG mice (NOD.Cg-*Prkdc^scid^ Il2rg^tm1Wjl^*) and NSG_SCF_ mice exhibiting a 220 amino acid isoform of human membrane-bound SCF (NOD.Cg-*Prkdc^scid^ Il2rg^tm1Wjl^* Tg[PGK1-KITLG*220] 441Daw/SzJ) were purchased from The Jackson Laboratory (Bar Harbor, ME, USA) and housed in individual ventilated cages to maintain pathogen-free conditions. Experiments were performed on adult (7.5-12.5 week-old) female and male mice. Littermates of the same sex were randomly assigned to experimental groups. Primary CML cells (MACS-isolated CD34^+^ cells from two patients with CP CML and T cell-depleted MNC from one patient with BP CML (each 1-1.5 million cells per mouse) were pre-incubated in medium containing DMSO (0.01%) or medium containing JQ1 (1 µM) or dBET6 (1 µM) at 37°C for 4 hours. Thereafter cells were washed and then injected into the tail veins of NSG mice (BP) or NSG_SCF_ mice (CP). Before injection, cell viability was checked by trypan blue exclusion and was always greater than 90%. Twenty-four hours prior to injection, mice were sub-lethally irradiated (2.4 Gy). After injection, mice were inspected daily and sacrificed when they showed disease symptoms or after a maximum period of 6 months for CP samples or 10 weeks for BP sample. BM cells were obtained from flushed femurs, tibias and humeri. Engrafted cells were analyzed by flow cytometry using mAb against human CD45, CD33 and CD19. Antibodies used are shown in Supplemental Table S3. All animal studies were approved by the ethics committee of the Veterinary Medicine Vienna and carried out in accordance with guidelines for animal care and protection and protocols approved by Austrian law (BMWFW-68.205/0113-WF/V/3b/2016).

**Co-culture experiments with osteoblast-like cells and CML cells**

Niche-related cells can protect CML LSC from TKI effects and thus contribute to LSC resistance. We asked whether BRD4-targeting drugs can counteract niche-related resistance in CML cells. In initial experiments, K562 and KU812 cells were co-cultured on osteoblast-like CAL-72 osteosarcoma cells in RPMI 1640 medium with 10% FCS at 37°C. Co-cultured cells were incubated in medium or in medium containing nilotinib (100 nM or 50 nM) or ponatinib (10 nM) as single agent or in combination with JQ1 (2.5 µM or 1 µM) at 37°C for 48 hours. After incubation, cells were harvested and the percentages of apoptotic (Annexin V positive cells among DAPI negative cells) K562 cells (defined as CD44^−^ cells) or KU812 cells (defined as CD45^+^ cells) were determined by flow cytometry. The effects of JQ1 and the TKI applied on K562 cells (alone), and KU812 cells (alone) were examined in parallel. In a next step, primary CML MNC were co-cultured with CAL-72 cells or primary osteoblasts. In these experiments, the combinations ´nilotinib (5 µM) plus JQ1 (2.5 µM)´, ´ponatinib (0.5 µM) plus JQ1 (2.5 µM)´, ´nilotinib (5 µM) plus dBET6 (100 nM)´, and ´ponatinib (0.5 µM) plus dBET6 (100 nM)´, or DMSO (0.05%) as well as single agents were applied at 37°C for 48 hours. Primary CML MNC were gated on the CD45^dim^/CD34^+^/CD38^−^ population (LSC) and the percentages of Annexin V positive cells among the DAPI negative cells were analyzed by flow cytometry. The effects of BET inhibitors and the TKI applied on primary CML LSC were examined in parallel. Moreover, we examined the percentage of apoptotic CAL-72 cells cultured separately or cultured together with CML LSC in our co-culture system. In these experiments, cells were cultured in medium or in medium containing dBET6 (100 nM). In co-cultures, CAL-72 cells were defined as CD45^−^ cells. Antibodies used to detect LSC and cell lines are shown in Supplemental Table S3. In a separate set of experiments KU812 cells or K562 cells were co-cultured with CAL-72 cells at 37°C for 48 hours. Thereafter, the two cell types were separated (purified) by cell sorting on a FACSAria Fusion (BD Biosciences) as reported.^11-14^ In the co-cultures containing CAL-72 cells and KU812 cells, CAL-72 were defined as CD33^–^ cells and KU812 defined as CD33^+^ cell. In the co-cultures containing CAL-72 and K562 cells, CAL-72 cells were defined as CD44^+^ cells and K562 as CD44^–^ cells. The purity of sorted cells was >90% in all experiments. Antibodies used for cell sorting are provided in Supplemental Table S3. Control cells cultured separately in the same experiments were also sorted in order to provide equal conditions. After sorting, expression of MYC, BRD4, CCND2, and HOXB4 mRNA was analyzed by qPCR.

**Evaluation of expression of resistance-related checkpoint molecules on CML LSC**

Recent data suggest that cytokine-induced expression of certain checkpoint molecules, including PD-L1, is associated with stem cell resistance against various targeted drugs. We examined whether BRD4 inhibitors can counteract interferon-gamma (IFN-G) induced expression of PD-L1 on CML LSC. Primary CML MNC were incubated in medium or medium containing IFN-G (200 U/ml) in the absence or presence of JQ1 (1-2500 nM), dBET1 (1-1000 nM), or dBET6 (1-100 nM) at 37°C for 24 hours. Then, cells were harvested and CD34^+^/CD38^−^ CML stem cells were examined for expression of CD47 (IAP), CD243 (MDR-1) and CD274 (PD-L1) by flow cytometry essentially as described previously.^15^ In a separate set of experiments, primary CML MNC, K562 cells and KU812 cells were incubated in medium or medium containing IFN-G (200 U/ml) at 37°C for 24 hours. Then cells were harvested and CD34^+^/CD38^−^ CML stem cells and CML cell lines were examined for the expression of CD28, CD47, CD80, CD83, CD86, CD243, CD273, CD274, CD279 and CD366 by flow cytometry as described.^14,15^ A specification of antibodies is provided in Supplemental Table S3.

**References**

1. Yuan H, Wang Z, Gao C, et al. BCR-ABL gene expression is required for its mutations in a novel KCL-22 cell culture model for acquired resistance of chronic myelogenous leukemia. *J Biol Chem.* 2010;285(7):5085-5096.

2. La Rosee P, Corbin AS, Stoffregen EP, Deininger MW, Druker BJ. Activity of the Bcr-Abl kinase inhibitor PD180970 against clinically relevant Bcr-Abl isoforms that cause resistance to imatinib mesylate (Gleevec, STI571) *Cancer Res*. 2002;62 (24):7149–7153.

3. Gabert J, Beillard E, van der Velden VH, et al. Standardization and quality control studies of 'real-time' quantitative reverse transcriptase polymerase chain reaction of fusion gene transcripts for residual disease detection in leukemia - a Europe Against Cancer program. *Leukemia.* 2003;17(12):2318-2357.

4. Foroni L, Wilson G, Gerrard G, et al. Guidelines for the measurement of BCR-ABL1 transcripts in chronic myeloid leukaemia. *Br J Haematol.* 2011;153(2):179-190.

5. Baccarani M, Deininger MW, Rosti G, et al. European LeukemiaNet recommendations for the management of chronic myeloid leukemia: 2013. *Blood.* 2013;122(6):872-884.

6. Cross NC, White HE, Colomer D, et al. Laboratory recommendations for scoring deep molecular responses following treatment for chronic myeloid leukemia. *Leukemia.* 2015;29(5):999-1003.

7. Herrmann H, Blatt K, Shi J, et al. Small-molecule inhibition of BRD4 as a new potent approach to eliminate leukemic stem- and progenitor cells in acute myeloid leukemia AML. *Oncotarget.* 2012;3(12):1588-1599.

8. Wedeh G, Cerny-Reiterer S, Eisenwort G, et al. Identification of bromodomain-containing protein-4 as a novel marker and epigenetic target in mast cell leukemia. *Leukemia.* 2015;29(11):2230-2237.

9. Peter B, Bibi S, Eisenwort G, et al. Drug-induced inhibition of phosphorylation of STAT5 overrides drug resistance in neoplastic mast cells. *Leukemia.* 2018;32(4):1016-1022.

10. Fellmann C, Hoffmann T, Sridhar V, et al. An optimized microRNA backbone for effective single-copy RNAi. *Cell Rep.* 2013;5(6):1704-1713.

11. Herrmann H, Sadovnik I, Cerny-Reiterer S, et al. Dipeptidylpeptidase IV (CD26) defines leukemic stem cells (LSC) in chronic myeloid leukemia. *Blood.* 2014;123(25):3951-3962.

12. Hauswirth AW, Florian S, Printz D, et al. Expression of the target receptor CD33 in CD34+/CD38-/CD123+ AML stem cells. *Eur J Clin Invest.* 2007;37(1):73-82.

13. Herrmann H, Kneidinger M, Cerny-Reiterer S, et al. The Hsp32 Inhibitors SMA-ZnPP and PEG-ZnPP Exert Major Growth-Inhibitory Effects on CD34+/CD38+ and CD34+/CD38- AML Progenitor Cells. *Curr Cancer Drug Targets.* 2012;12(1):51-63.

14. Herrmann H, Cerny-Reiterer S, Gleixner KV, et al. CD34+/CD38- stem cells in chronic myeloid leukemia express Siglec-3 (CD33) and are responsive to the CD33-targeting drug gemtuzumab/ozogamicin. *Haematologica.* 2012;97(2):219-226.

15. Hogg SJ, Vervoort SJ, Deswal S, et al BET-Bromodomain Inhibitors Engage the Host Immune System and Regulate Expression of the Immune Checkpoint Ligand PD-L1. *Cell Rep.* 2017;18(9):2162-2174.

**Supplemental Tables**

Supplemental Table S1

**Patients´ characteristics**

| Patient No. | Gender | Age | Source | Diagnosis | *BCR::ABL1* mutations | Leucocytes (x10^9^/L) | Platelets (x10^9^/L) | Hemoglobin (g/dL) | blasts (%) PB | blasts (%) BM | *BCR::ABL1* (%) PB | treatment before sampling |
| --- | --- | --- | --- | --- | --- | --- | --- | --- | --- | --- | --- | --- |
| 1 | f | 80 | PB | CML-CP | n.t. | 96.3 | 477 | 10.5 | 0 | 1 | 33.912 | none |
| 2 | f | 62 | PB | CML-CP | n.t. | 101.73 | 302 | 11.8 | 0 | 3 | 47.871 | none |
| 3 | m | 49 | PB | CML-CP | n.t. | 168.73 | 1027 | 8.9 | 2 | 1 | 38.733 | none |
| 4 | m | 19 | PB | CML-CP | n.t. | 175.8 | 282 | 8.7 | 2 | 1 | 51.578 | none |
| 5 | f | 37 | PB | CML-CP | n.t. | 27.33 | 412 | 12.7 | 0 | <1 | 22.198 | none |
| 6 | f | 28 | PB | CML-CP | n.t. | 108.57 | 432 | 8.5 | 0 | 1 | 11.664* | none |
| 7 | f | 46 | PB | CML-CP | n.t. | 570.5 | 342 | 7.5 | 1 | 2 | 47.54 | none |
| 8 | f | 56 | PB | CML-CP | n.d. | 68.72 | 818 | 12.6 | 0 | 1 | 25.678* | none |
| 9 | m | 58 | PB | CML-CP | n.t. | 247.61 | 282 | 11.2 | 2 | 1 | 43.083 | none |
| 10 | m | 63 | PB | CML-CP | n.t. | 103.59 | 438 | 8.6 | 5 | n.t. | 35.527 | none |
| 11 | f | 34 | PB | CML-CP | n.t. | 396.93 | 425 | 7.2 | 2 | 1 | 43.84 | none |
| 12a | m | 48 | PB | CML-CP | n.t. | 315.92 | 190 | 10.8 | 2 | n.t. | 39.403 | none |
| 12b | m | 49 | PB | CML-BP | T315I | 24.2 | 93 | 7.5 | 37 | 56 | 69.655 | imatinib (res), dasatinib (dis), nilotinib (dis) |
| 13 | m | 34 | BM | CML-CP | n.t. | 98.11 | 266 | 15.2 | 0 | <1 | 39.148 | none |
| 14 | m | 59 | PB | CML-CP | n.t. | 86.42 | 266 | 13.5 | 1 | 2-3 | 29.993* | none |
| 15 | m | 50 | BM/PB | CML-CP | n.t. | 146.2 | 163 | 16.2 | 1 | 1 | 39.142 | none |
| 16 | m | 62 | PB | CML-CP | n.t. | 93.5 | 305 | 13.1 | 1 | 1 | 39.993 | none |
| 17a | m | 48 | BM | CML-BP | G250E | 2.6 | 158 | 11.9 | 11 | 35 | 25.074* | imatinib (res), dasatinib (res), bosutinib (res), ponatinib |
| 17b | m | 48 | PB | CML-BP | G250E E255V | 33.53 | 200 | 7.6 | 77 | 65 | 33.427 | imatinib (res), dasatinib (res), bosutinib (res), ponatinib (res) |
| 18 | m | 61 | PB | CML-BP | n.t. | 36.79 | 12 | 8.9 | 63 | 73 | 37.403 | imatinib (dis) |
| 19 | f | 54 | PB | CML-CP | n.t. | 428.23 | 899 | 7.9 | 3 | 2 | 34.71 | none |
| 20 | f | 80 | BM | CML-CP | n.t. | 33.34 | 311 | 12.2 | 0 | 1 | 33.751 | imatinib (dis) |
| 21 | f | 53 | BM | CML-AP | n.d. | 3.19 | 20 | 9.8 | 0 | <1 | 58.598 | imatinib (res), nilotinib (res), dasatinib (res), bosutinib (res),  ponatinib |
| 22 | f | 58 | BM | CML-CP | n.t. | 57.32 | 465 | 14.1 | 0 | 1 | 68.668 | none |
| 23 | m | 67 | BM | CML-CP | n.t. | 72.35 | 320 | 12.6 | 1 | 2 | 37.262* | none |
| 24 | f | 29 | BM/PB | CML-CP | n.t. | 30.74 | 439 | 13.9 | 0 | <1 | 44.375 | none |
| 25 | f | 69 | PB | CML-CP | n.t. | 95.36 | 867 | 11.9 | 2 | 1 | 50.955 | none |
| 26 | f | 56 | PB | CML-CP | n.t. | 398.94 | 165 | 8.6 | 1 | 1 | 31.626 | none |
| 27 | f | 25 | PB | CML-CP | n.t. | 506.92 | 197 | 7.9 | 9 | n.t. | 46.743 | none |
| 28 | f | 32 | PB | CML-CP | n.t. | 38.69 | 732 | 13.2 | 1 | 1 | 25.676 | none |
| 29 | f | 66 | PB | CML-AP | n.t. | 30.14 | 293 | 7.9 | 2 | 4 | 60.654 | none |
| 30 | m | 67 | PB | CML-CP | n.t. | 228.36 | 140 | 12.2 | 3 | 1 | 65.063 | none |
| 31 | m | 31 | BM | CML-BP | T315I | 55.8 | 134 | 5.58 | 67 | 82.6 | 69.000 | imatinib (dis), dasatinib (res) |
| 32 | f | 48 | BM | CML-BP | T315I | 39.6 | 86.1 | 9.42 | 79 | 62 | 100.000 | dasatinib (res) |
| 33 | m | 56 | BM | CML-BP | F317L | 4.49 | 316 | 13.8 | 15 | 42.8 | 52.000 | imatinib (dis), dasatinib (res) |
| 34 | m | 71 | PB | CML-AP | T315I | 264.81 | 792 | 8.1 | 10 | n.t. | n.t. | imatinib (res)  hydroxyurea |
| 35 | f | 43 | PB | CML-AP | n.t. | 19.78 | 580 | 10.6 | 18 | 19 | 69.958 | none |
| 36 | m | 42 | PB | CML-BP | n.d. | 44.56 | 769 | 9.0 | 20 | 20-25 | 63.490* | none |
| 37a | f | 38 | BM | CML-BP | n.d. | 4.85 | 298 | 6.4 | 8 | 30 | 48.668* | dasatinib (res), imatinib (res), nilotinib (res), bosutinib (res) |
| 37b | f | 38 | PB | CML-BP | n.t. | 3.53 | 18 | 9.6 | 52 | n.t. | n.t | dasatinib (res), imatinib (res), nilotinib (res), bosutinib (res),  CHT |
| 38a | m | 41 | PB | CML-BP | n.d. | 9.18 | 1 | 7.6 | 35 | 90 | 27.240 | imatinib (res),  nilotinib (dis),  dasatinib (res) |
| 38b | m | 43 | PB | CML-BP | V299L  F317L | 39.75 | 21 | 11.6 | 81 | 50 | 45.640* | imatinib (res),  nilotinib (dis), dasatinib (res),  CHT+GO  HSCT |
| 39 | f | 28 | PB | CML-BP | n.d. | 16.14 | 236 | 10.6 | 35 | 45 | 46.641 | imatinib (res), dasatinib (res) |
| 40 | f | 48 | BM | CML-BP | G250E | 73 | 29 | 9.2 | 28 | n.t. | 64.967** | imatinib (res) |
| 41 | m | 46 | BM | CML-BP | n.t. | 12.73 | 103 | 7.5 | 22 | 26 | 40*/** | imatinib(res) |
| 42 | m | 64 | BM | CML-BP | G250E | 264.81 | 118 | 8.7 | 47 | 62 | 70.93** | imatinib (res) |

Abbreviations: f, female; m, male; PB, peripheral blood; BM, bone marrow; CP, chronic phase; AP, accelerated phase; BP, blast phase; n.t., not tested; n.d., not detected; res, resistant; dis, discontinued; CHT, polychemotherapy; GO, gemtuzumab ozogamycin; HSCT, hematopoietic stem cell transplantation. *In these cases, the percentage of BCR::ABL1 mRNA was measured in the BM. **In these samples, BCR::ABL1 mRNA levels were not corrected according to IS.

Supplemental Table S2

**Primer sequences used for quantitative real time PCR**

--------------------------------------------------------------------------------------------------------

Gene Sequence

--------------------------------------------------------------------------------------------------------

*MYC* 5’-TGCTCCATGAGGAGACACC-3’ (forward)

5’-CCTGCCTCTTTTCCACAGAA-3’ (reverse)

*BRD4* 5’-GCCCGCAAGCTCCAGGATGT-3’ (forward)

5’-CCTCAGGCTCGTCCGGCATC -3’ (reverse)

*GUSB*  5’-GGGCTTCGAGGAGCAGTGG-3’ (forward)

5’- GCTGGAGGGAACTGGCATGT-3’ (reverse)

*GAPDH* 5’-CGAGCCACATCGCTCAGACA-3’ (forward)

5’-GGCGCCCAATACGACCAAAT-3’ (reverse)

*HOXB4* 5’-CTGGATGCGCAAAGTTCAC-3’ (forward)

5’-GTGTAGGCGGTCCGAGAG-3’ (reverse)

*CCND2* 5’GTGCAGAAGGACATCCAACC-3’ (forward)

5’TCGCACTTCTGTTCCTCACA-3’ (reverse)

--------------------------------------------------------------------------------------------------------

PCR, polymerase chain reaction.

Supplemental Table S3

**List of antibodies used in this study**

| CD | antigen | clone | conjugate | source* | catalog number |
| --- | --- | --- | --- | --- | --- |
| CD3 | TcR | UCHT1 | APC | BD Biosciences | 555335 |
| CD19 | B4 | SJ25C1 | APC | eBioscience | 17-0198-42 |
| CD28 | Tp44, T44 | CD28.2 | PE | BioLegend | 302908 |
| CD33 | Siglec-3 | WM53 | PE | BD Biosciences | 555450 |
| CD34 | HPCA-1 | 581 | FITC | BioLegend | 343504 |
| CD34 | HPCA-1 | 581 | Pacific Blue | BioLegend | 343512 |
| CD34 | HPCA-1 | 581 | PE | BD Biosciences | 555822 |
| CD38 | T10 | HIT2 | APC | BD Biosciences | 555462 |
| CD44 | Hermes | 515 | PE | BD Biosciences | 550989 |
| CD45 | LCA | 2D1 | APC-H7 | BD Biosciences | 560178 |
| CD45 | LCA | HI30 | V500 | BD Biosciences | 560779 |
| CD45 | LCA | HI30 | APC-Cy7 | BioLegend | 304014 |
| CD45 | LCA | 2D1 | PerCP | BD Biosciences | 345809 |
| CD47 | IAP | BH612 | PE | BD Biosciences | 556046 |
| CD80 | B7-1 | 2D10 | PE | BioLegend | 305208 |
| CD83 | HB15 | HB15e | PE | BioLegend | 305308 |
| CD86 | B7-2 | IT2.2 | PE | BioLegend | 305406 |
| CD243 | MDR-1 | 15D3 | PE | BD Biosciences | 340555 |
| CD273 | PD-L2 | MIH18 | PE | BioLegend | 345506 |
| CD274 | PD-L1 | 29E.2A3 | PE | BioLegend | 329706 |
| CD279 | PD-1 | EH12.2H7 | PE | BioLegend | 329906 |
| CD366 | TIM3 | F38-2E2 | PE | BioLegend | 345006 |
| n.c. | BRD4 | polyclonal | none | Sigma-Aldrich | HPA015055 |
| n.c. | MYC | Y69 | none | abcam | ab32072 |
| n.c. | MYC | D84C12 | none | Cell Signaling | 5605 |
| n.c. | Actin | 2Q1055 | none | Santa Cruz Biotechnology | sc-58673 |
| n.c. | beta-Actin | polyclonal | none | Santa Cruz Biotechnology | sc-130656 |
| n.c. | Actin | polyclonal | none | Santa Cruz Biotechnology | sc-1616 |

*Antibodies were purchased from eBioscience (San Diego, CA, USA), BioLegend (San Diego, CA, USA), BD Biosciences (San Jose, CA, USA), Sigma-Aldrich (St. Louis, MO, USA), abcam (Cambridge, UK), Cell Signaling (Danvers, MA, USA) and Santa Cruz Biochtechnology (Santa Cruz, CA, USA). Abbreviations: n.c., not (yet) clustered; APC, allophycocyanin; PE, phycoerythrin; FITC, fluorescein isothiocyanate; PerCP, peridinin-chlorophyll-protein.

Supplemental Table S4

**97-mer sequences of shRNAs used in knockdown experiments**

--------------------------------------------------------------------------------------------------------

BRD4.602

TGCTGTTGACAGTGAGCGACAGGACTTCAACACTATGTTTTAGTGAAGCCACAGATGTAAAACATAGTGTTGAAGTCCTGGTGCCTACTGCCTCGGA

BRD4.1817

TGCTGTTGACAGTGAGCGACAGCAGAACAAACCAAAGAAATAGTGAAGCCACAGATGTATTTCTTTGGTTTGTTCTGCTGGTGCCTACTGCCTCGGA

MYC.1834 TGCTGTTGACAGTGAGCGCACGACGAGAACAGTTGAAACATAGTGAAGCCACAGATGTATGTTTCAACTGTTCTCGTCGTTTGCCTACTGCCTCGGA

Ren.713

TGCTGTTGACAGTGAGCGCAGGAATTATAATGCTTATCTATAGTGAAGCCACAGATGTATAGATAAGCATTATAATTCCTATGCCTACTGCCTCGGA

--------------------------------------------------------------------------------------------------------

shRNA, short hairpin RNA; Ren, Renilla luciferase.

Supplemental Table S5

**Efficacy of JQ1, OTX-015, dBET1 and dBET6 in CML patient samples**

| **Patient** | **JQ1 (µM)*** | **OTX-015 (µM)*** | **dBET1 (µM)*** | **dBET6 (µM)*** |
| --- | --- | --- | --- | --- |
| 1 | 0.1-0.5 | n.t. | n.t. | n.t. |
| 2 | 0.1-0.5 | n.t. | n.t. | n.t. |
| 3 | 0.75-1 | n.t. | n.t. | n.t. |
| 4 | 1 - 5 | n.t. | n.t. | n.t. |
| 5 | 0.1-0.5 | n.t. | n.t. | n.t. |
| 6 | 0.05-0.1 | n.t. | n.t. | n.t. |
| 7 | 0.1-0.25 | n.t. | 0.1-0.25 | <0.01 |
| 8 | 0.1-0.25 | 0.1-0.25 | n.t. | n.t. |
| 9 | 0.1-0.5 | 0.1 | n.t. | n.t. |
| 10 | 0.1 | 0.01-0.05 | n.t. | n.t. |
| 11 | 0.1-0.5 | 0.1-0.25 | n.t. | n.t. |
| 12a | 0.1-0.5 | 0.1-0.5 | n.t. | n.t. |
| 12b | 1 - 5 | n.t. | 0.01-0.1 | 0.001-0.005 |
| 13 | 0.5-0.75 | > 10 | n.t. | n.t. |
| 14 | 1 - 5 | 1 - 5 | n.t. | n.t. |
| 15 | 0.5-0.75 | 1 | n.t. | n.t. |
| 16 | 0.05-0.1 | 0.05-0.1 | n.t. | n.t. |
| 17a | n.t. | n.t. | n.t. | n.t. |
| 17b | > 5 | > 5 | 0.1-0.5 | 0.001-0.005 |
| 18 | n.t. | n.t. | n.t. | n.t. |
| 19 | 0.05-0.1 | 0.1-0.5 | n.t. | n.t. |
| 20 | 0.01-0.1 | 0.1-0.5 | 0.1-0.5 | 0.001-0.005 |
| 21 | 0.1-0.5 | 0.01-0.1 | 0.1 | 0.001-0.005 |
| 22 | 0.01-0.1 | 0.01-0.1 | 0.5-1 | 0.01-0.1 |
| 23 | 1 - 5 | > 5 | 0.1-0.5 | 0.005-0.01 |
| 24 | 0.1-0.5 | 0.1-0.5 | 0.1-0.5 | 0.001-0.005 |
| 25 | 1 | 1 - 5 | 0.1-1 | 0.005-0.01 |
| 26 | 0.1-0.5 | 0.1-0.5 | 0.1-0.5 | 0.001-0.005 |
| 27 | n.t. | n.t. | n.t. | n.t. |
| 28 | n.t. | n.t. | n.t. | n.t. |
| 29 | n.t. | n.t. | n.t. | n.t. |
| 30 | n.t. | n.t. | n.t. | n.t. |
| 31 | n.t. | n.t. | n.t. | n.t. |
| 32 | n.t. | n.t. | n.t. | n.t. |
| 33 | n.t. | n.t. | n.t. | n.t. |
| 34 | n.t. | n.t. | n.t. | n.t. |
| 35 | 1 - 5 | n.t. | 0.1-0.5 | 0.001-0.005 |
| 36 | 0.01-0.1 | n.t. | 0.01-0.1 | 0.001-0.005 |
| 37a | n.t. | n.t. | n.t. | n.t. |
| 37b | 0.1-0.5 | n.t. | 0.1-0.5 | 0.001-0.005 |
| 38a | n.t. | n.t. | n.t. | n.t. |
| 38b | n.t. | n.t. | n.t. | n.t. |
| 39 | 0.1-0.5 | n.t. | n.t. | 0.001-0.005 |
| 40 | 1 - 5 | n.t. | n.t. | 0.01-0.1 |
| 41 | 1 - 5 | n.t. | n.t. | 0.005-0.01 |
| 42 | n.t. | n.t. | n.t. | n.t. |

* IC_50_ values were determined by ^3^H-thymidine uptake; n.t., not tested.

Supplemental Table S6

**Surface expression of checkpoint related proteins with and without IFN-G**

| **Surface Marker** | **KU812**  **-IFN-G** | **KU812**  **+IFN-G** | **K562**  **-IFN-G** | **K562**  **+IFN-G** | **CML LSC**  **-IFN-G** | **CML LSC**  **+IFN-G** |
| --- | --- | --- | --- | --- | --- | --- |
| CD28 (TP44) | - | - | - | - | - | - |
| CD47 (IAP) | +++ | +++ | ++ | ++ | ++ | ++ |
| CD80 (B7-1) | ++ | ++ | +/- | +/- | - | - |
| CD83 (HB15) | +/- | +/- | +/- | +/- | +/- | - |
| CD86 (B7-2) | +/- | +/- | - | - | - | +/- |
| CD243 (MDR-1) | +/- | +/- | - | - | - | - |
| CD274 (PD-L1) | + | ++ | + | + | + | ++ |
| CD273 (PD-L2) | - | - | - | - | - | - |
| CD279 (PD-1) | - | - | +/- | +/- | - | - |
| CD366 (TIM3) | +/- | +/- | +/- | +/- | - | - |

Expression of immunological checkpoint molecules on KU812 cells, K562 cells and primary CD34^+^/CD38^─^ CML cells (LSC) with or without exposure to IFN-G (200 U/ml) for 24 hours. The staining index (SI) was calculated using MFI of the test antibody divided by MFI obtained with an isotype-matched control antibody. Results in the table represent the mean SI values calculated from all experiments and expression levels were graded according to the following score: -, SI <1.3; +/-, SI 1.31.-3; +, SI 3.01-10; ++, SI 10.01-100; +++, SI >100. Expression of CD47, CD243, and CD274 on LSC was examined in 6 patients with CML CP and 1 with CML AP. The other markers (on LSC) were analyzed in 2 patients with CML CP and 1 with CML AP. Abbreviation: LSC, leukemic stem cells; CML, chronic myeloid leukemia; IFN-G, interferon-gamma; MFI, mean fluorescence intensity; SI, staining index; CP, chronic phase; AP, accelerated phase.

**Supplemental Figures**

Supplemental Figure S1


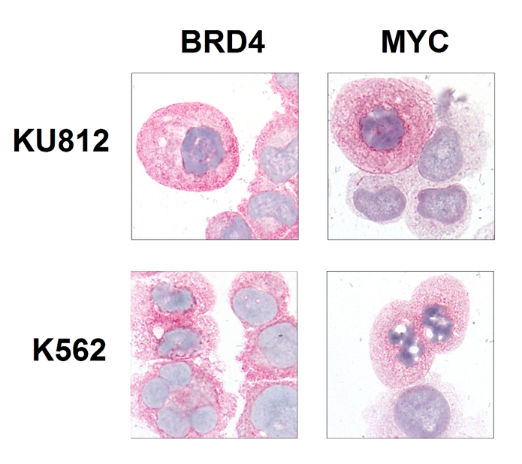


Peter et al., Supplemental Figure S1A


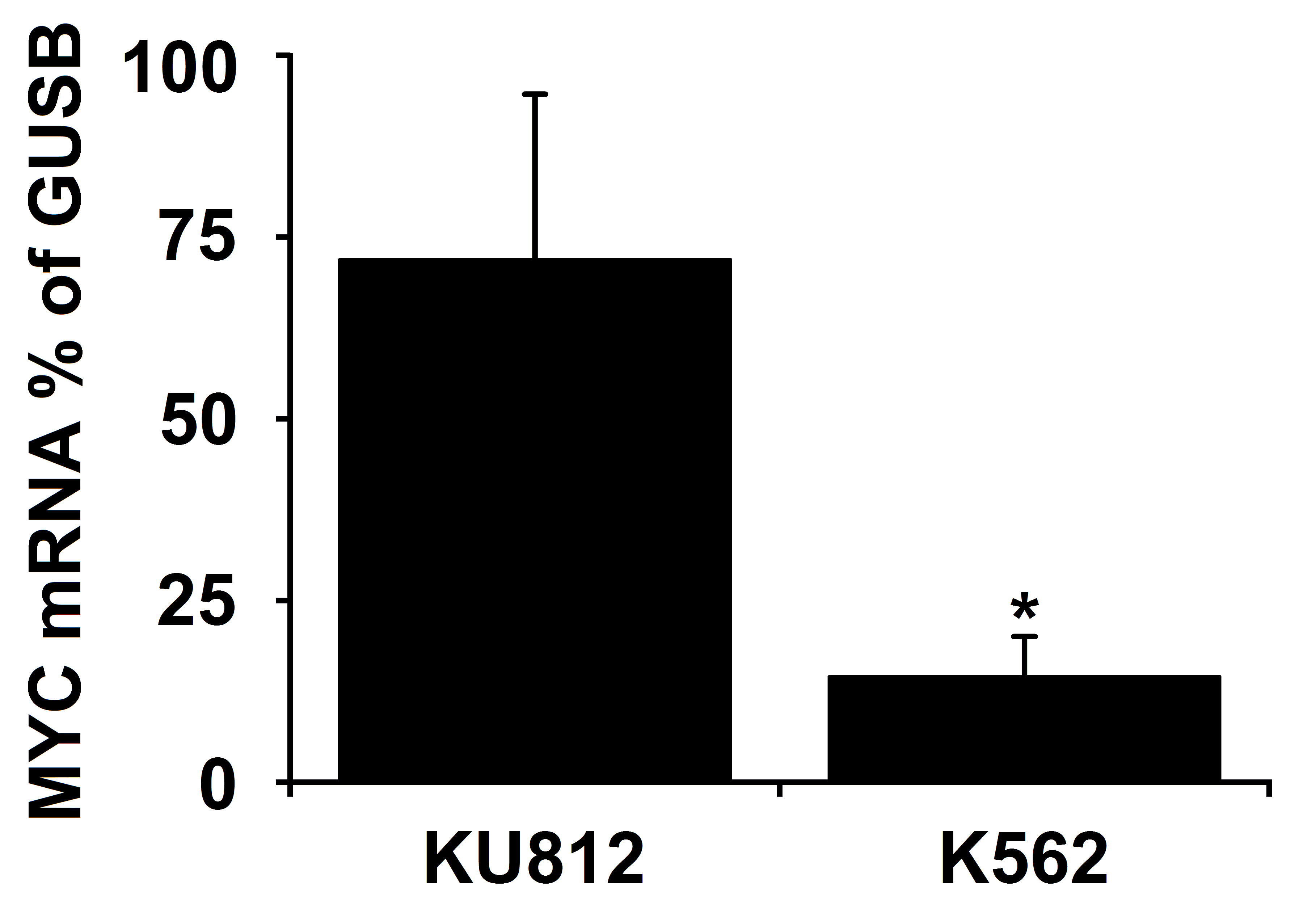

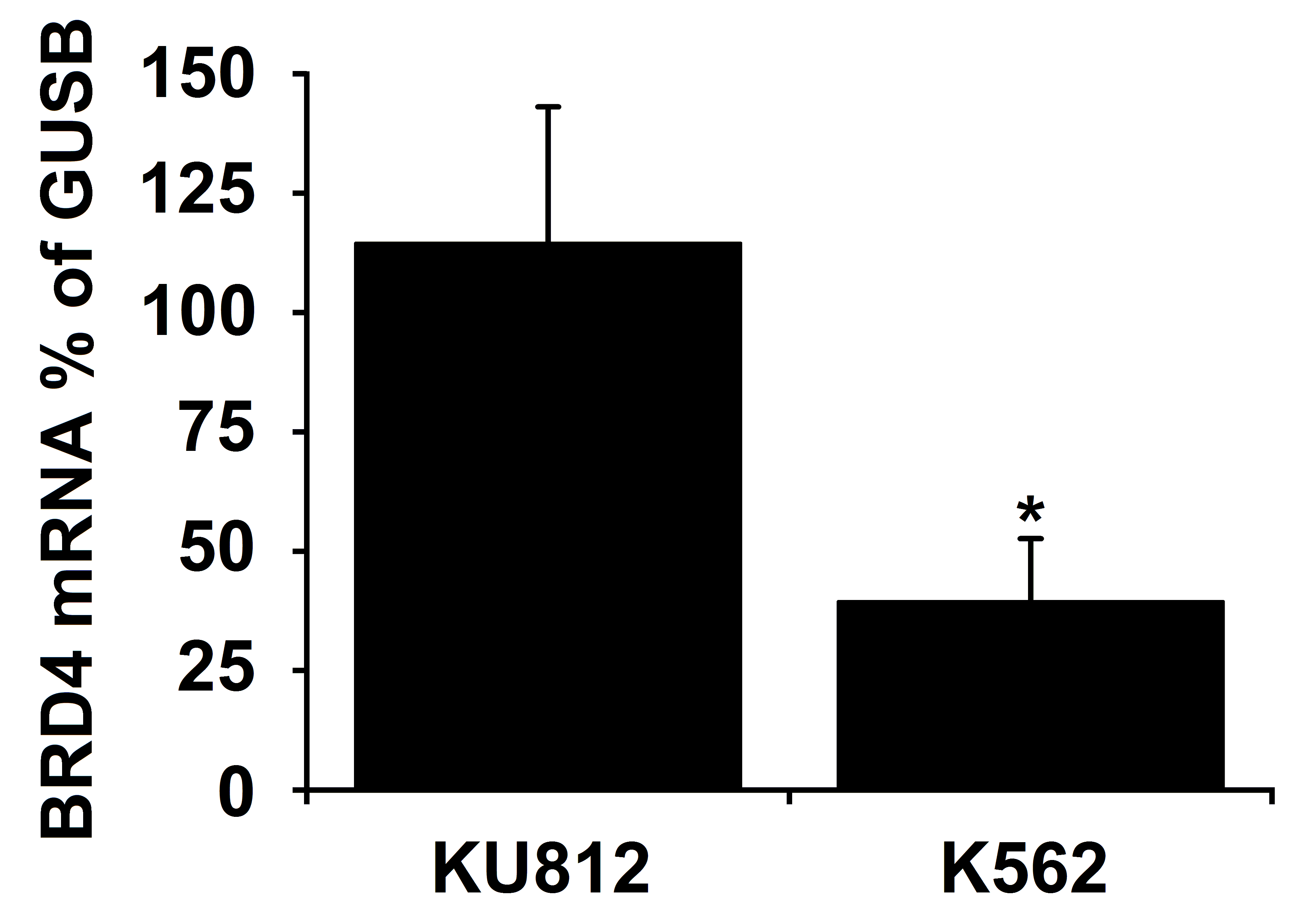


Peter et al., Supplemental Figure S1B


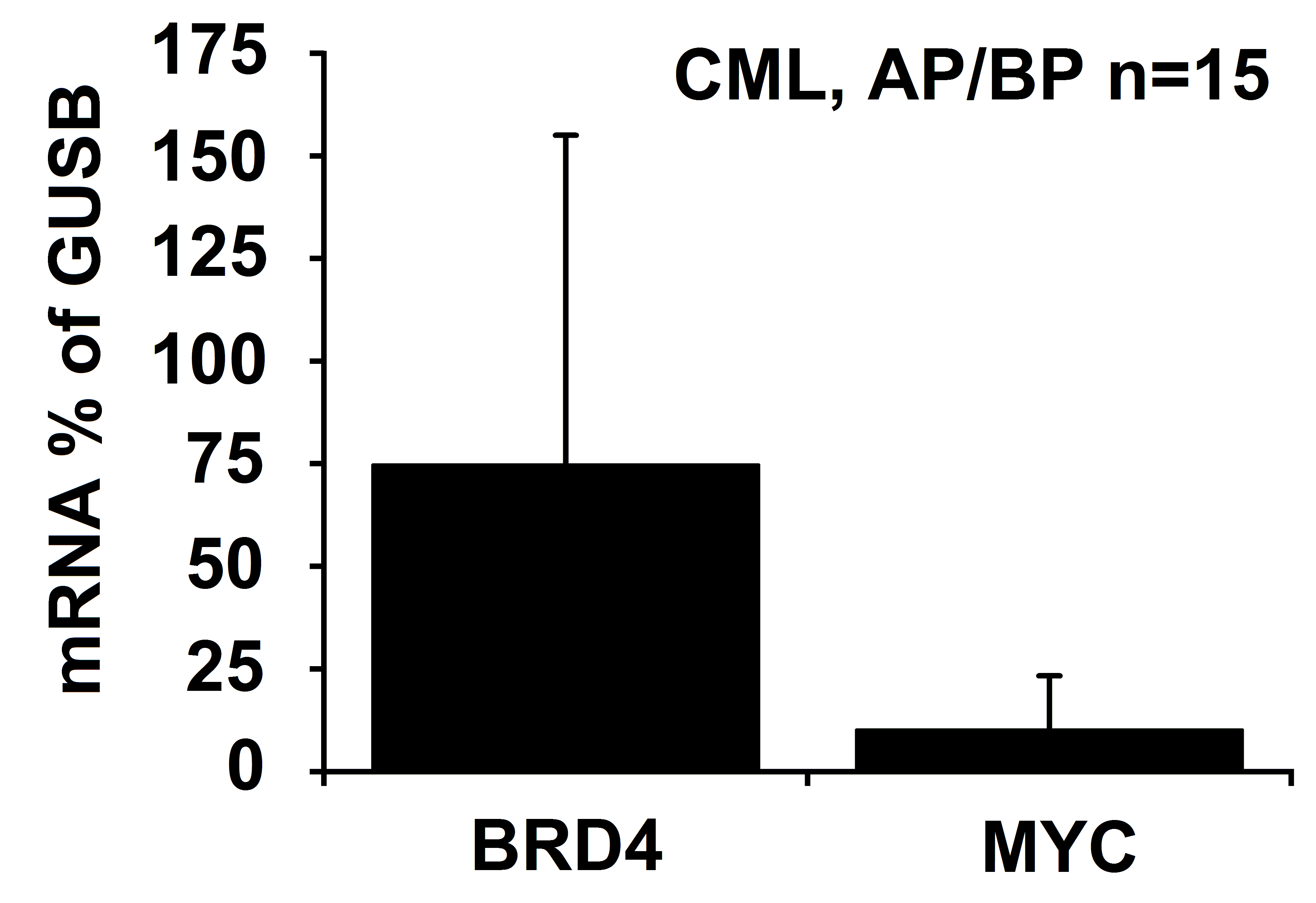

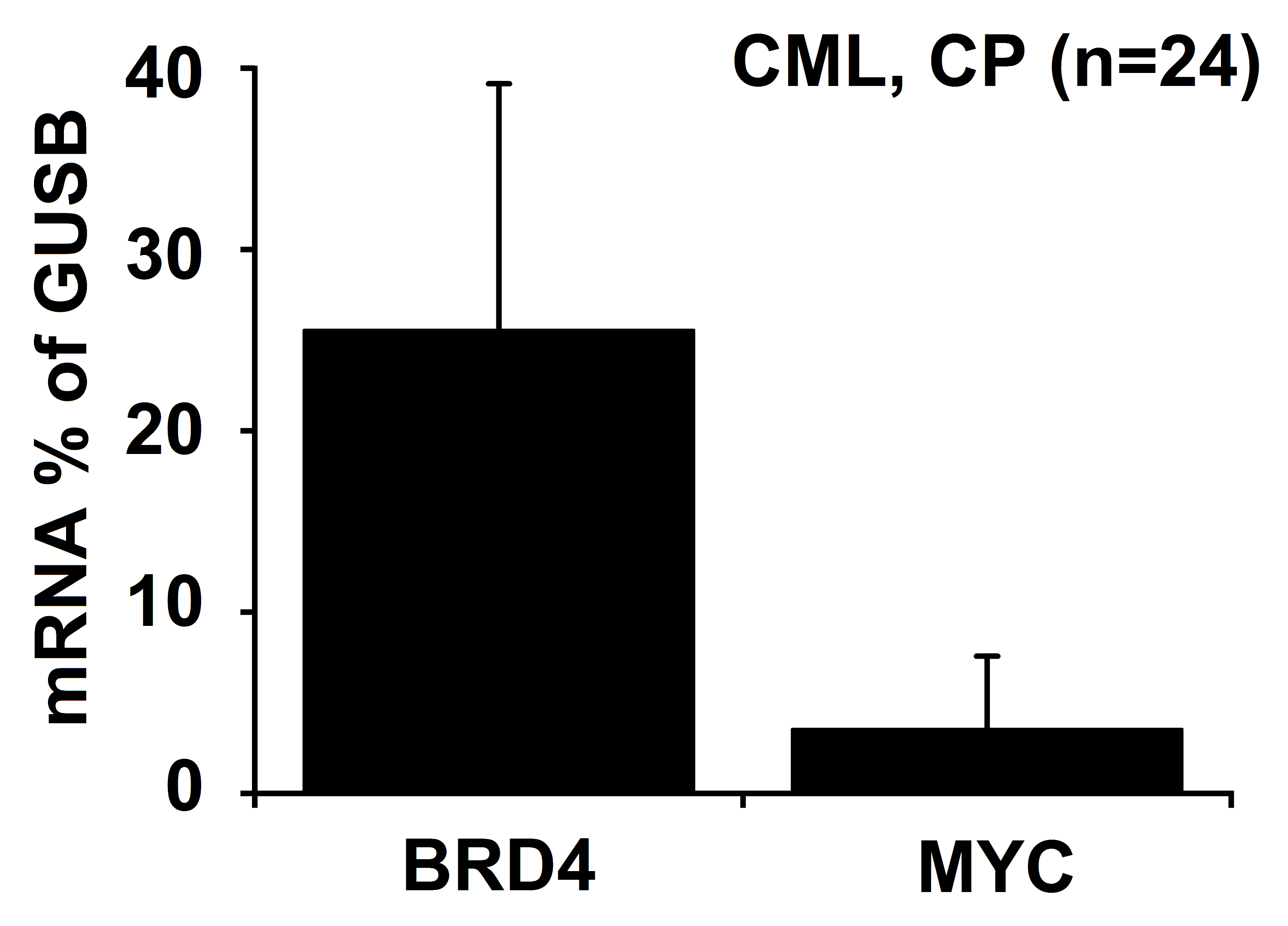


Peter et al., Supplemental Figure S1C

**Expression of BRD4 and MYC in CML cells**

A: KU812 cells (upper panel) and K562 cells (lower panel) were analyzed for the expression of BRD4 and MYC by immunocytochemistry as described in the text. A polyclonal antibody directed against BRD4 and a monoclonal antibody directed against MYC was used. Original magnification, x100. Original magnification, x60. Slides were investigated using an Olympus DP21 camera connected to an Olympus BX50F4 microscope equipped with 100x/1.35 UPlanAPO (Oil Iris) objective lenses. Images were adjusted by Adobe Photoshop CS5. B: KU812 cells and K562 cells were analyzed for the expression of BRD4 mRNA (left panel) and MYC mRNA (right panel) by qPCR as described in the text. Results are expressed as mRNA levels percent of GUSB mRNA levels and represent the mean±SD from 8 independent experiments. Asterisk: p<0.05. C: Primary CML MNC obtained from chronic phase (CP) CML patients (left panel) and accelerated phase (AP) or blast phase (BP) CML patients (right panel) were analyzed for the expression of MYC mRNA and BRD4 mRNA by qPCR as described in the text. Results are expressed in mRNA levels percent of GUSB mRNA levels and represent the mean±SD from 24 CML CP samples and the mean±SD from 15 CML AP/BP samples (AP, n=2; BP, n=13).

Supplemental Figure S2


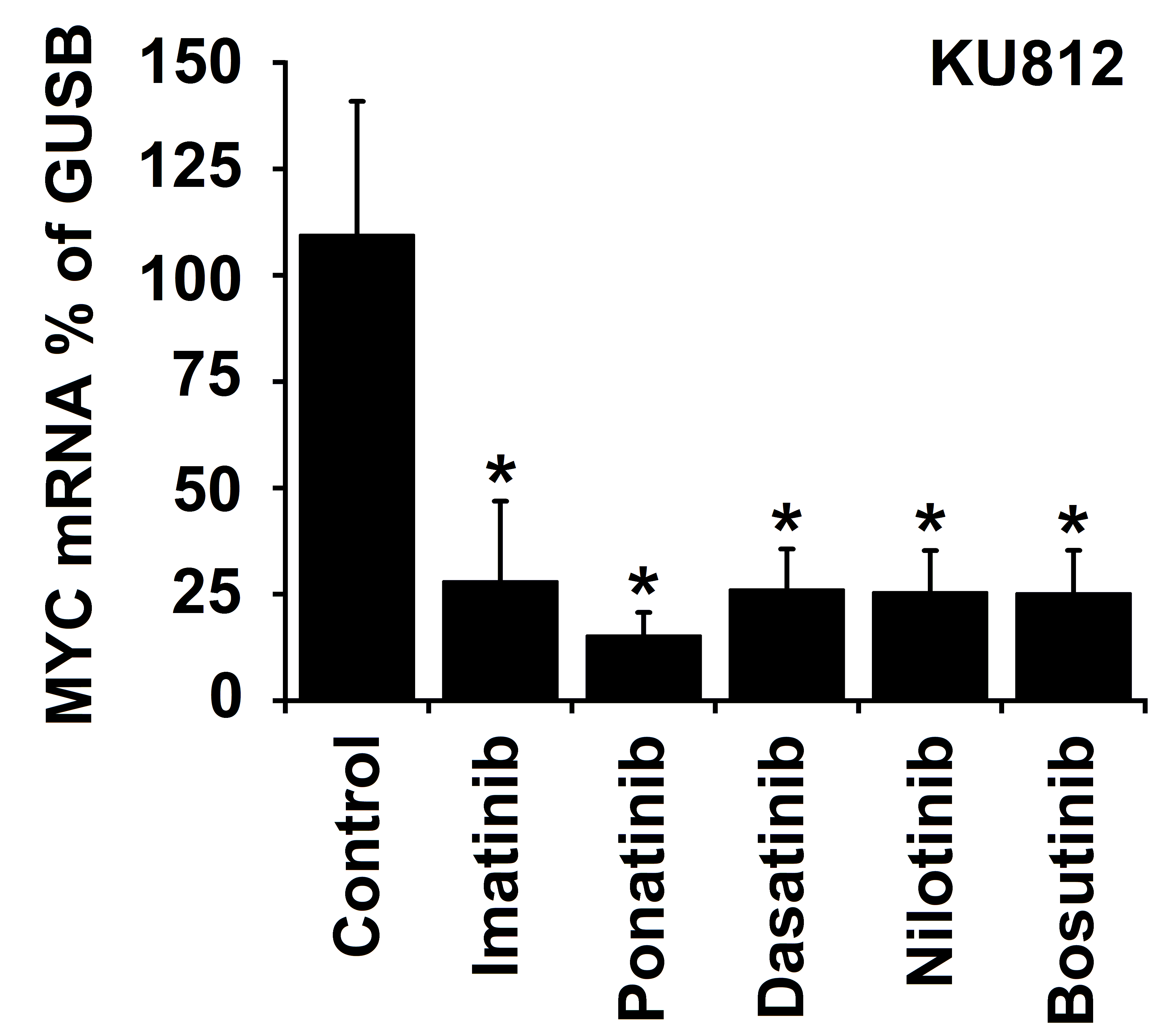

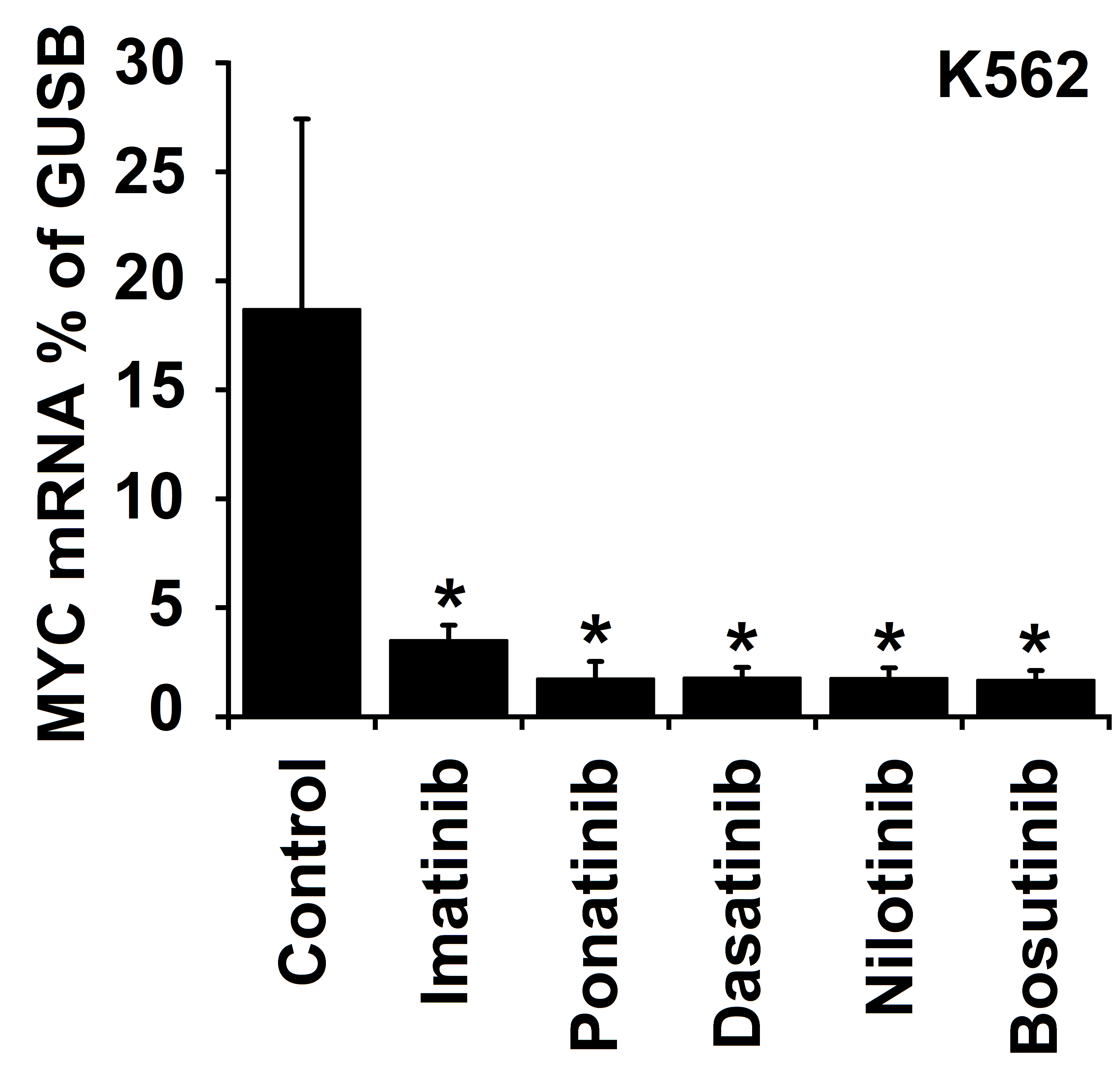

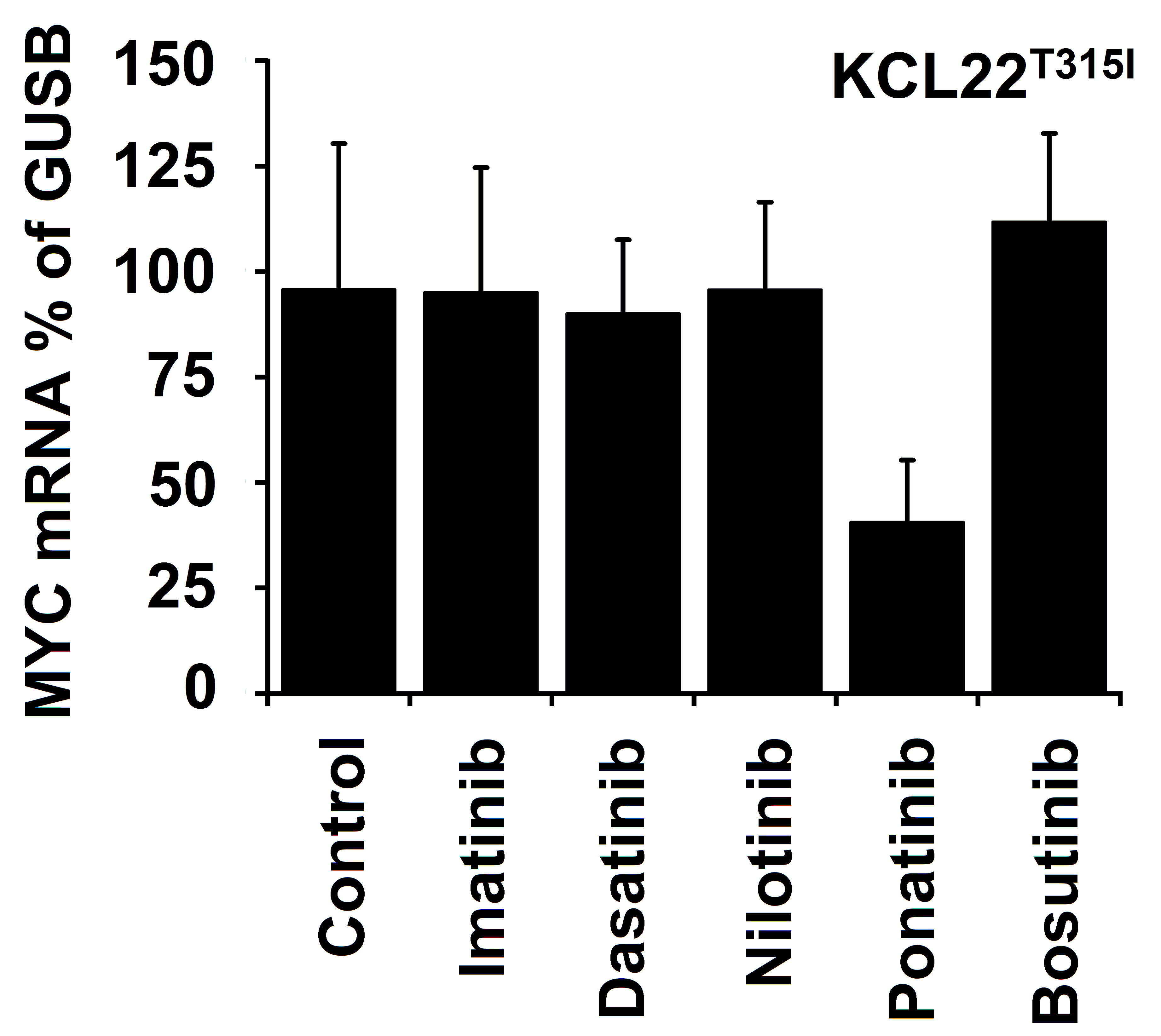


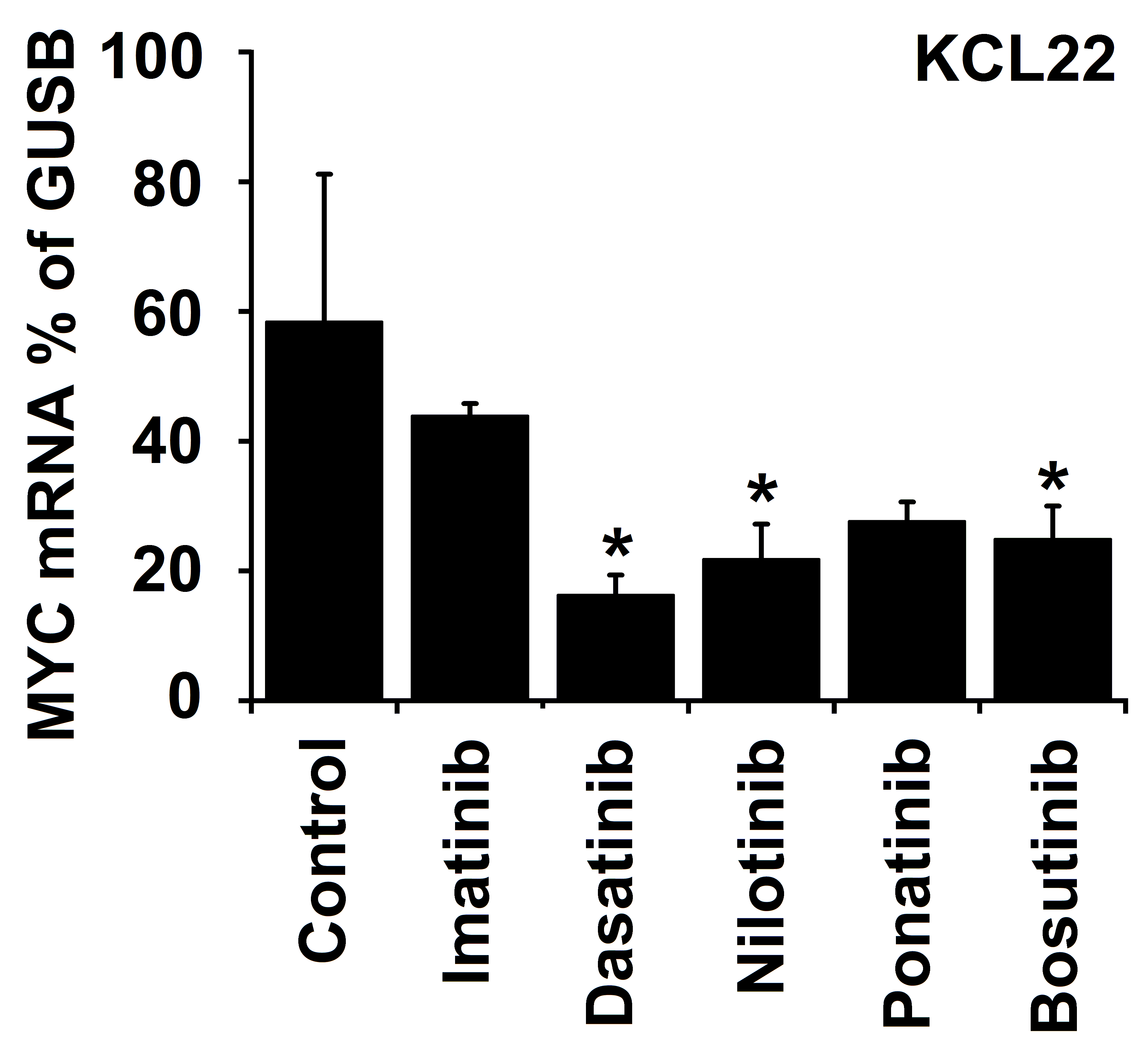


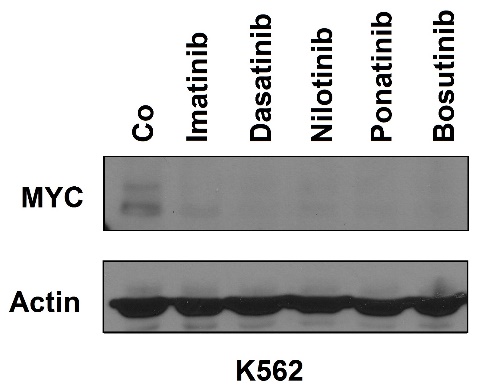
Peter et al., Supplemental Figure S2A


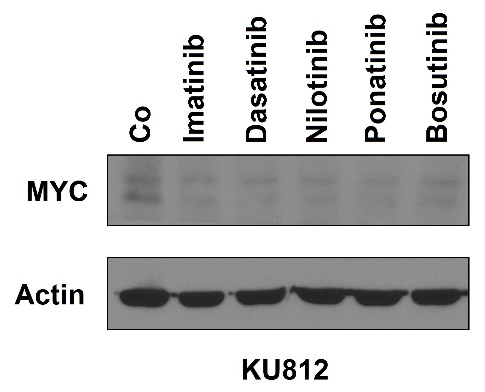


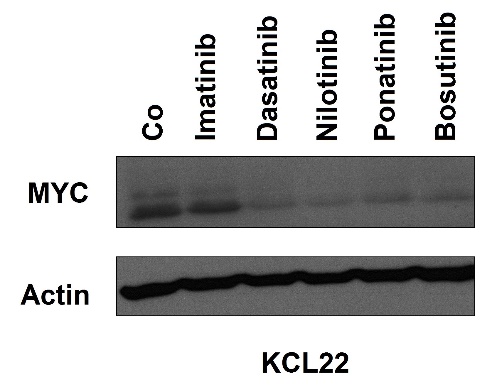

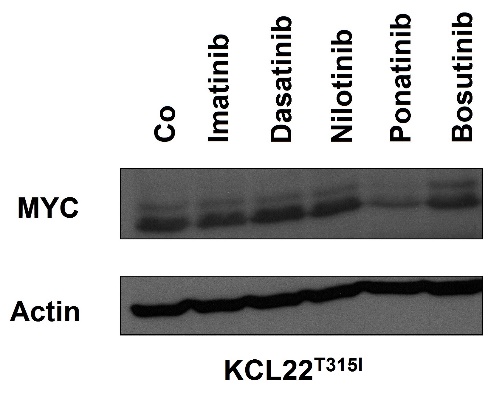


Peter et al., Supplemental Figure S2B


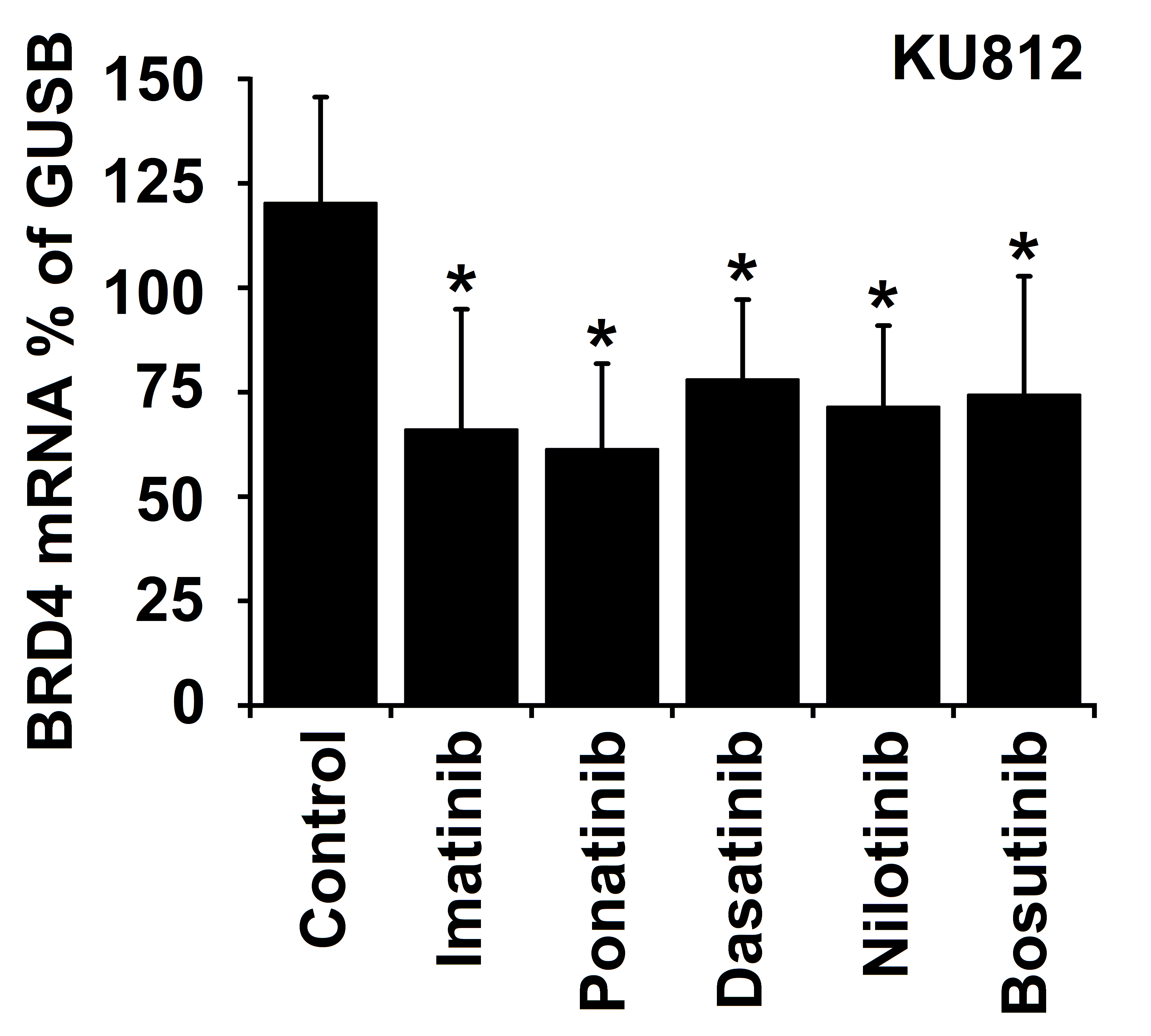

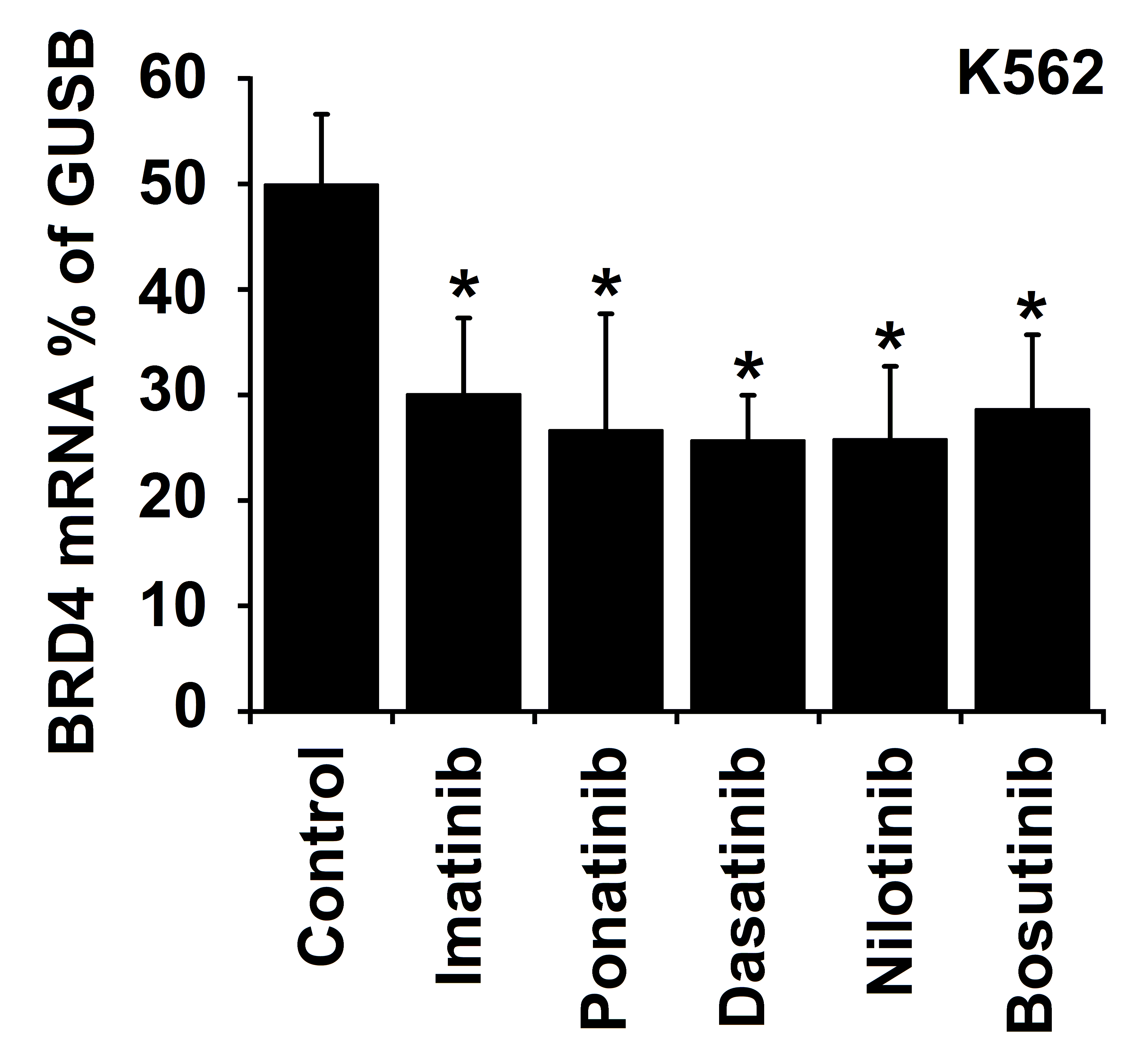


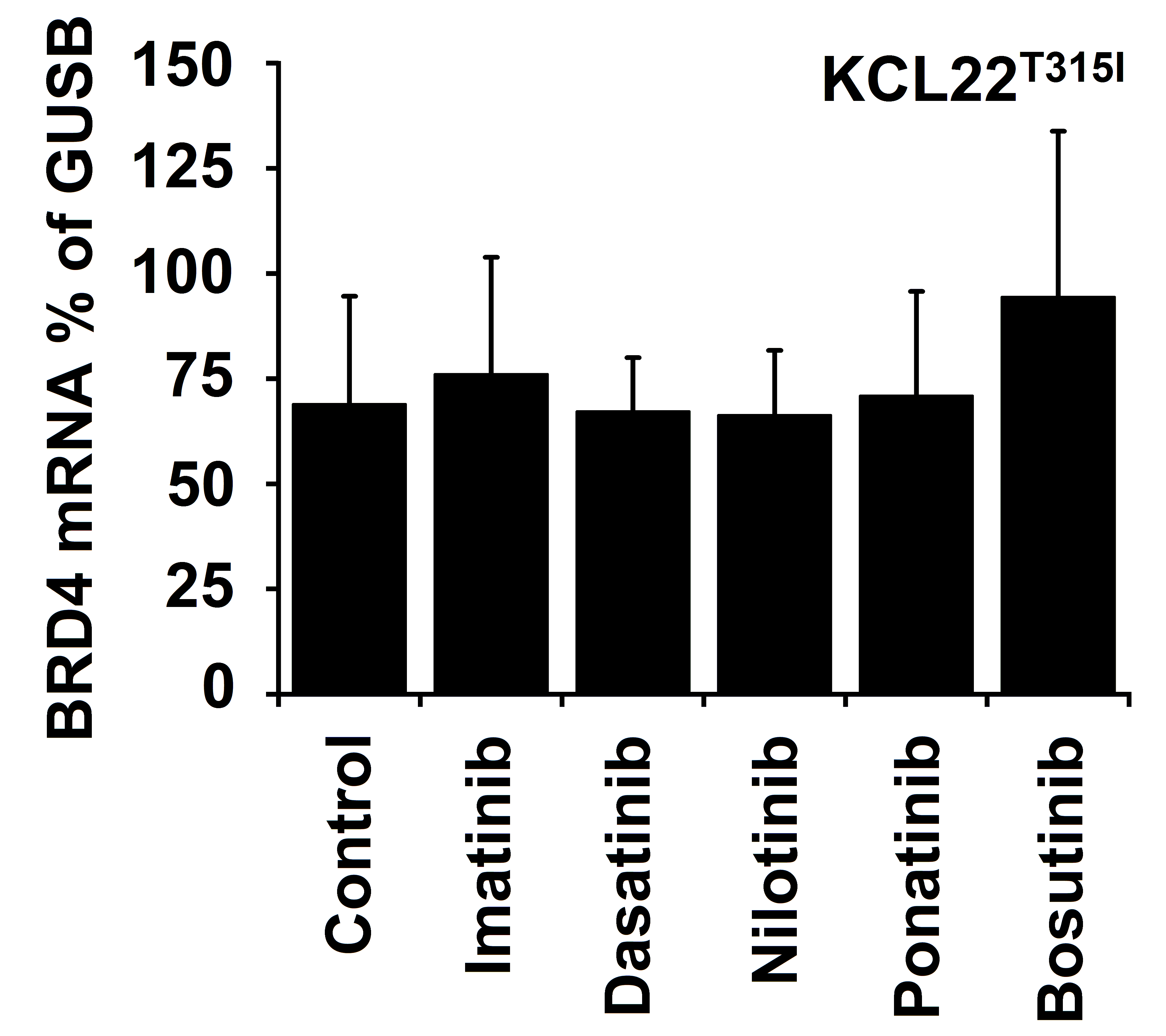

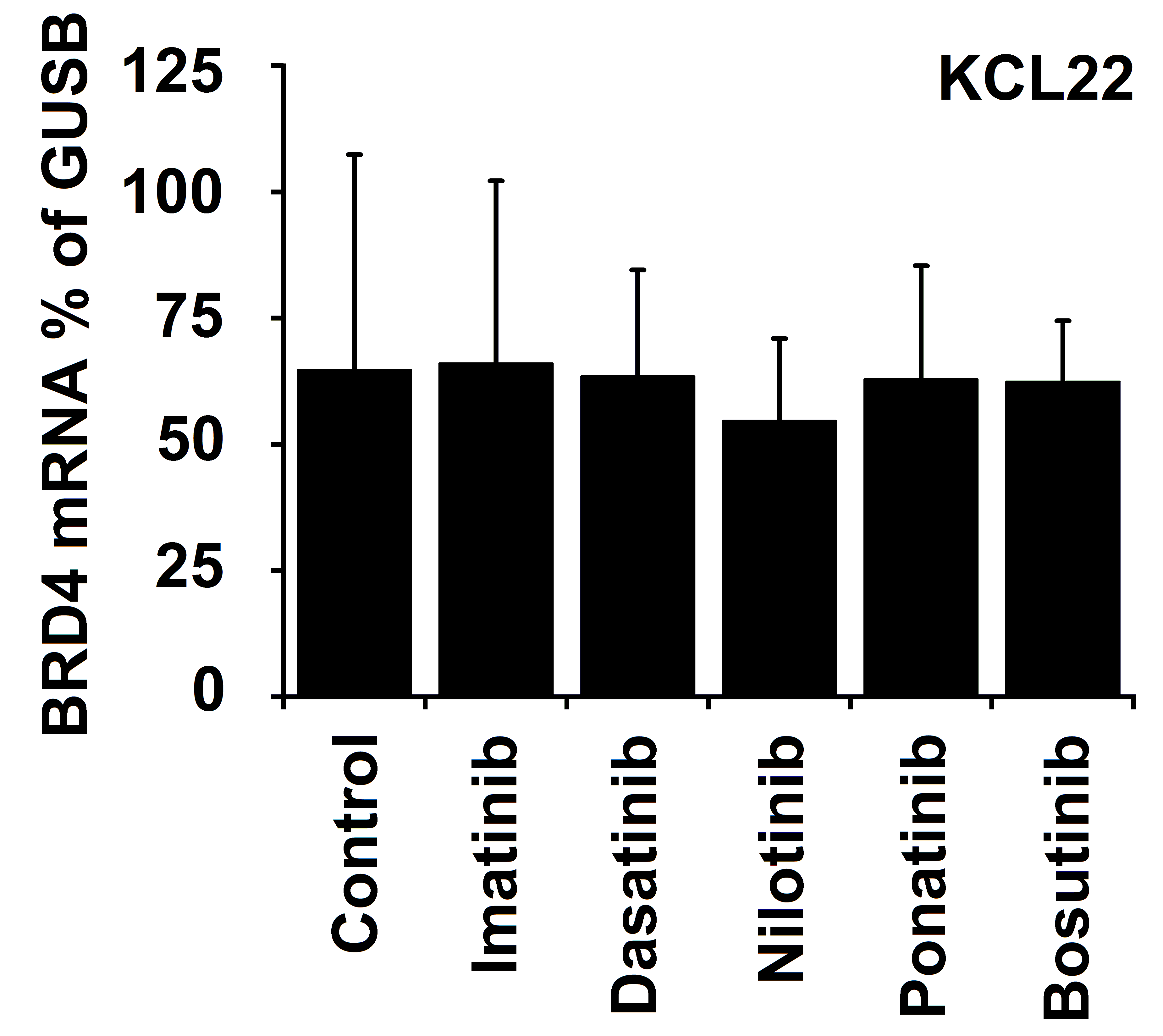


Peter et al., Supplemental Figure S2C

**
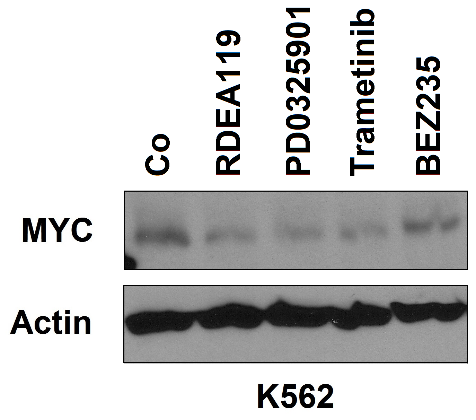

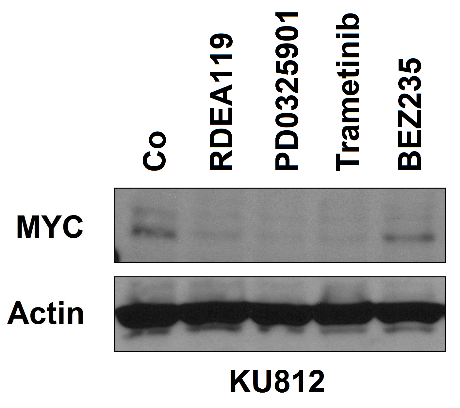
**

Peter et al., Supplemental Figure S2D

**Effects of BCR::ABL1 TKI on MYC expression**

A,C: KU812, K562, KCL22 and KCL22^T315I^ cells were incubated in medium (control) or medium containing imatinib, dasatinib, dasatinib, bosutinib, ponatinib (1 µM each) at 37°C for 24 hours. Thereafter cells were harvested and qPCR was performed as described. Results are expressed as MYC mRNA (A) or BRD4 mRNA (C) levels percent of GUSB mRNA levels and represent the mean±SD from 4 independent experiments. Asterisk: p<0.05 compared to control. B: KU812 and K562 cells (upper panel), and KCL22 and KCL22^T315I^ cells (lower panel) were incubated in medium (Co) or medium containing imatinib, dasatinib, dasatinib, bosutinib, ponatinib (1 µM each) at 37°C for 24 hours. Thereafter, cells were harvested and Western blotting was performed using antibodies directed against MYC and Actin. D: KU812 and K562 cells were incubated in medium (Co) or medium containing RDEA119 (refametinib), PD0325901, trametinib or BEZ235 (each 1 µM) at 37°C for 24 hours. Thereafter, cells were harvested and Western blotting was performed using antibodies against MYC and Actin.


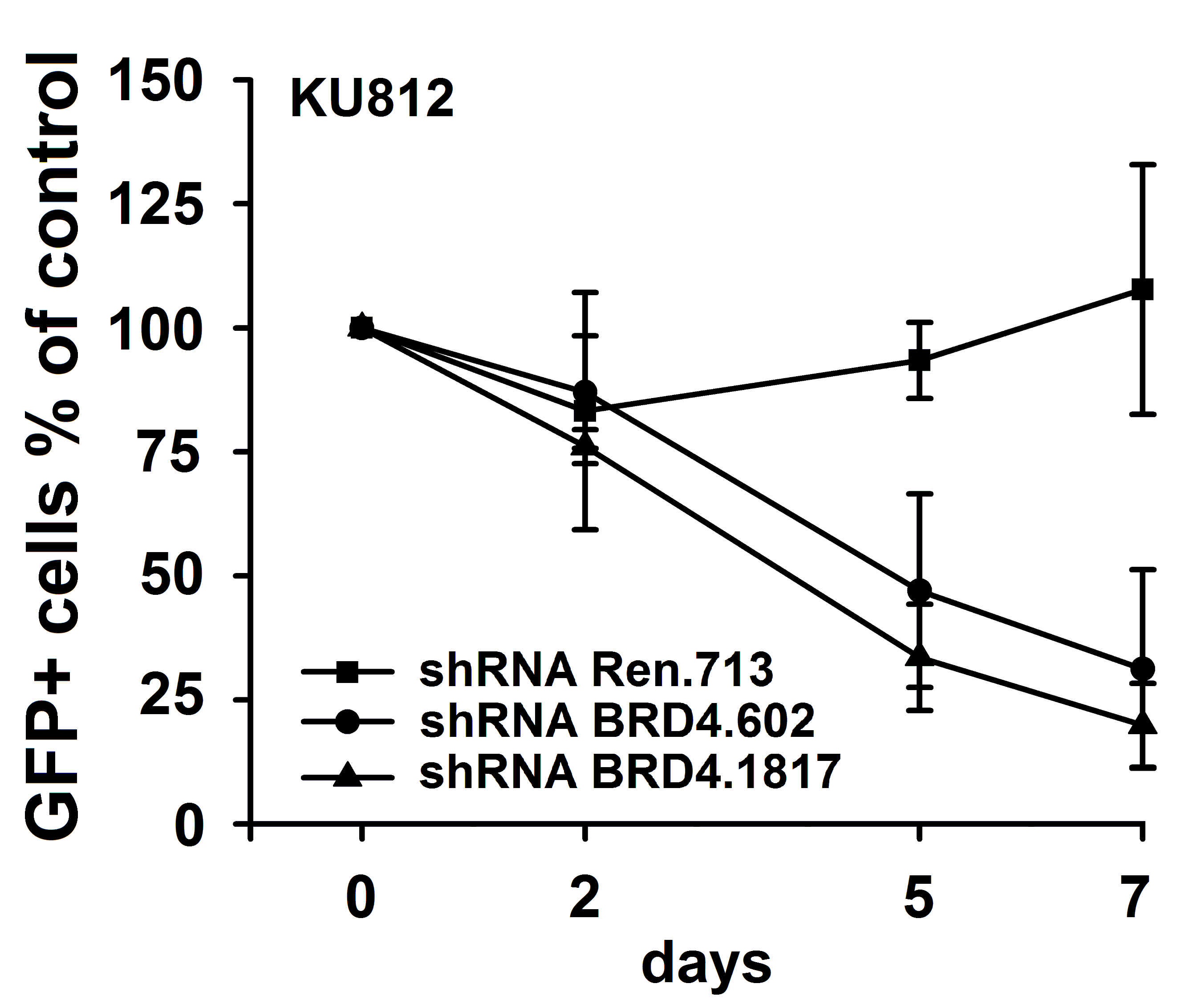

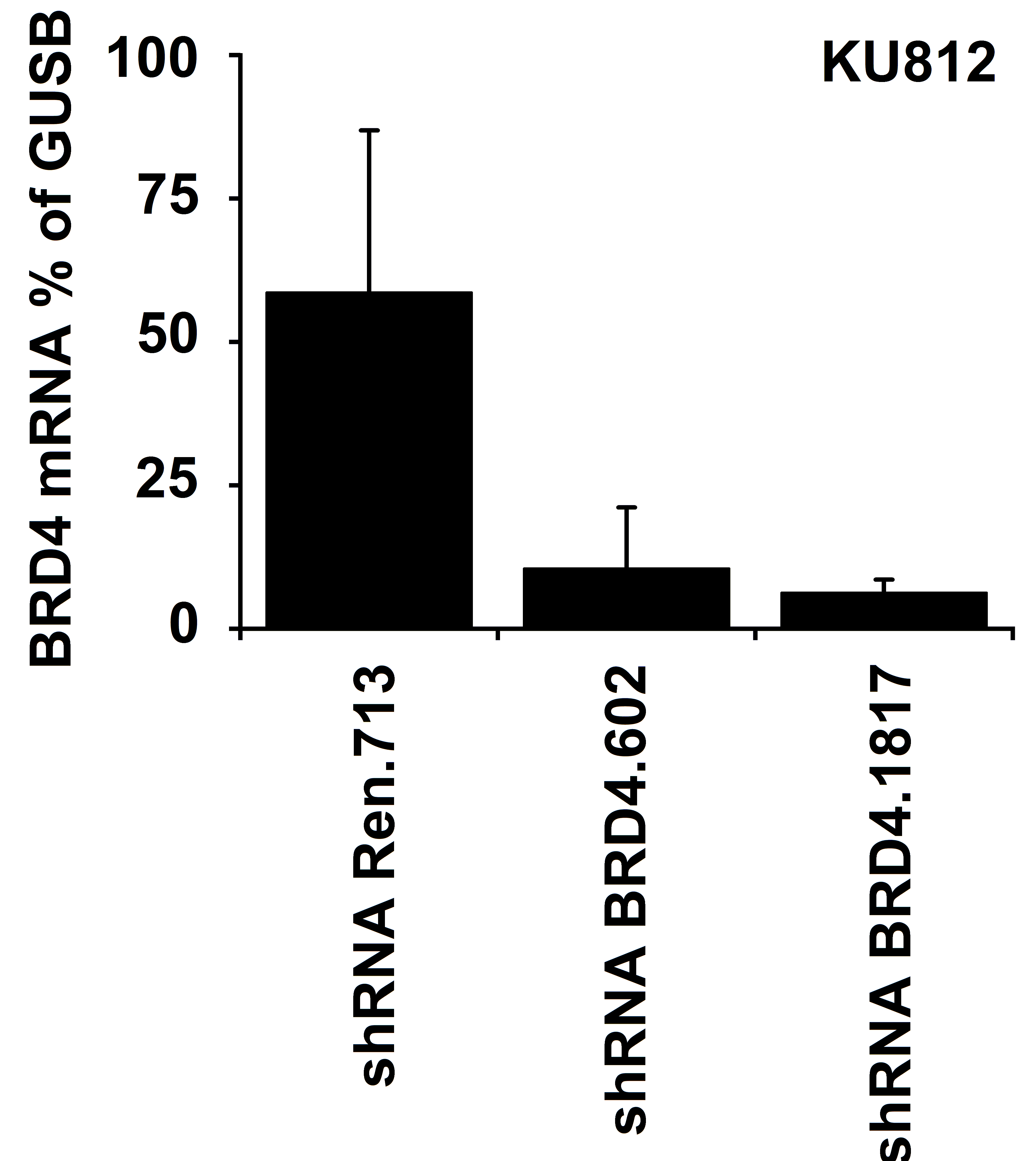
Supplemental Figure S3


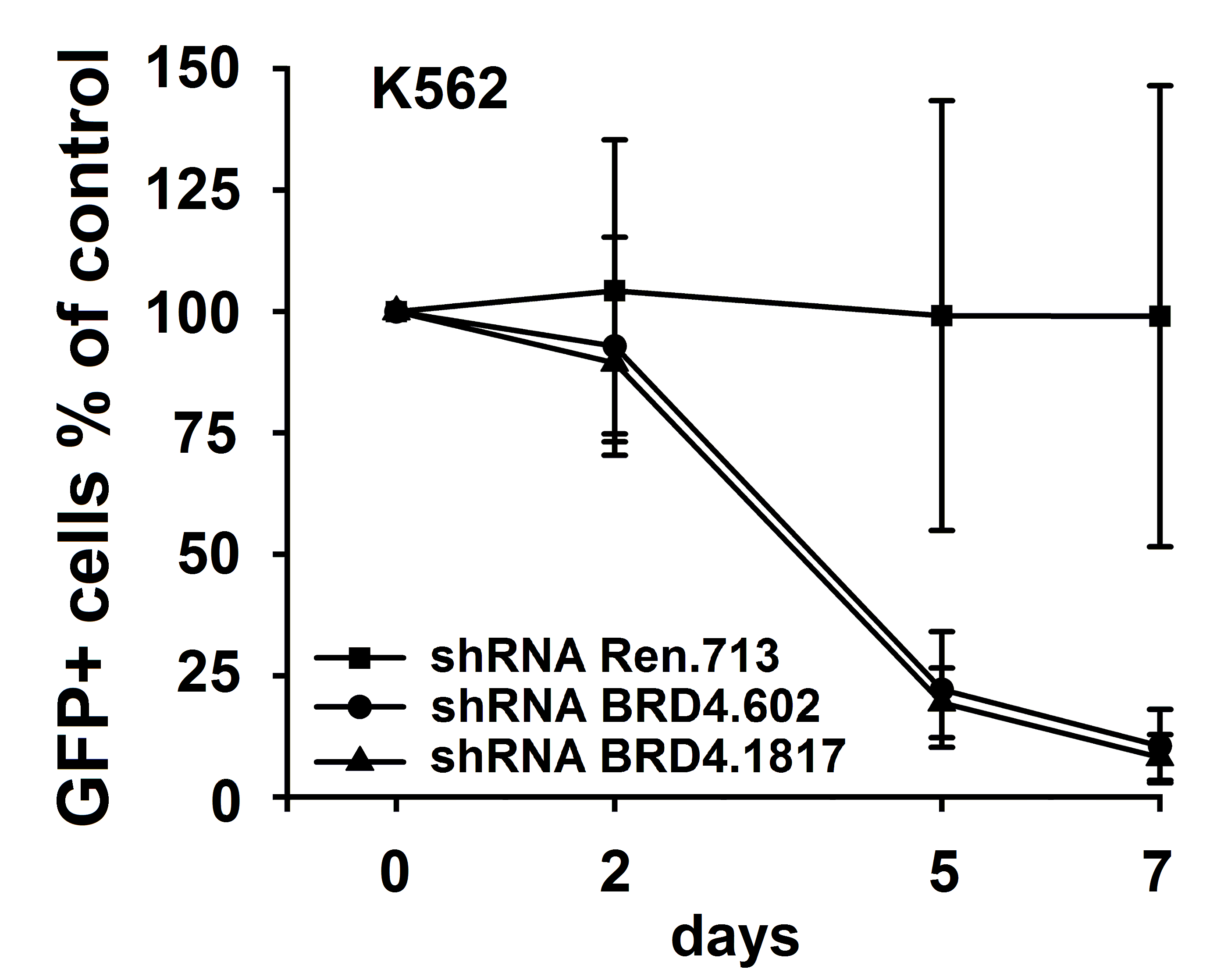

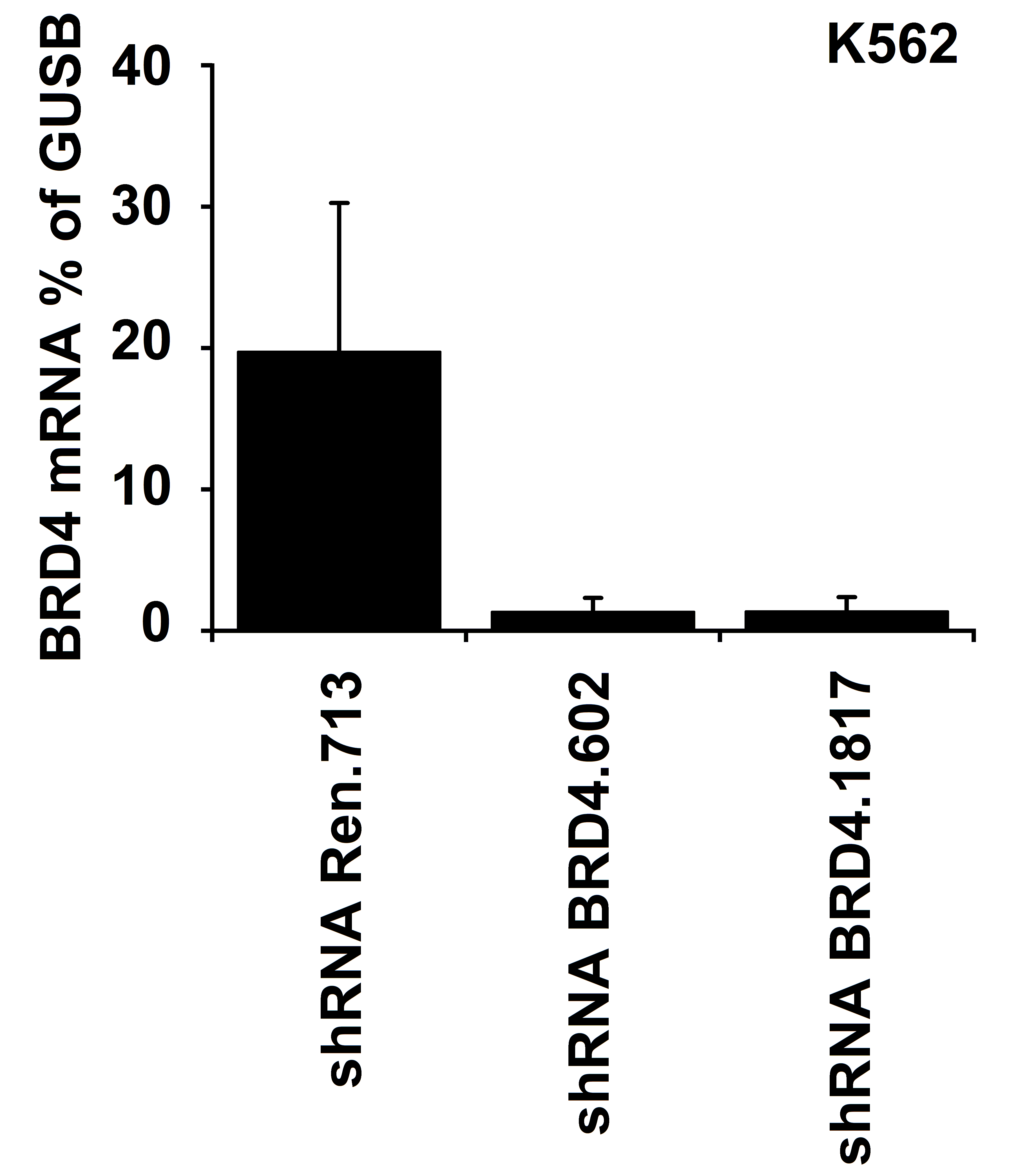


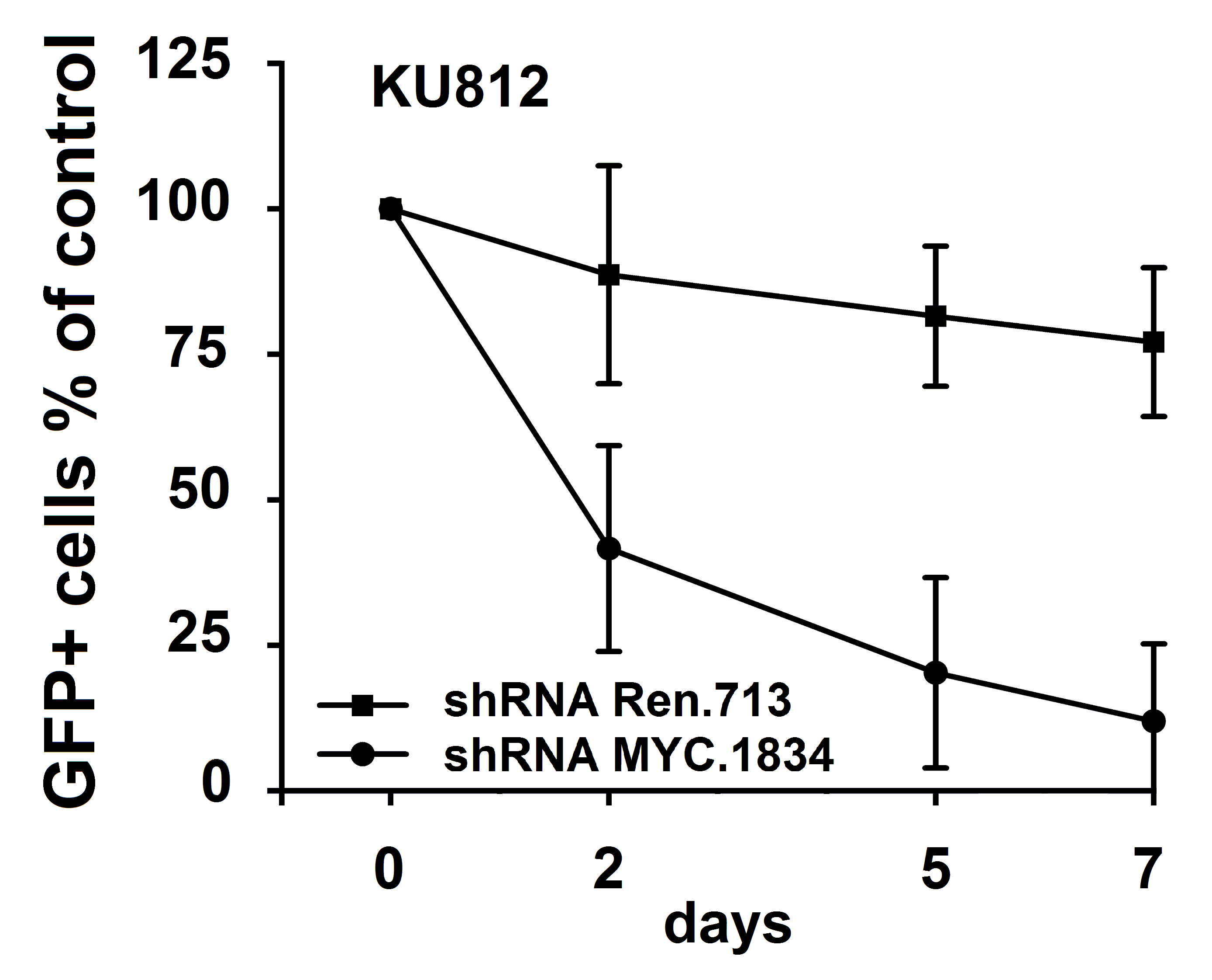
 Peter et al., Figure S3A


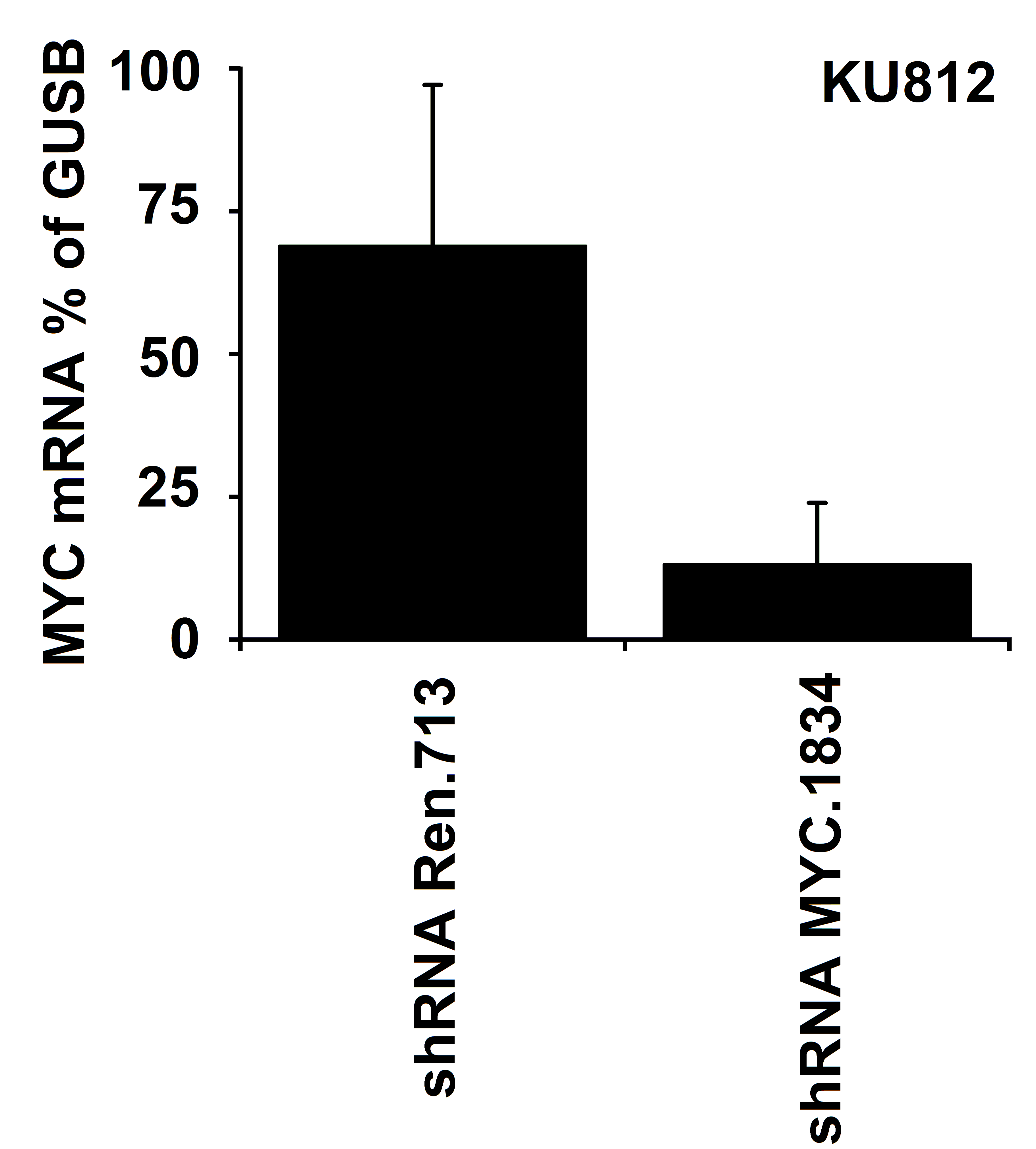


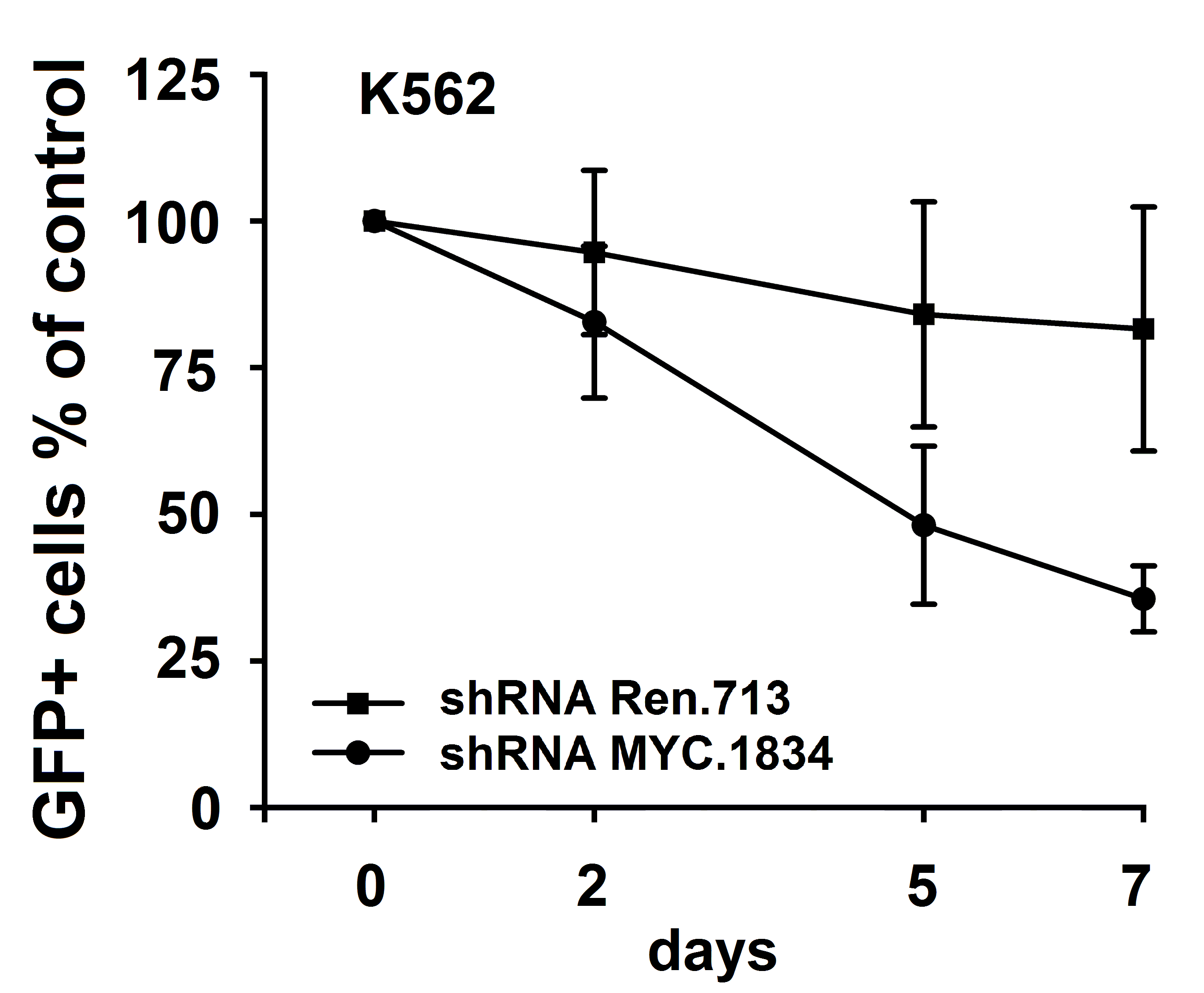


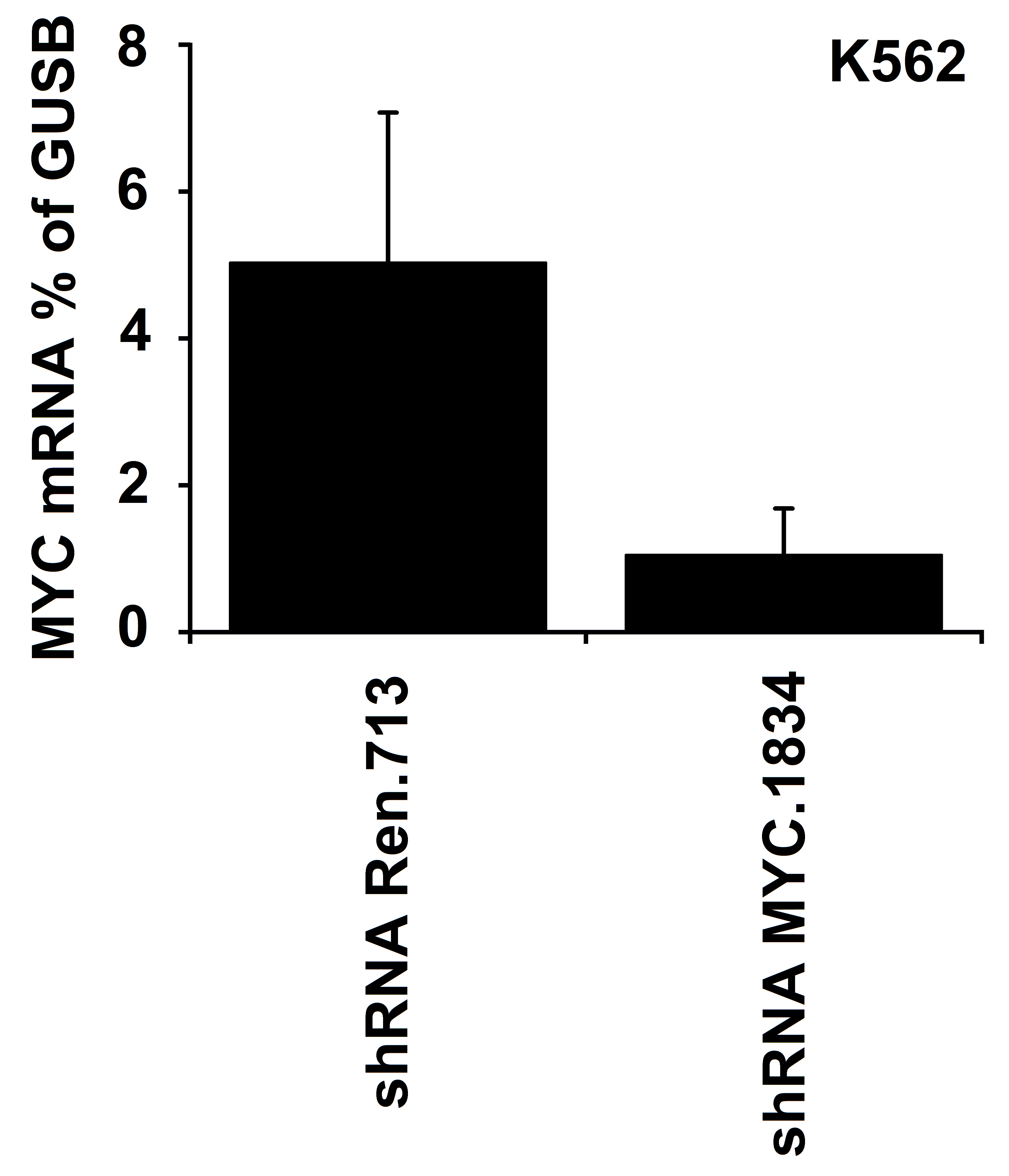


Peter et al., Figure S3B

**Effects of shRNA-induced knockdown of BRD4 or MYC in KU812 and K562 cells**

A,B: KU812 cells (upper panels) and K562 cells (lower panels) were transduced lentivirally with shRNA against BRD4 (A) or MYC (B) or renilla luciferase (Ren) (A,B) as described in the text. shRNA-transduced cells were mixed 1:1 with untransduced cells. The percentages of GFP-positive cells in these cultures were monitored for 7 days by flow cytometry. Results are shown as GFP+ cells (%) as percent of control (day 0) and represent the mean±S.D. from 3 independent experiments. The knockdown of BRD4 and MYC was confirmed by qPCR. Results are expressed as BRD4 mRNA (A) or MYC mRNA (B) levels percent of GUSB mRNA levels and represent the mean±SD from 3 independent experiments.

Supplemental Figure S4


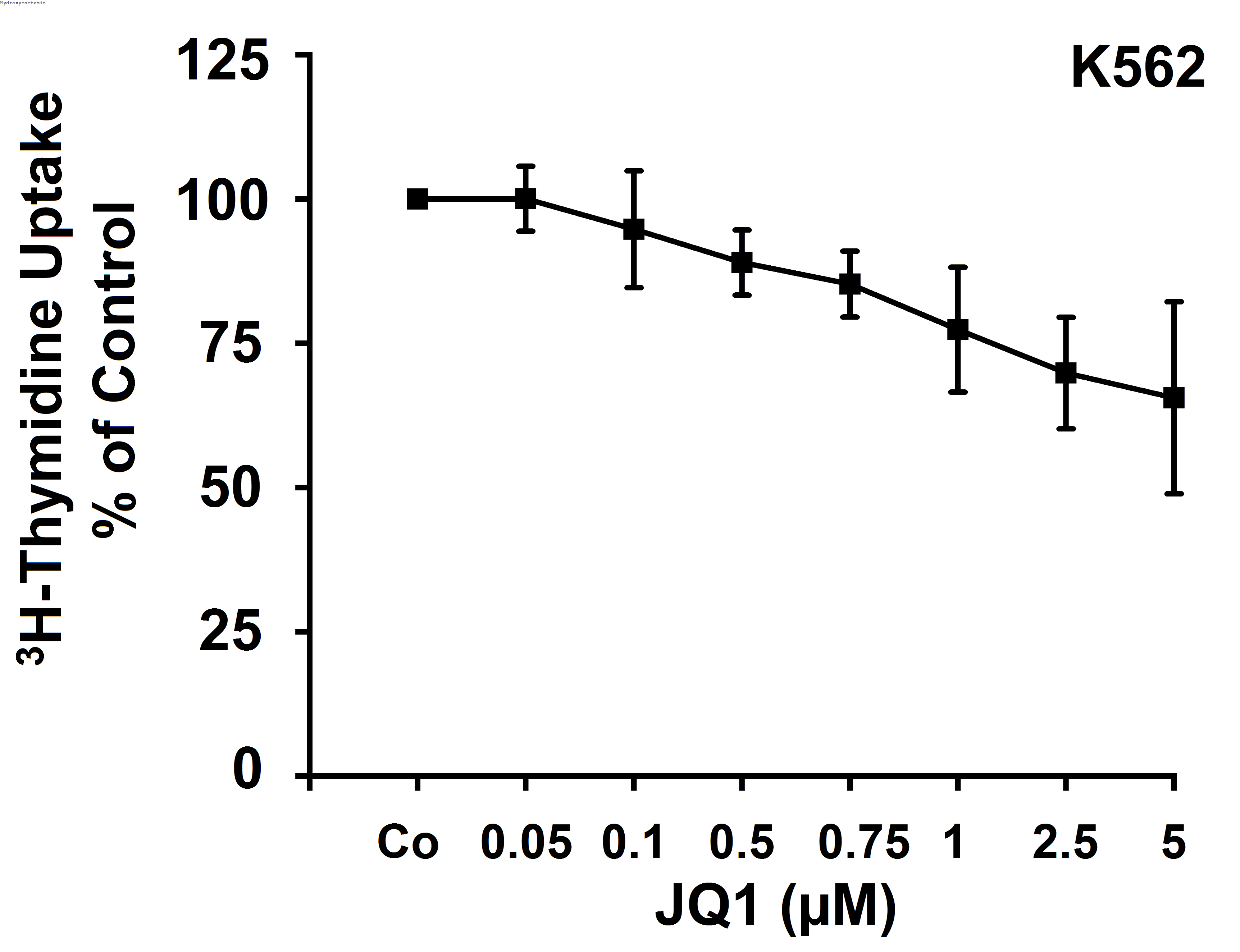

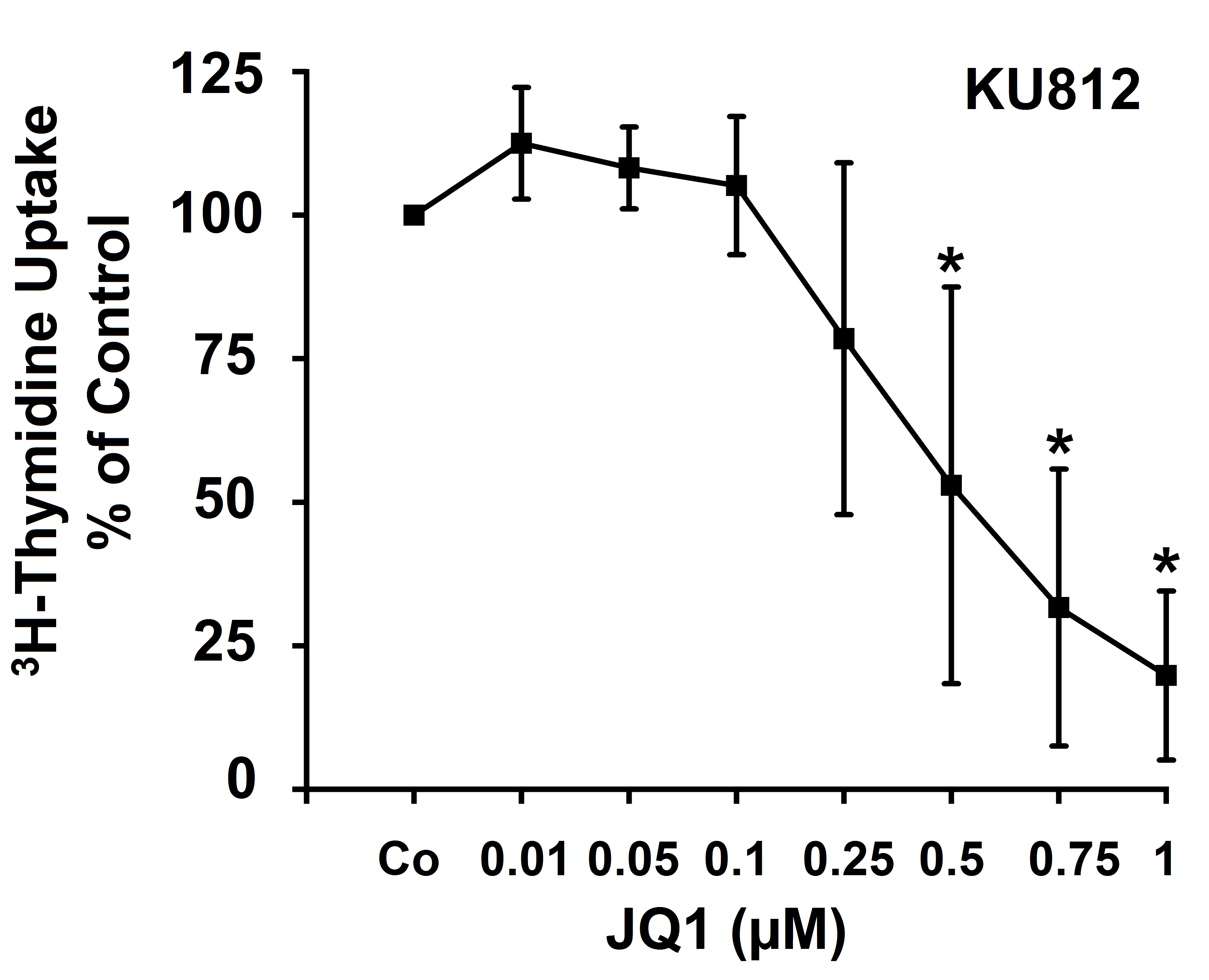


Peter et al., Supplemental Figure S4A


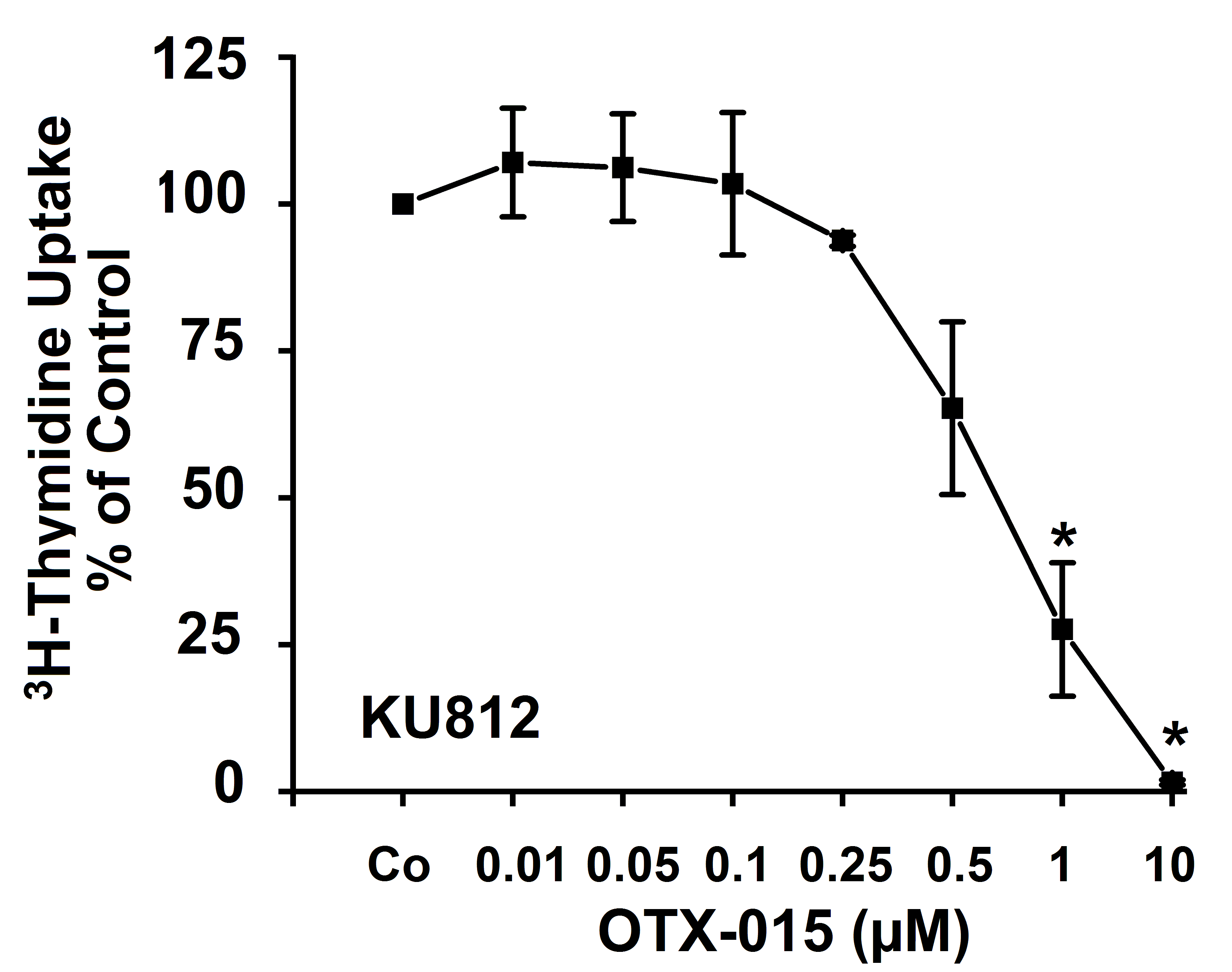

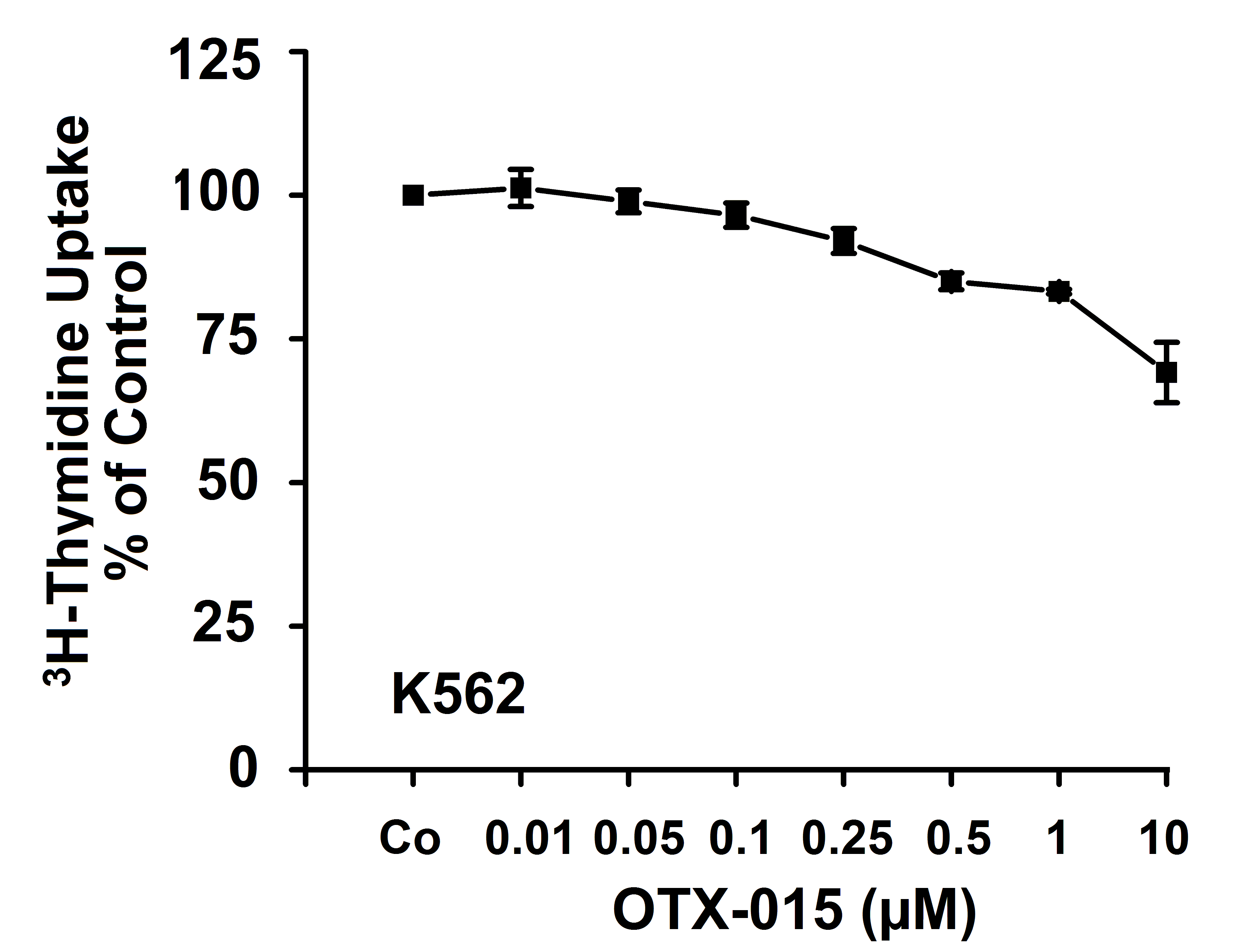


Peter et al., Supplemental Figure S4B


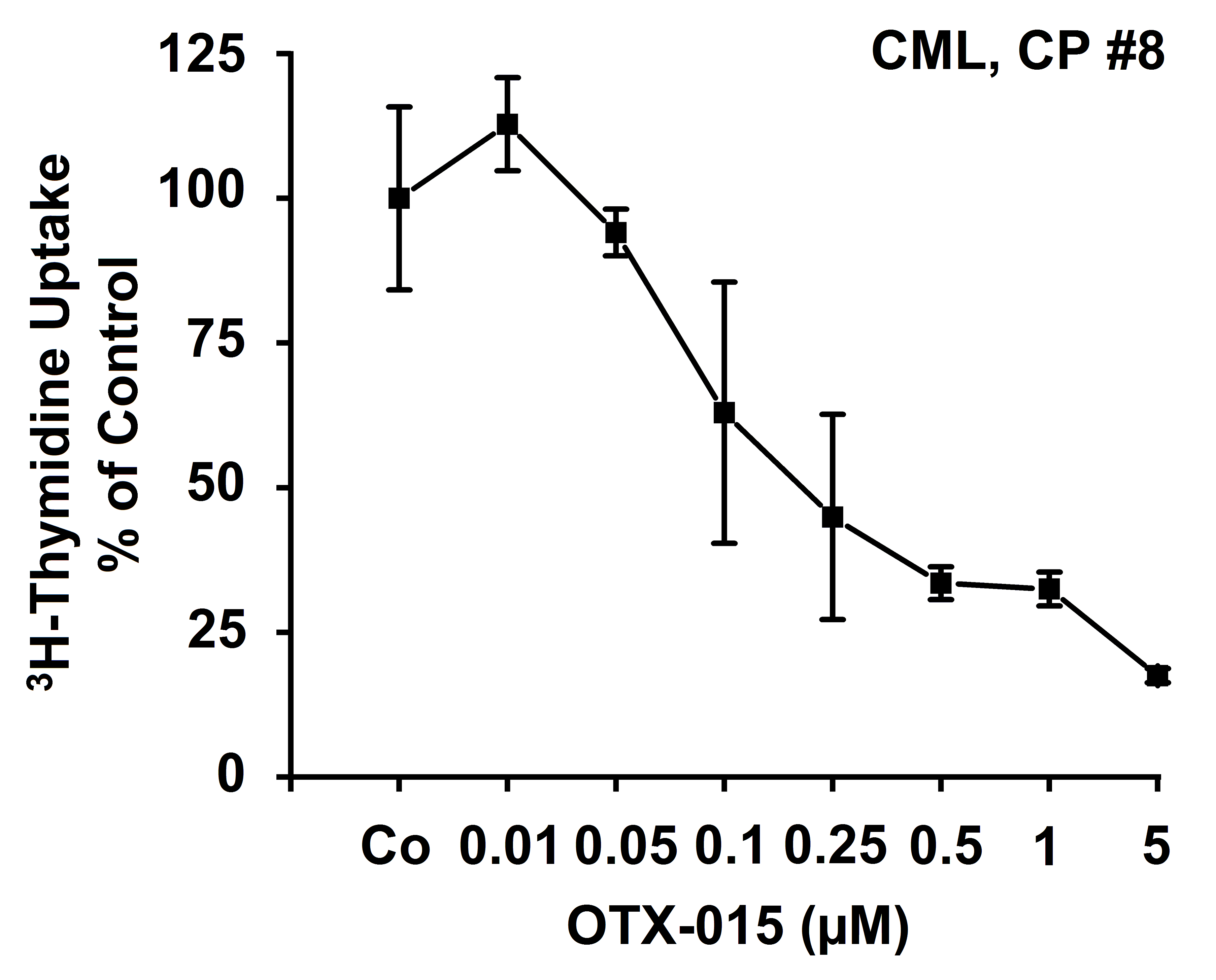

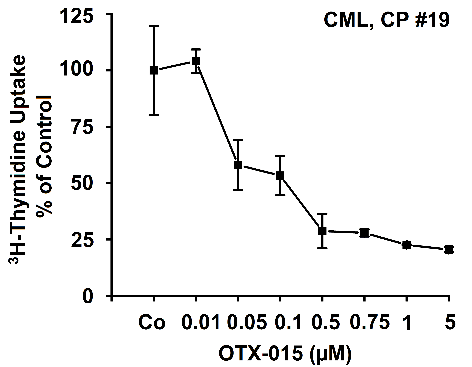


Peter et al., Supplemental Figure S4C

**Effects of BET-blockers on proliferation of CML cells**

A: KU812 cells (left panel) and K562 cells (right panel) were incubated in medium (Co) or in various concentrations of JQ1 as indicated at 37°C for 48 hours. B: KU812 cells (left panel) and K562 cells (right panel) were incubated with medium (Co) or various concentrations of OTX-015 as indicated at 37°C for 48 hours.
C: MNC obtained from patients with CML CP were incubated with medium (Co) or various concentrations of OTX-015 at 37°C for 48 hours. Results from “A and B” are expressed as percent of control and represent the mean±SD from at least 3 independent experiments. Asterisk: p<0.05 compared to control. Results from “C” are expressed as percent of control and represent the mean±SD from triplicates.

Supplemental Figure S5


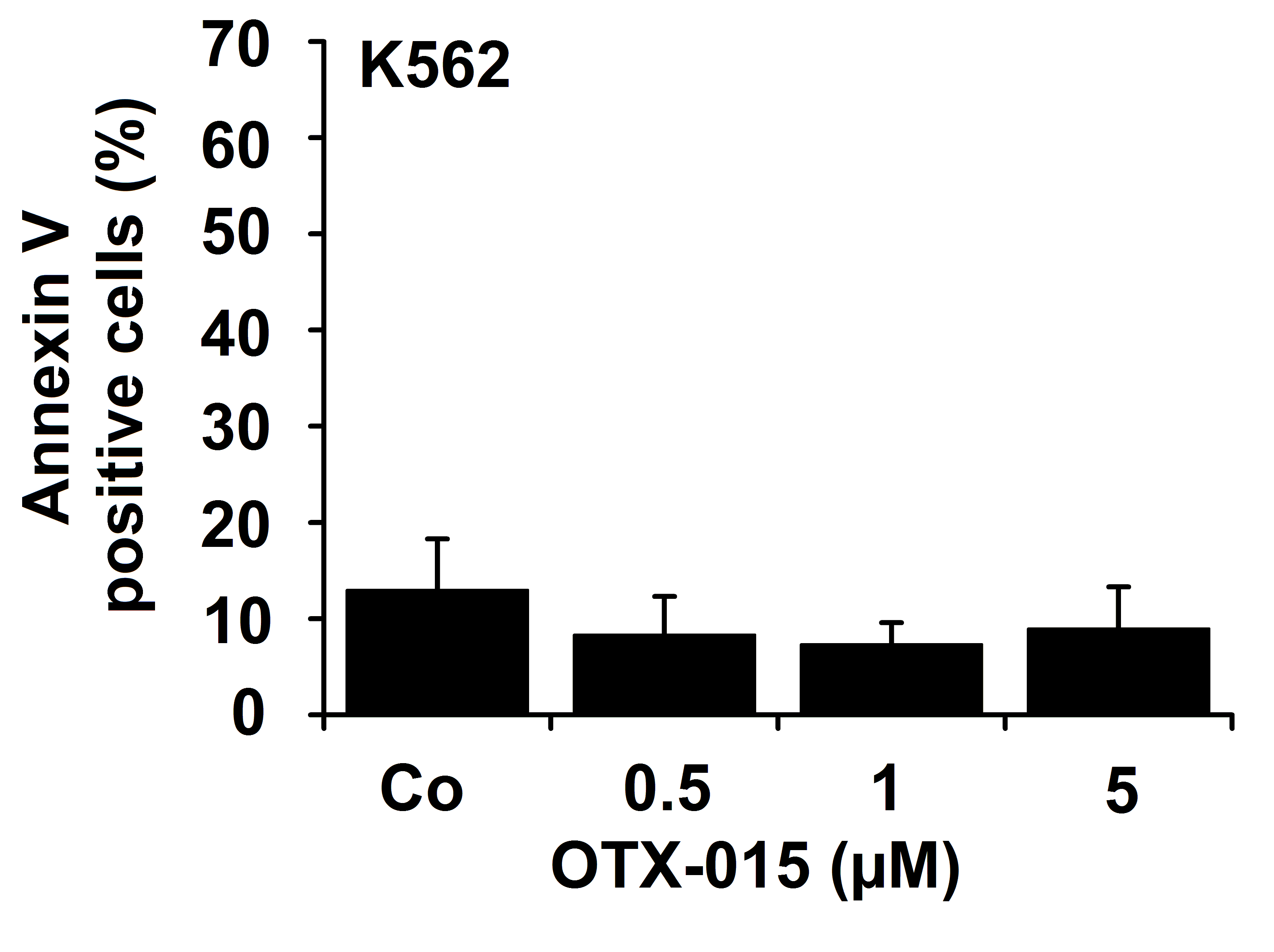

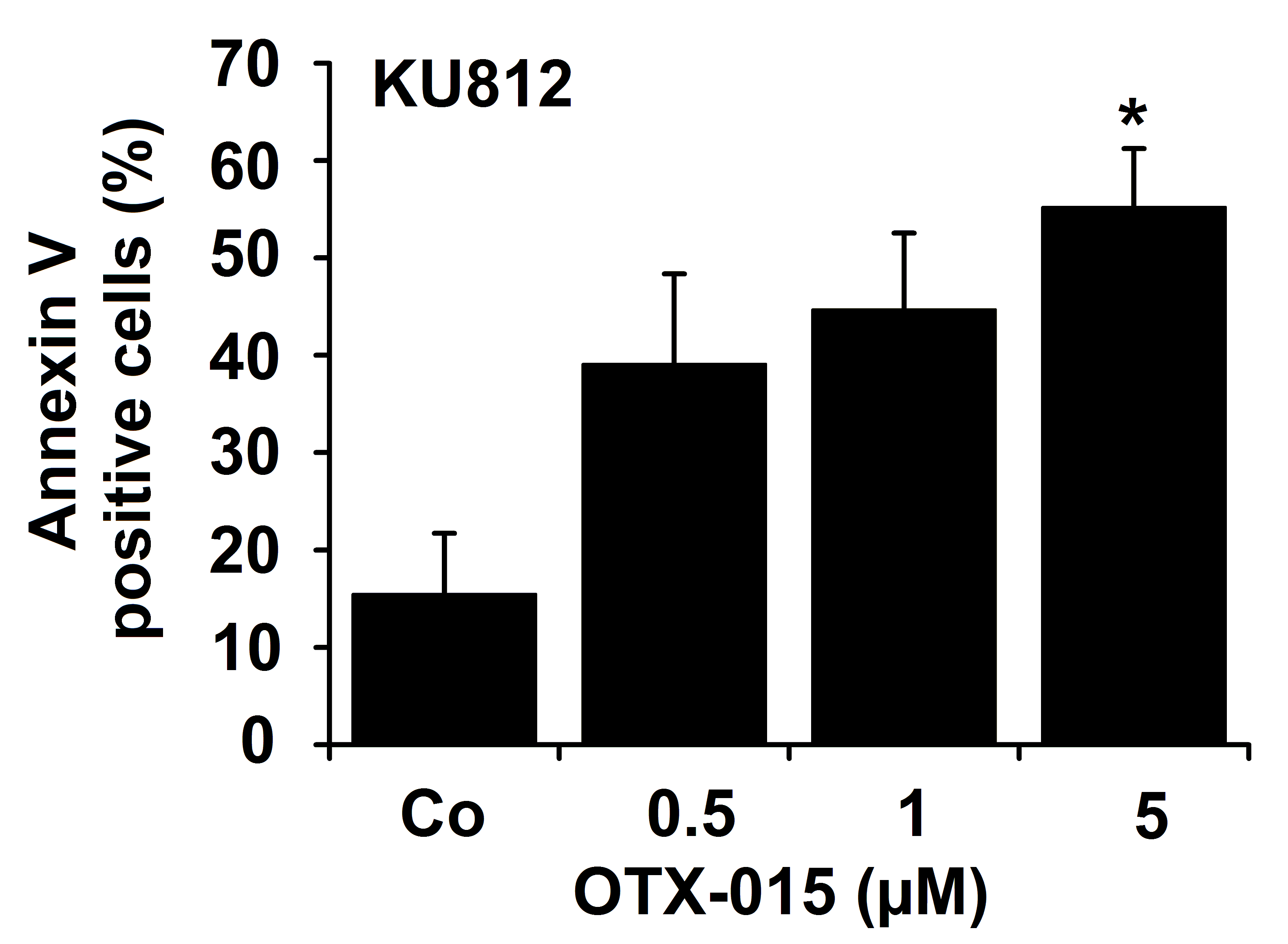


Peter et al. Supplemental Figure S5

**Effects of OTX-015 on survival of KU812 cells and K562 cells**

KU812 cells and K562 cells were incubated in medium (Co) with or without various concentrations of OTX-015 as indicated at 37°C for 48 hours. Thereafter cells were harvested and percentages of Annexin V positive cells were analyzed by flow cytometry. Results are expressed as Annexin V positive cells (%) and represent the mean±SD from 3 independent experiments. Asterisk: p<0.05 compared to control.

Supplemental Figure S6


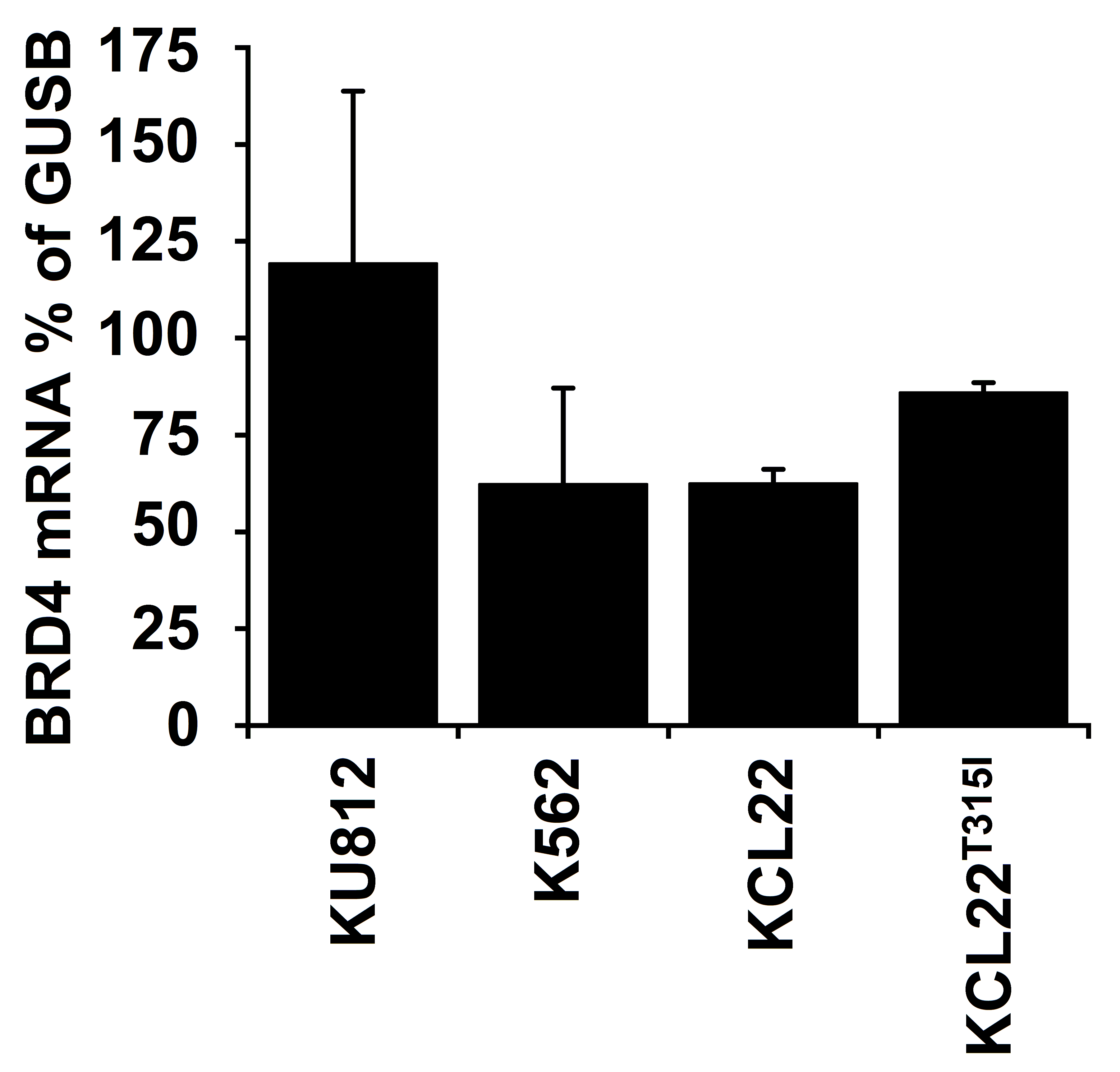

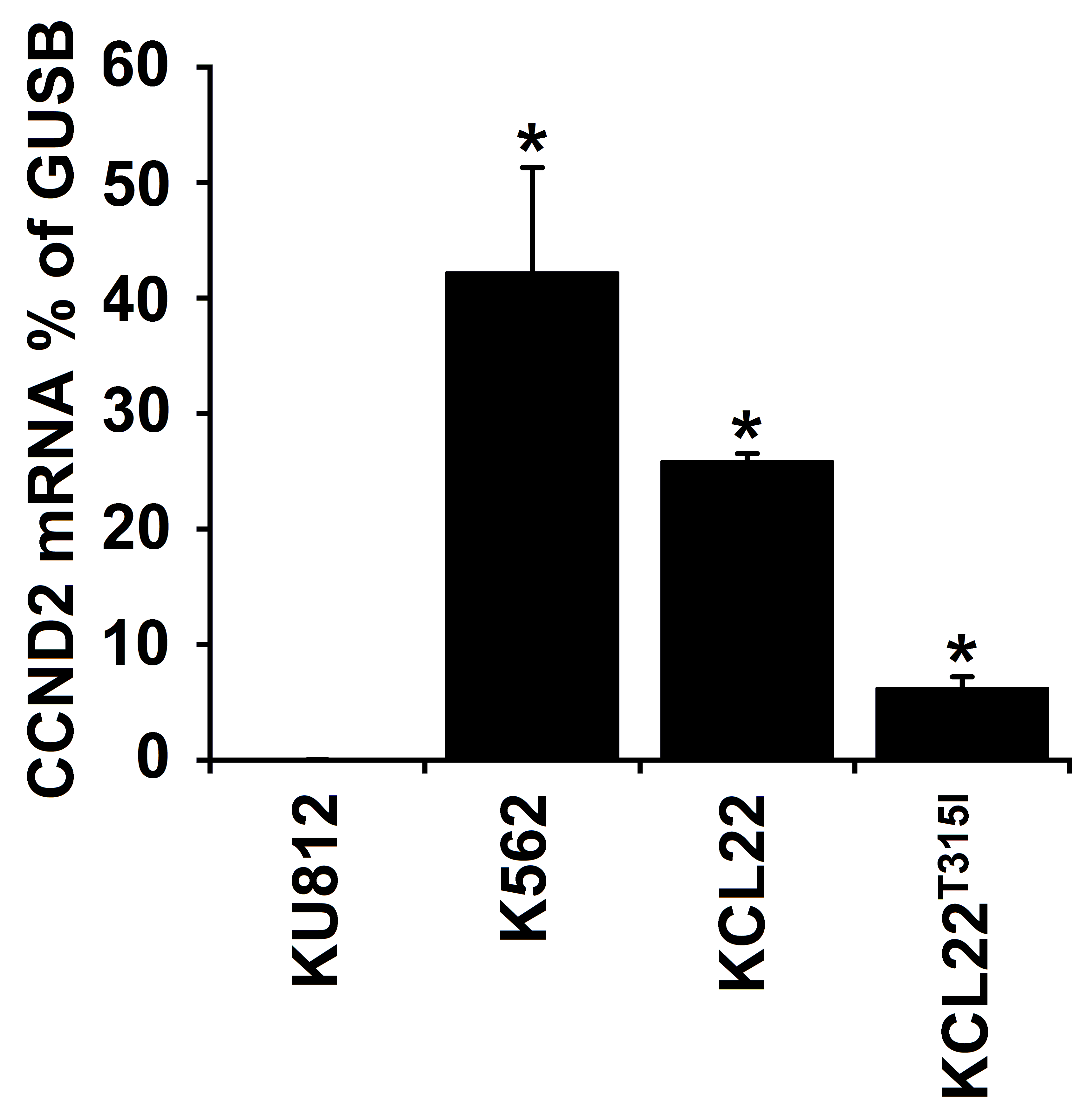

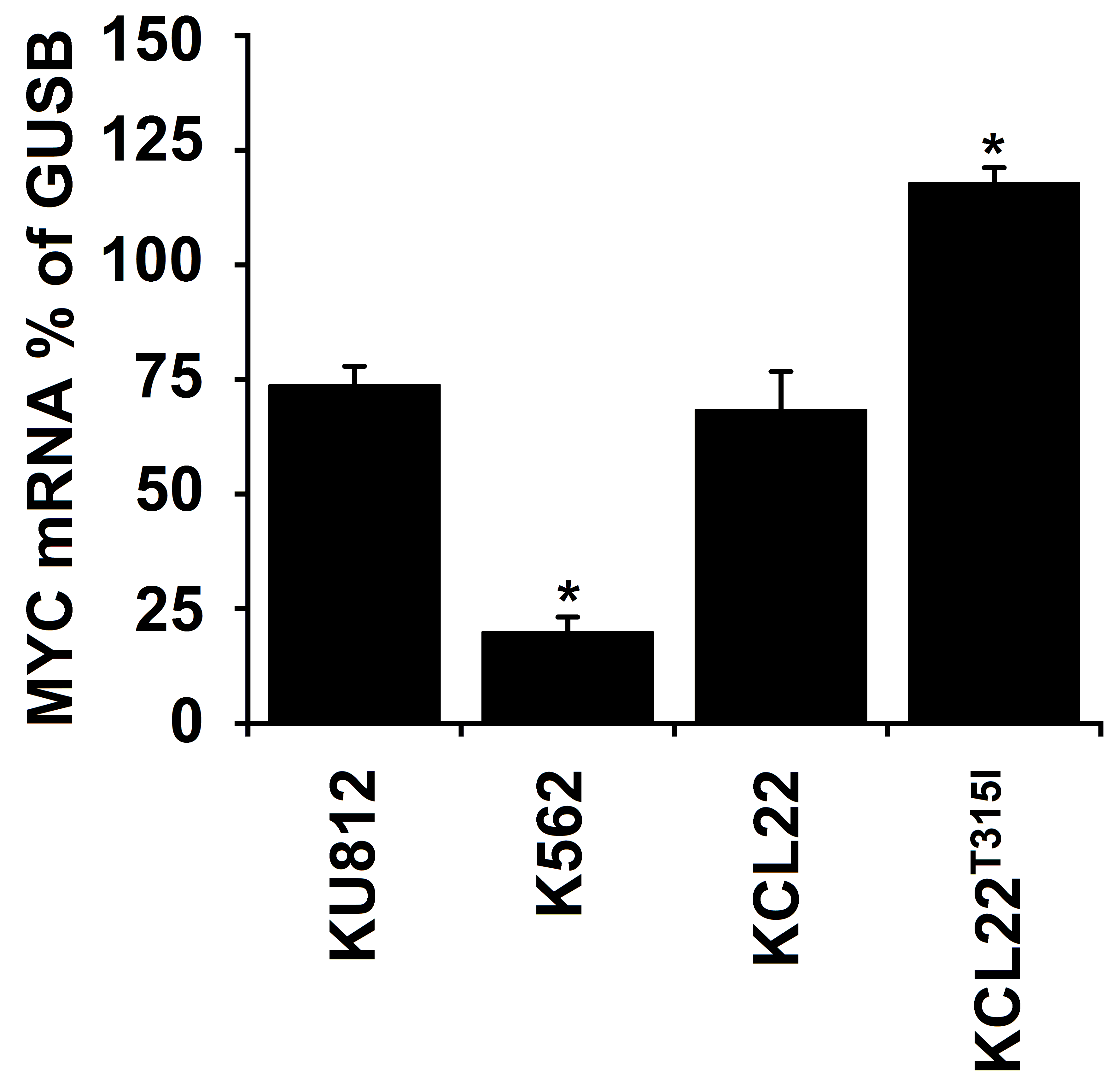


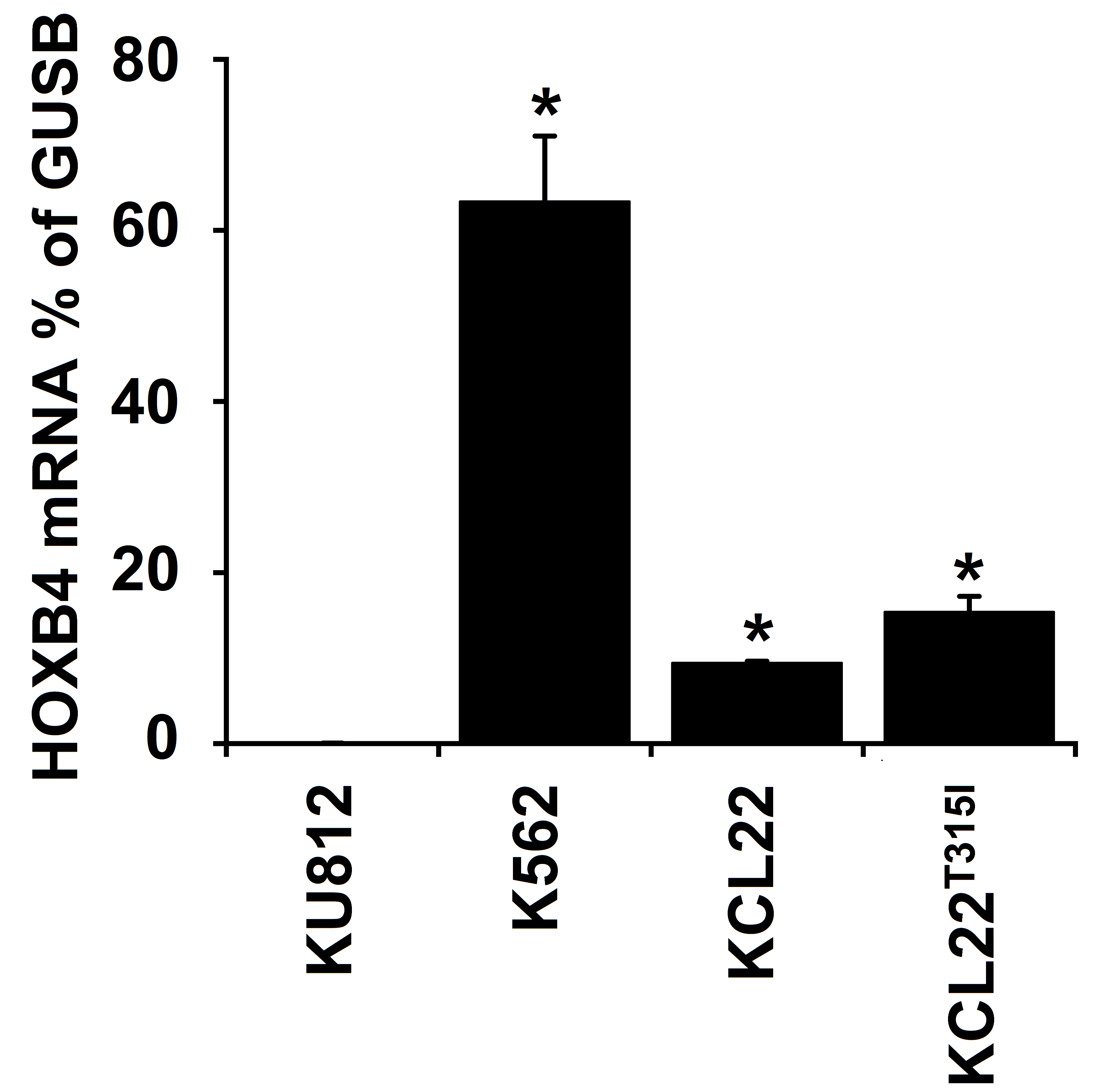


Peter et al. Supplemental Figure S6A


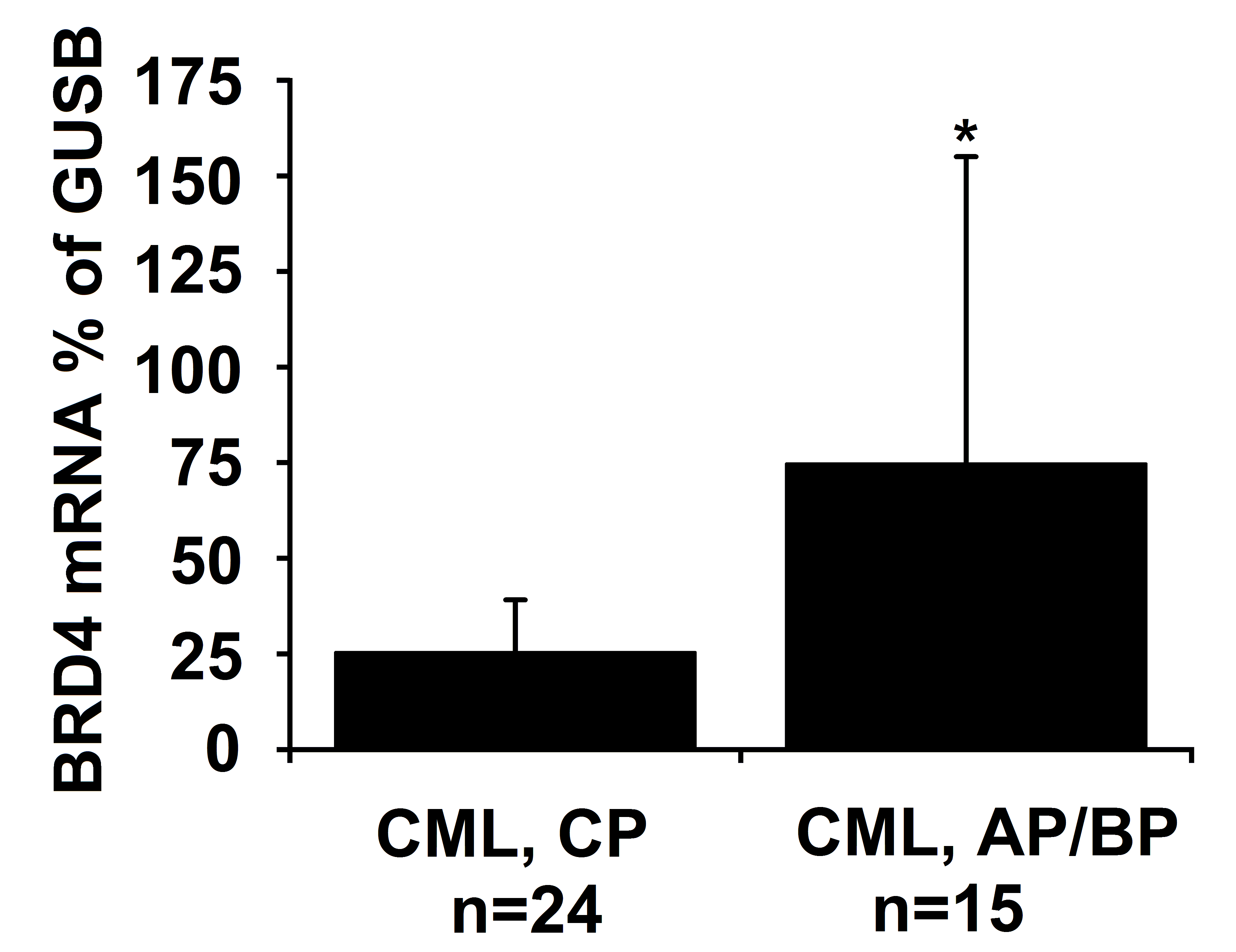

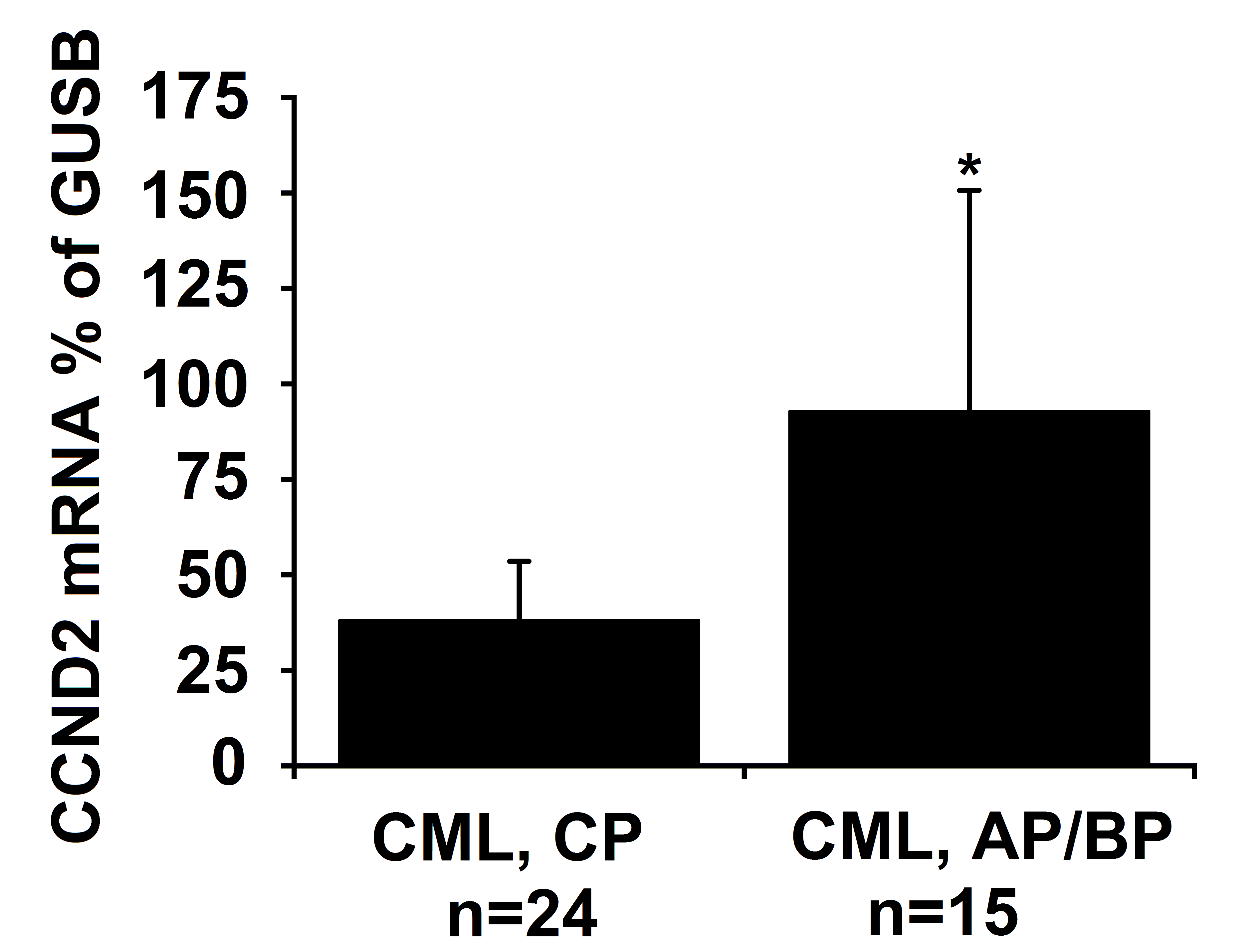

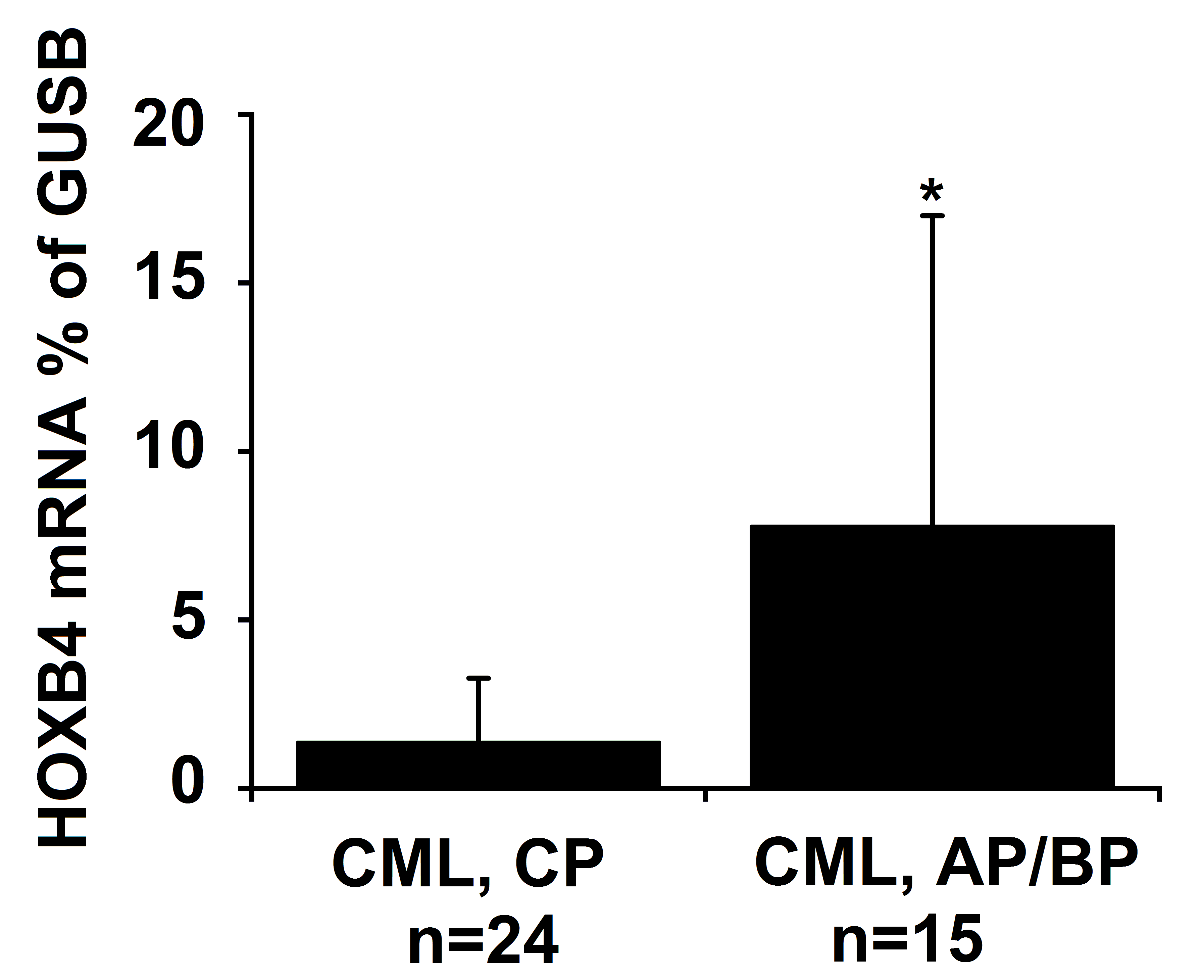

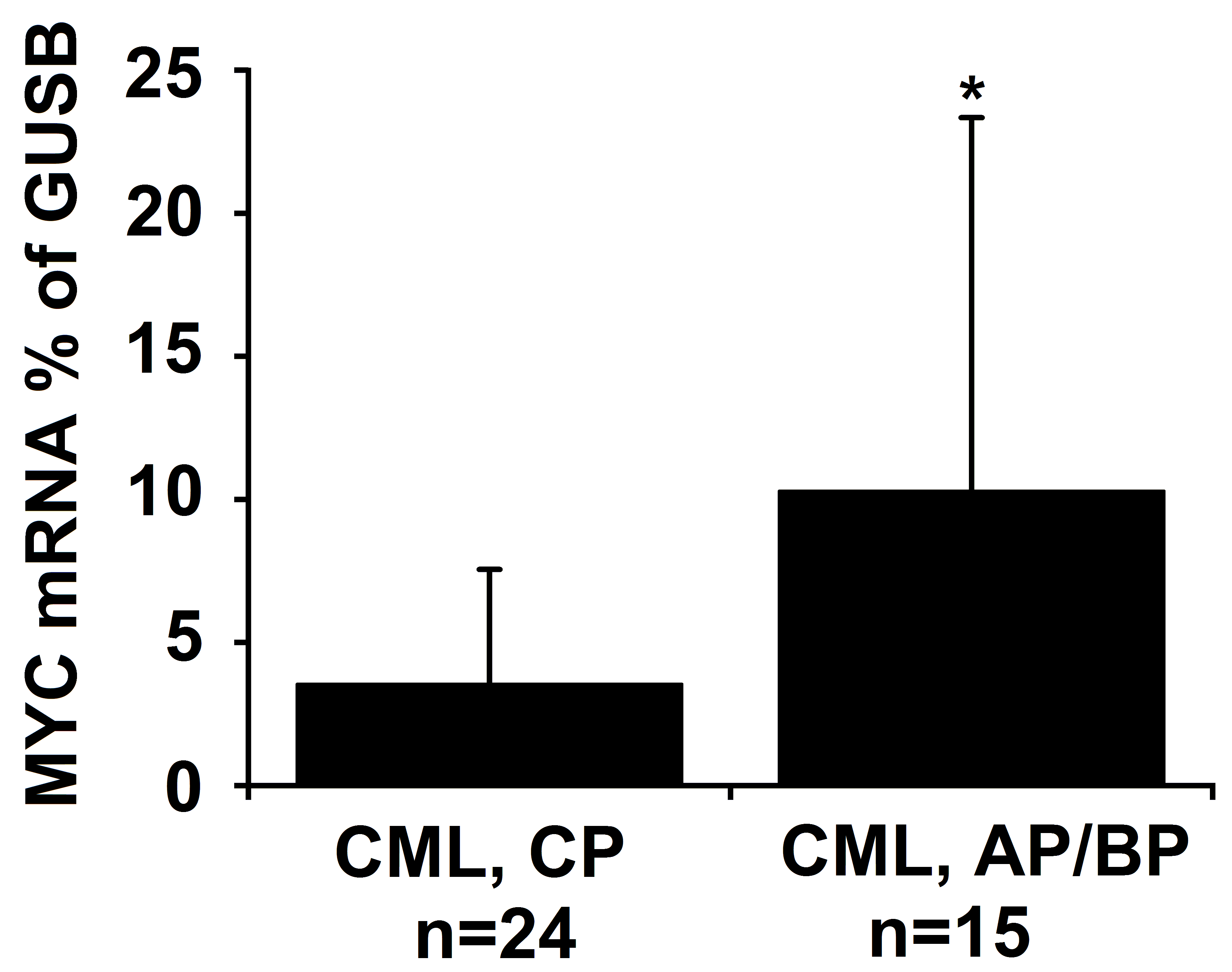


Peter et al. Supplemental Figure S6B


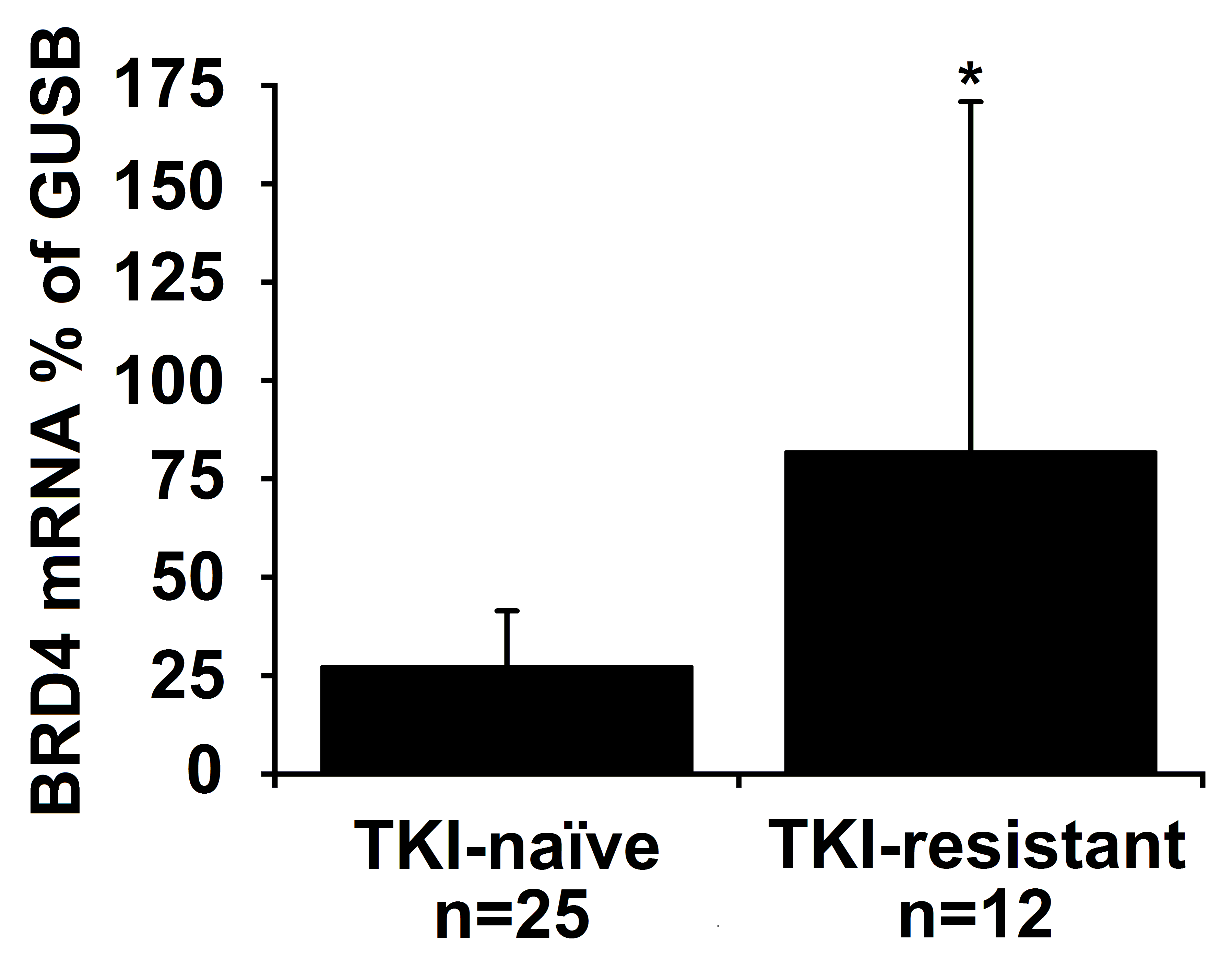

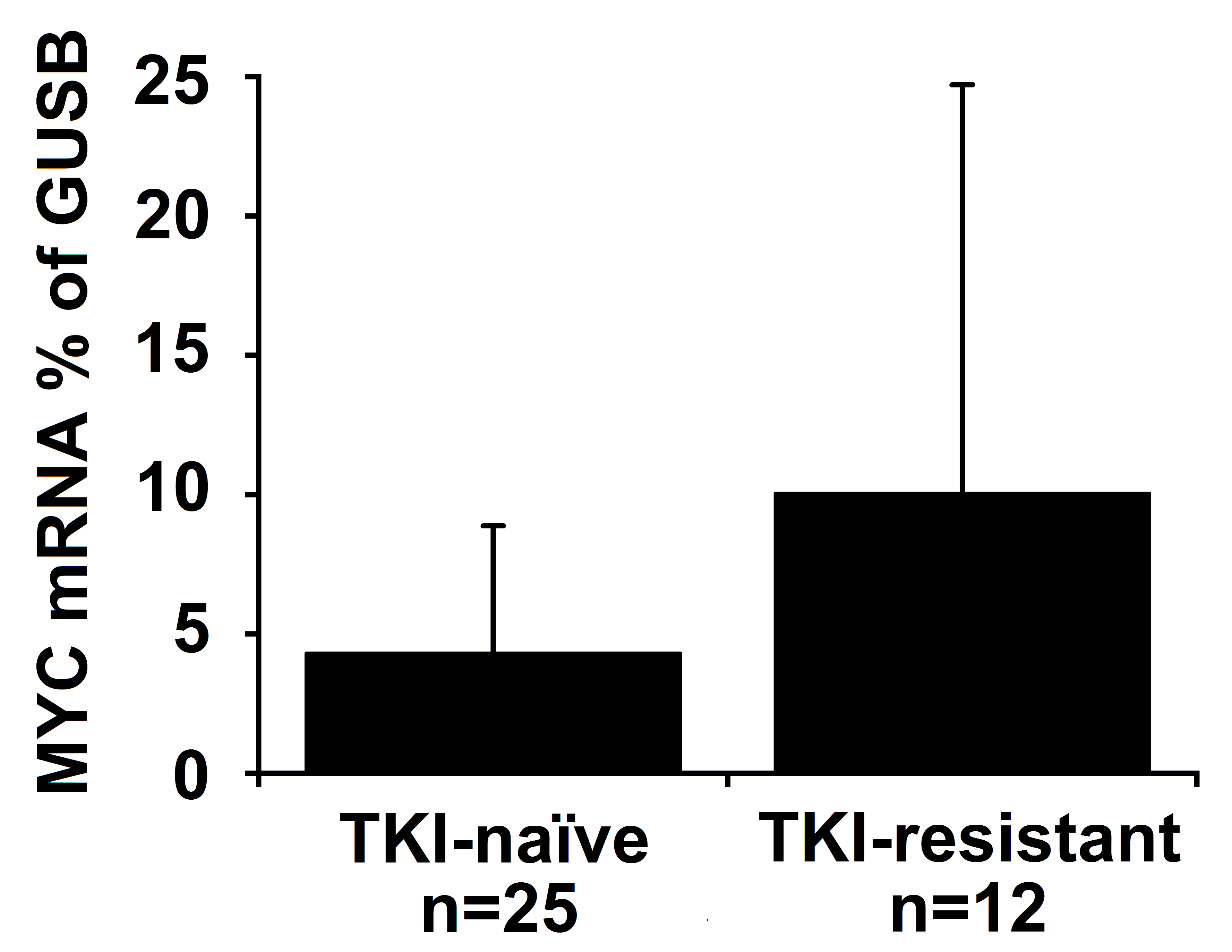


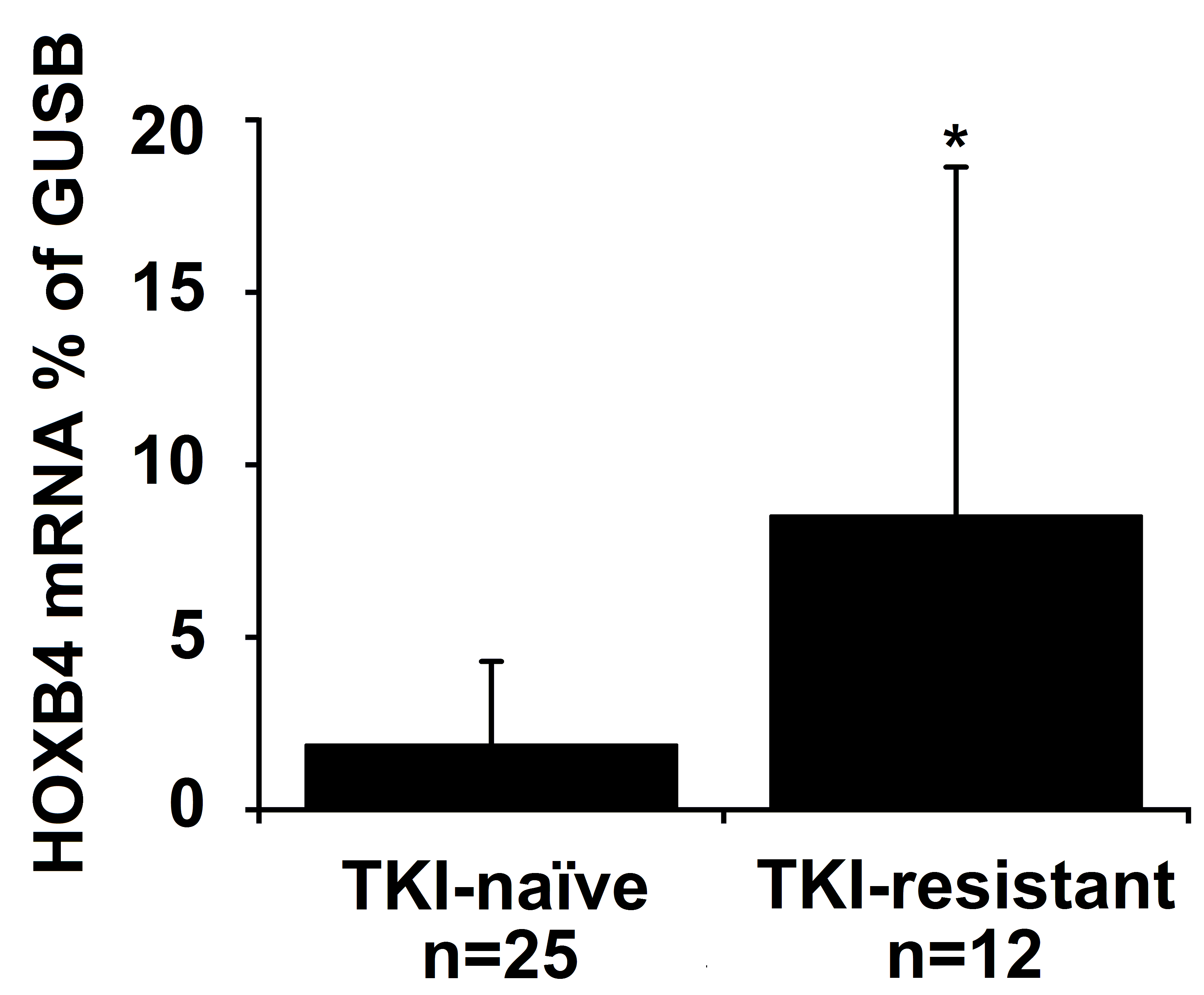

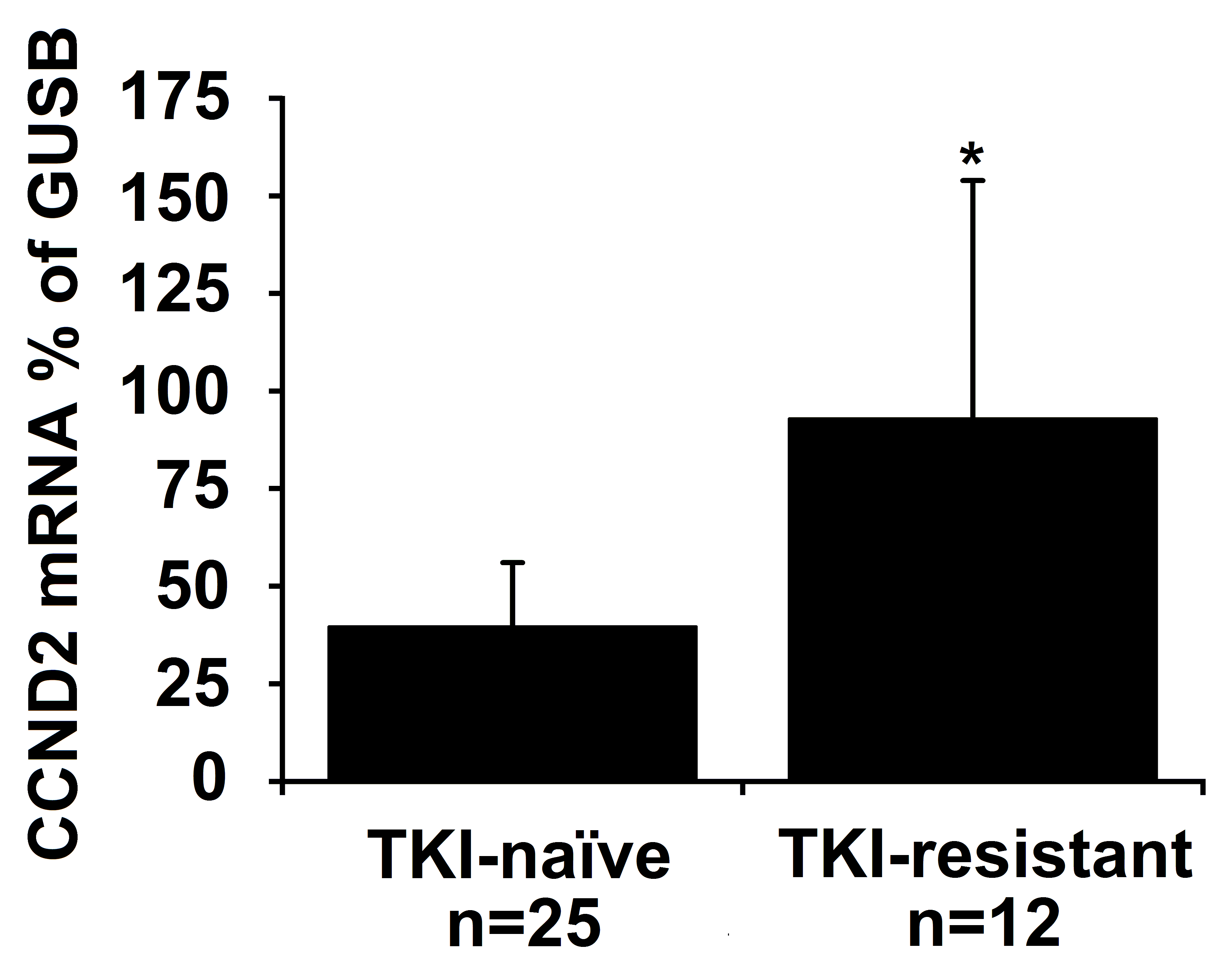


Peter et al. Supplemental Figure S6C


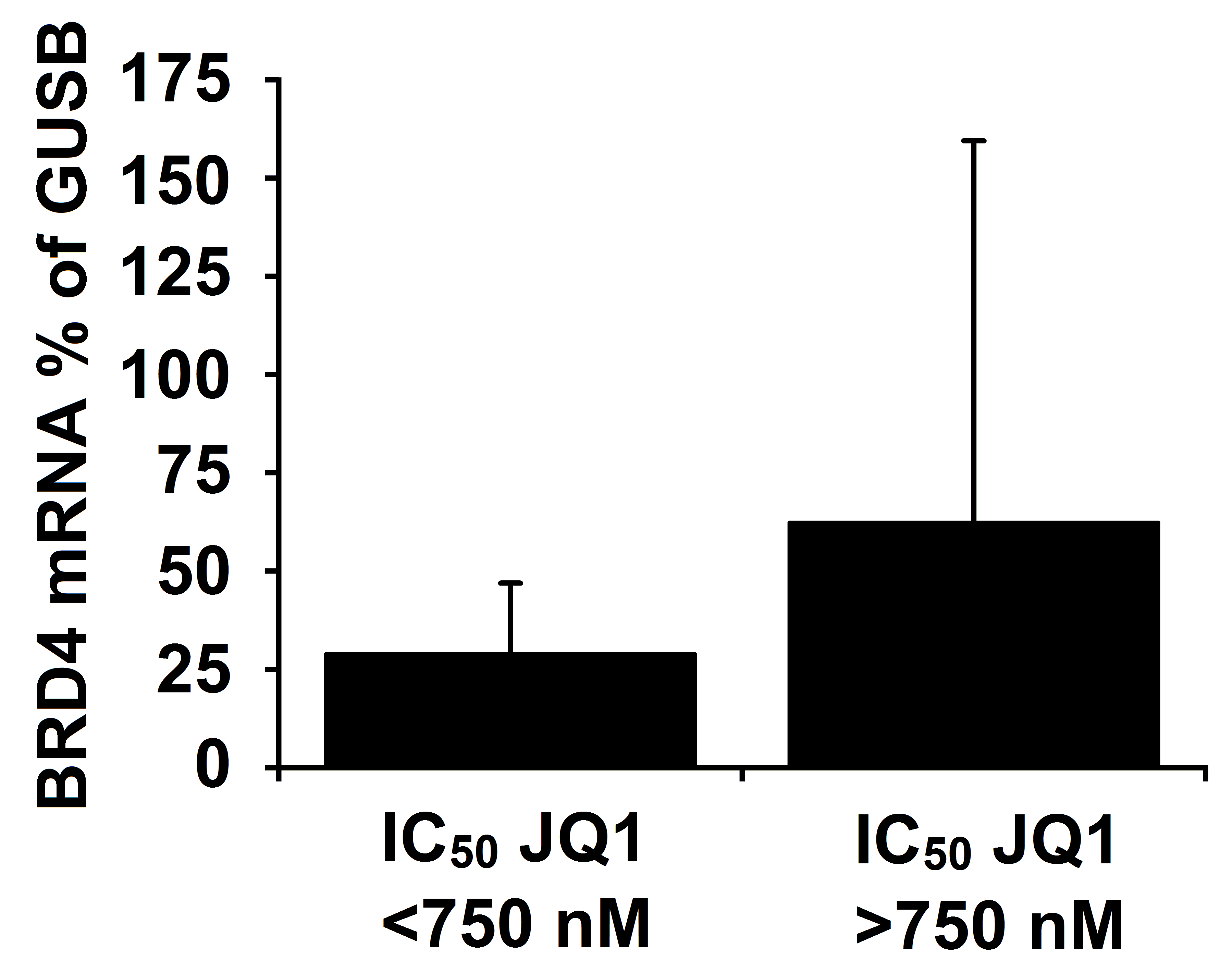

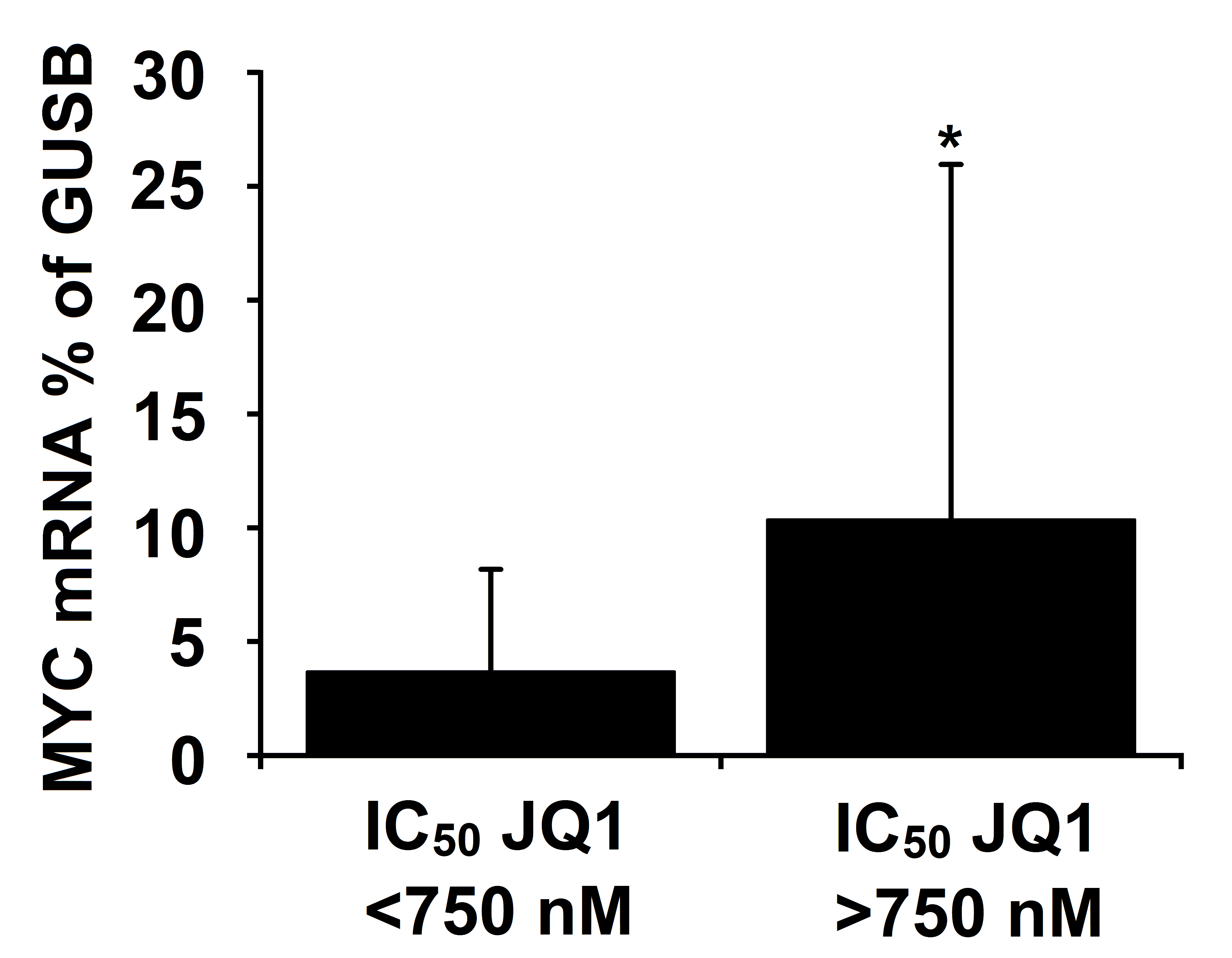


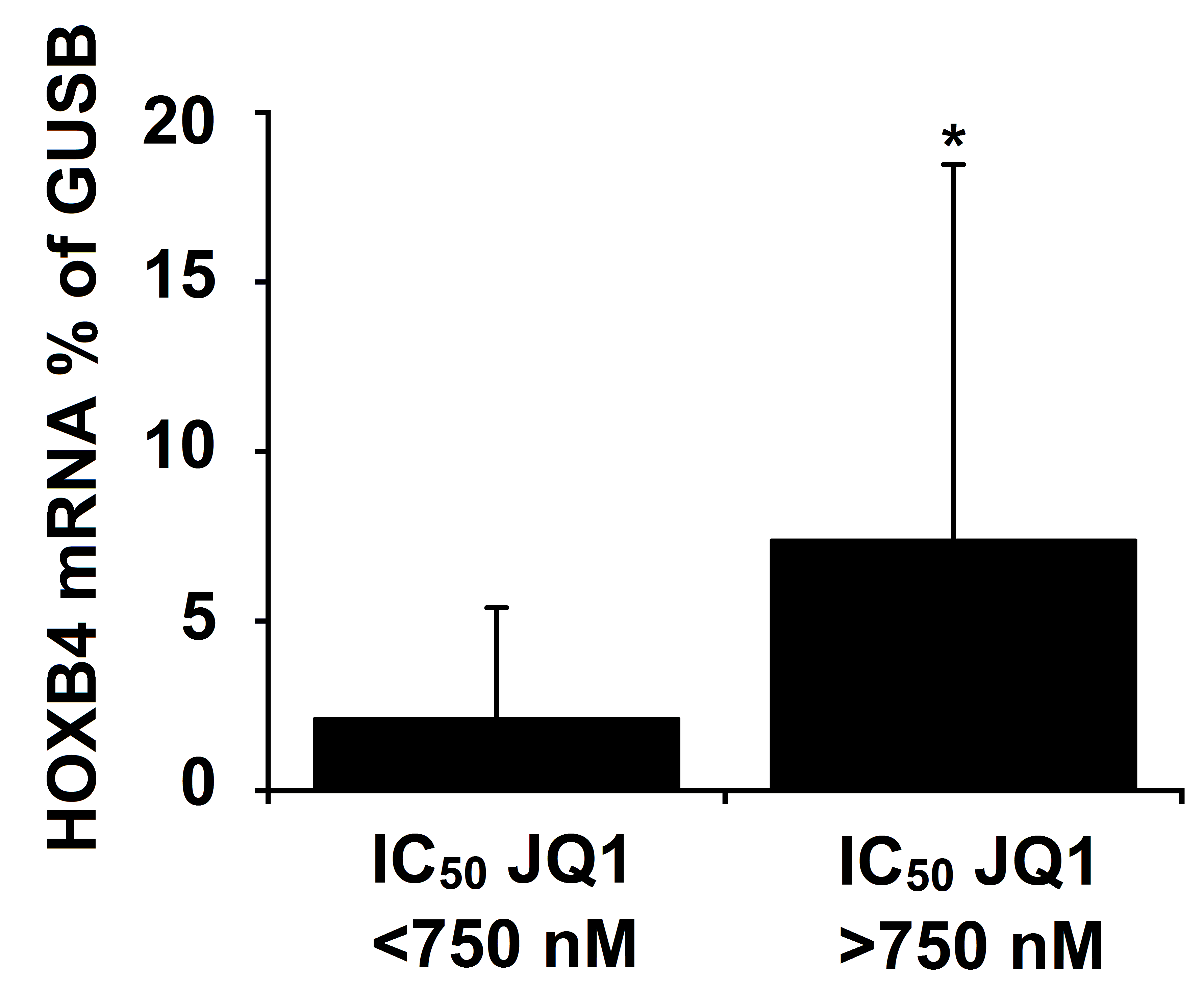

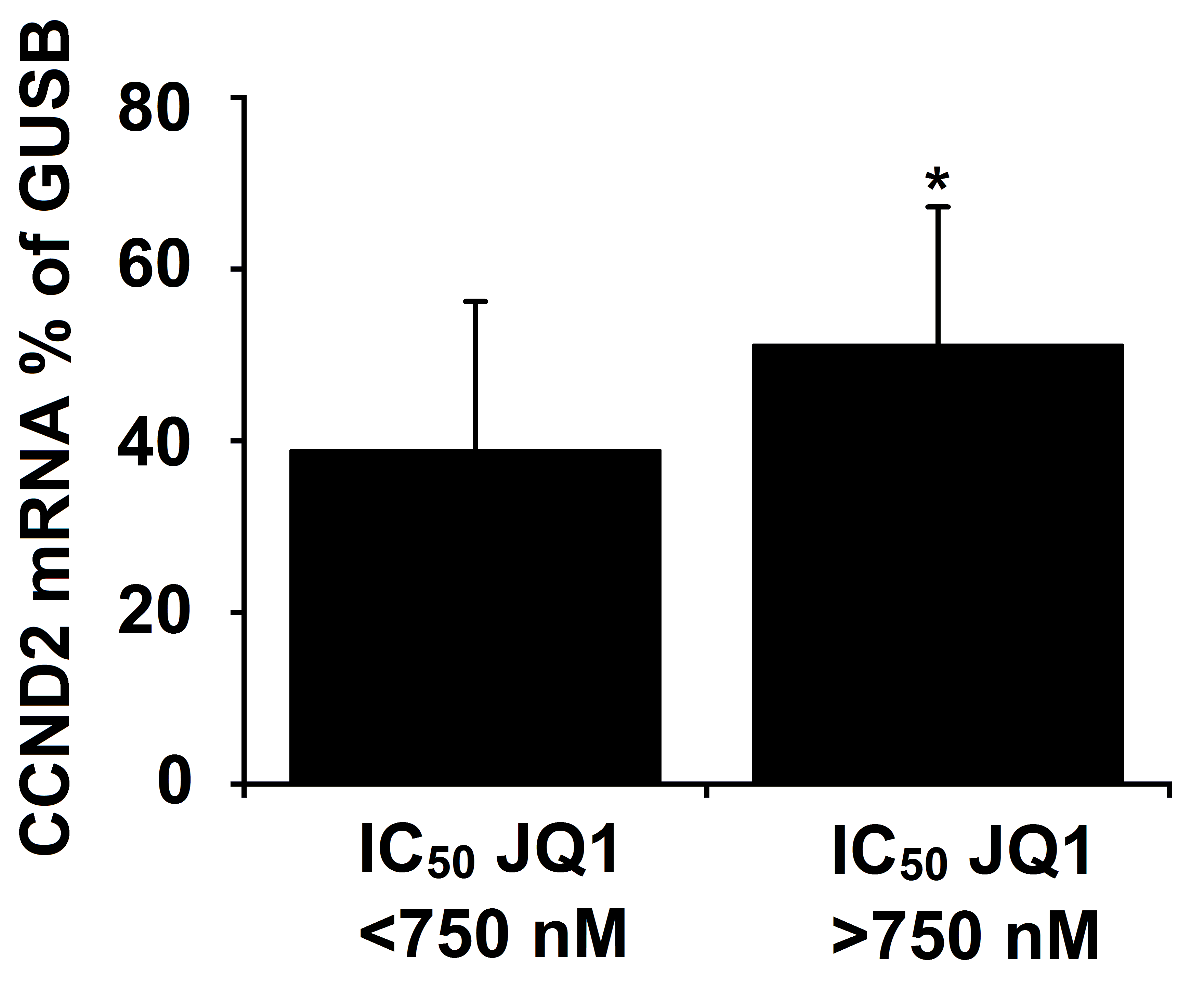


Peter et al. Supplemental Figure S6D

**Expression of BRD4, MYC and WNT signaling associated genes in CML cells**

A: KU812, K562, KCL22 and KCL22^T315I^ cells were analyzed for the expression of BRD4 mRNA and MYC mRNA (upper panel), and HOXB4 mRNA and CCND2 mRNA (lower panel) by qPCR as described in the text. Results (mRNA levels) are expressed as percent of GUSB mRNA levels and represent the mean±SD from 3 independent experiments. mRNA expression levels obtained in JQ1-responsive KU812 cells were compared to mRNA levels in JQ1-resistant cells; asterisk: p<0.05 compared to KU812 cells. B: Primary CML MNC were analyzed for the expression of BRD4 mRNA and MYC mRNA (upper panel) and HOXB4 mRNA and CCND2 mRNA (upper panel) by qPCR. mRNA levels are expressed as percent of GUSB mRNA levels and represent the mean±SD from 24 CML CP samples or 15 CML AP/BP samples (AP, n=2; BP, n=13). Higher levels of BRD4, MYC, HOXB4, and CCND2 mRNA levels were found to be expressed in leukemic cells in AP/BP patients compared to cells of CP patients (*, p<0.05 by Mann-Whitney-U-Test). C: Primary CML MNC obtained from CML patients without therapy (TKI-naïve) or TKI-resistant CML were analyzed for the expression of BRD4 mRNA and MYC mRNA (upper panel) and HOXB4 mRNA and CCND2 mRNA (lower panel) by qPCR. mRNA levels are expressed as percent of GUSB mRNA levels and represent the mean±SD from 25 TKI-naïve CML cell samples or 12 TKI-resistant CML cell samples. Asterisk: p<0.05 by Mann-Whitney-U-Test. D: JQ1-sensitive primary CML cell samples (IC_50_ <750 nM, n=22) and JQ1-resistant CML cell samples (IC_50_ >750 nM, n=10) were analyzed for the expression of BRD4 mRNA and MYC mRNA (upper panel), and HOXB4 mRNA and CCND2 mRNA (lower panel) by qPCR. mRNA levels are expressed as percent of GUSB mRNA levels and represent the mean±SD from 22 JQ1-sensitive CML cell samples and 10 JQ1-resistant CML cell samples. Asterisk: p<0.05 by Mann-Whitney-U-Test.

Supplemental Figure S7


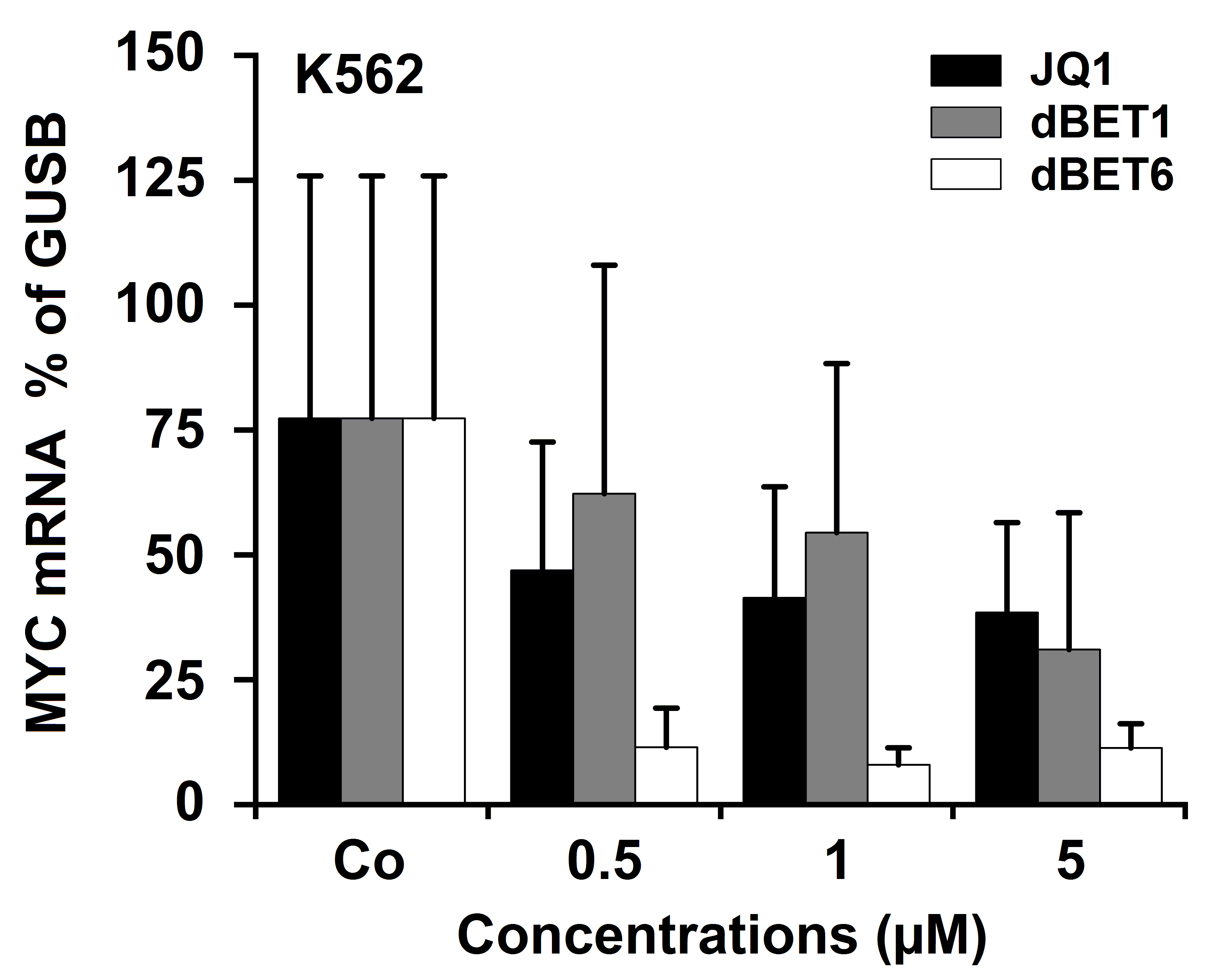

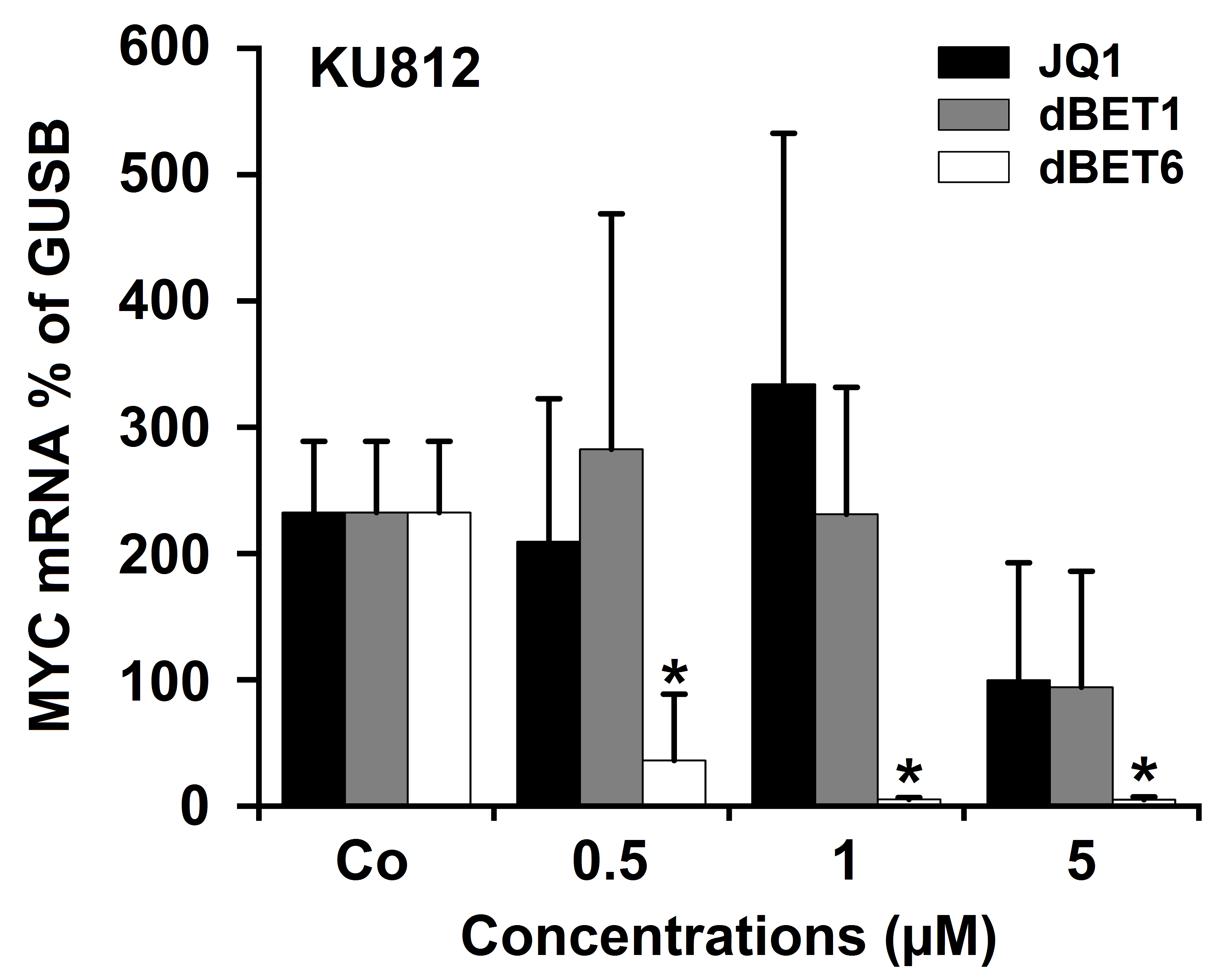


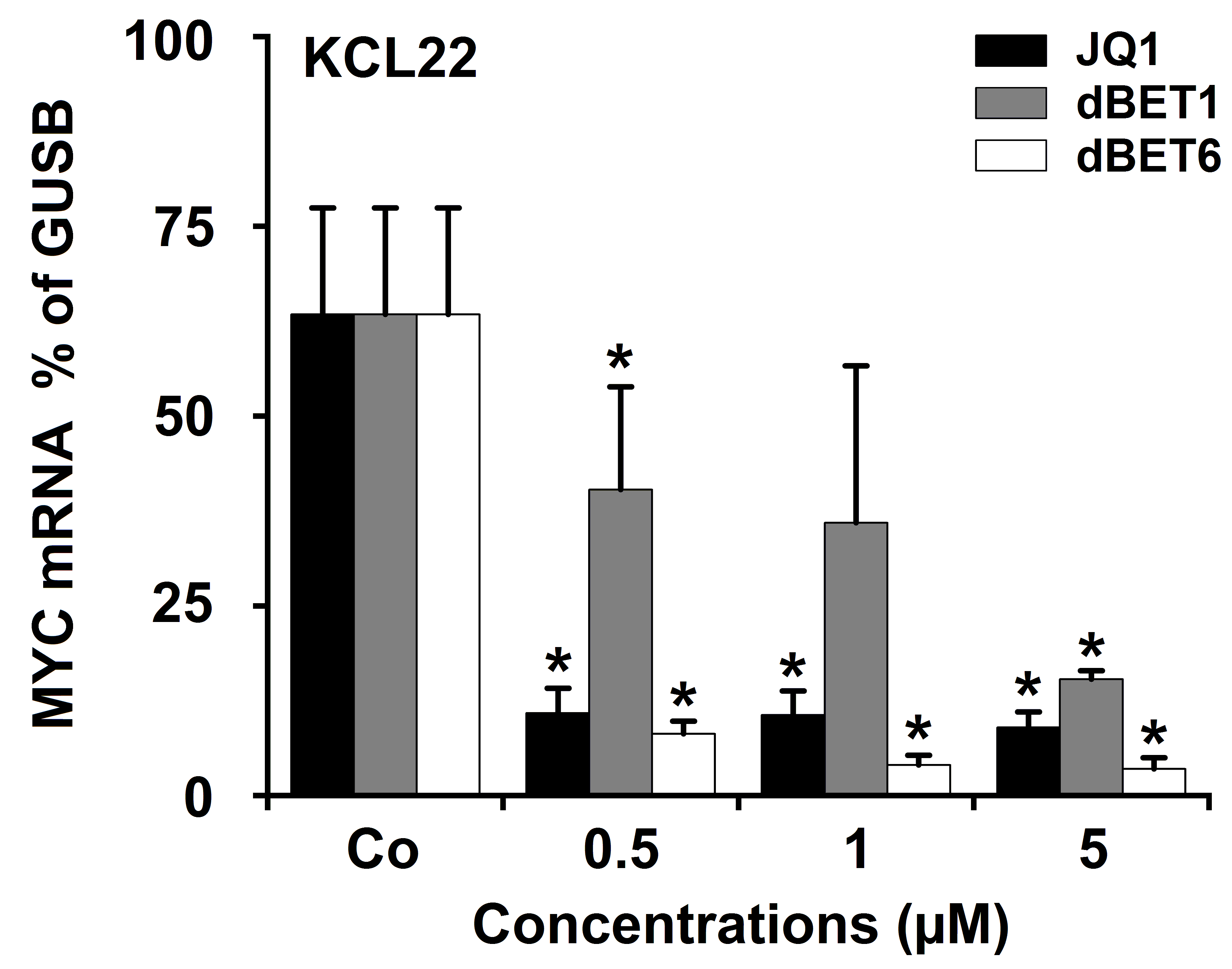

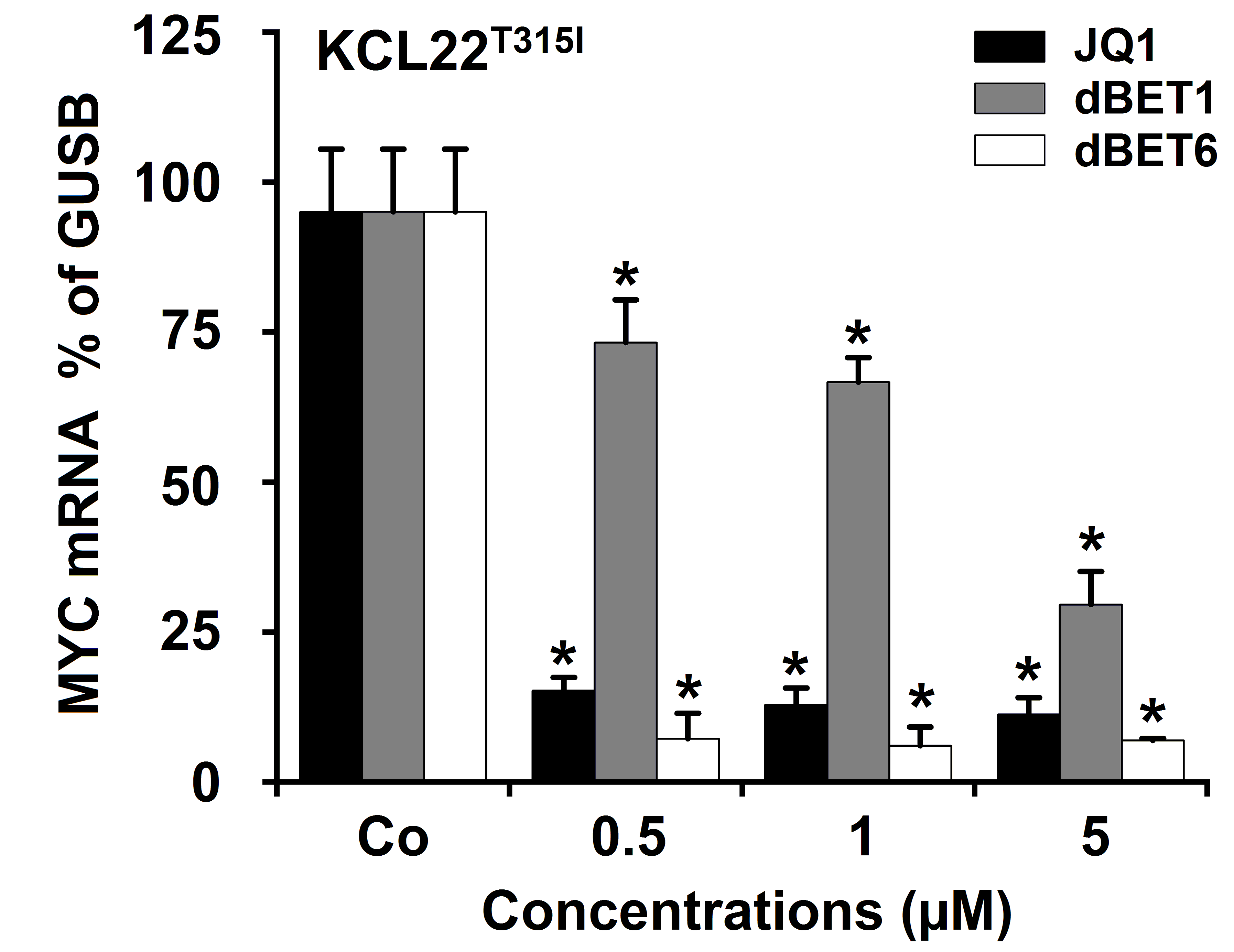


Peter et al Supplemental Figure S7A


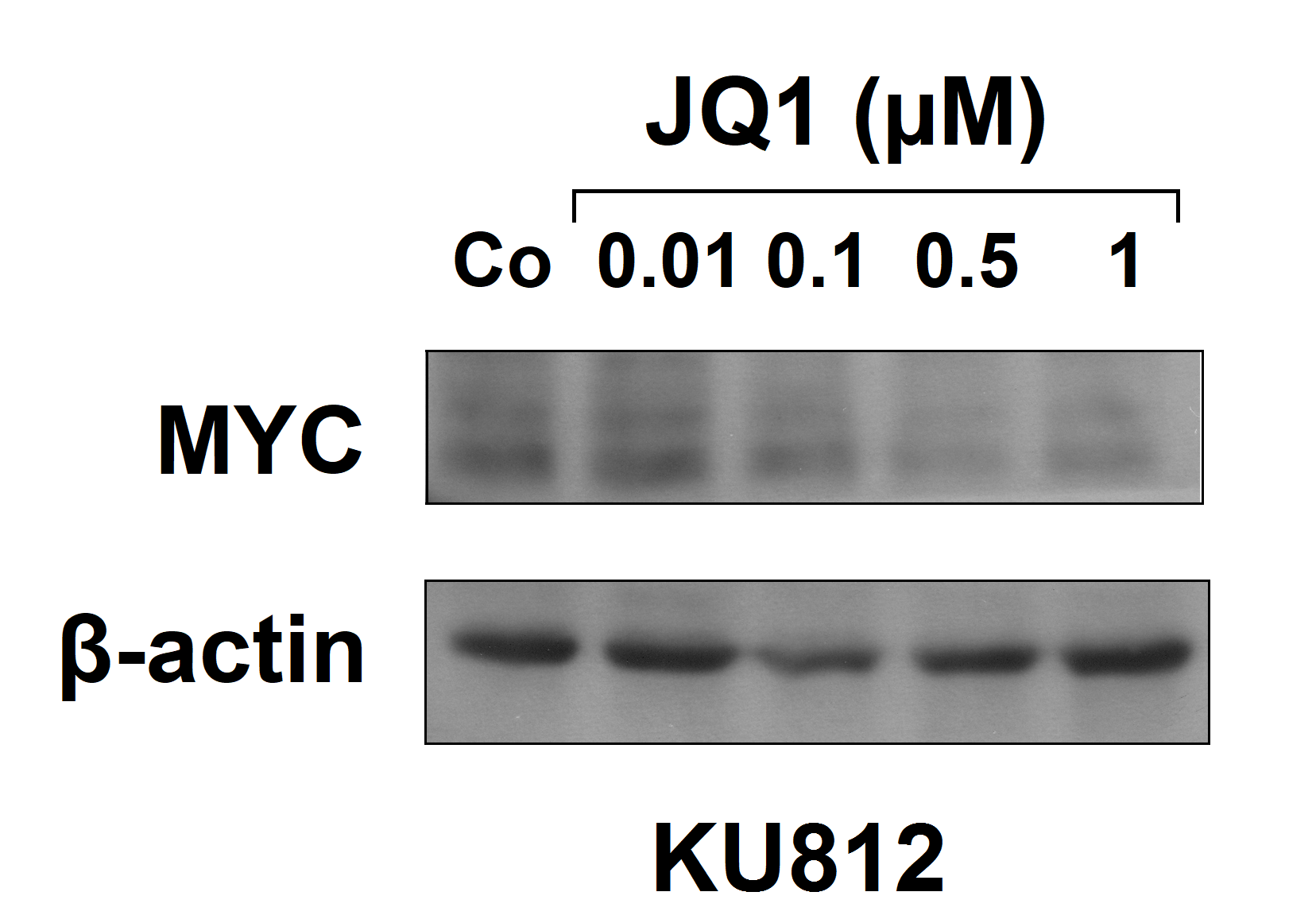

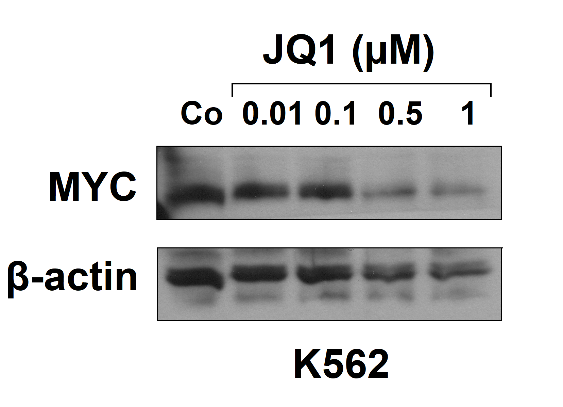


Peter et al Supplemental Figure S7B


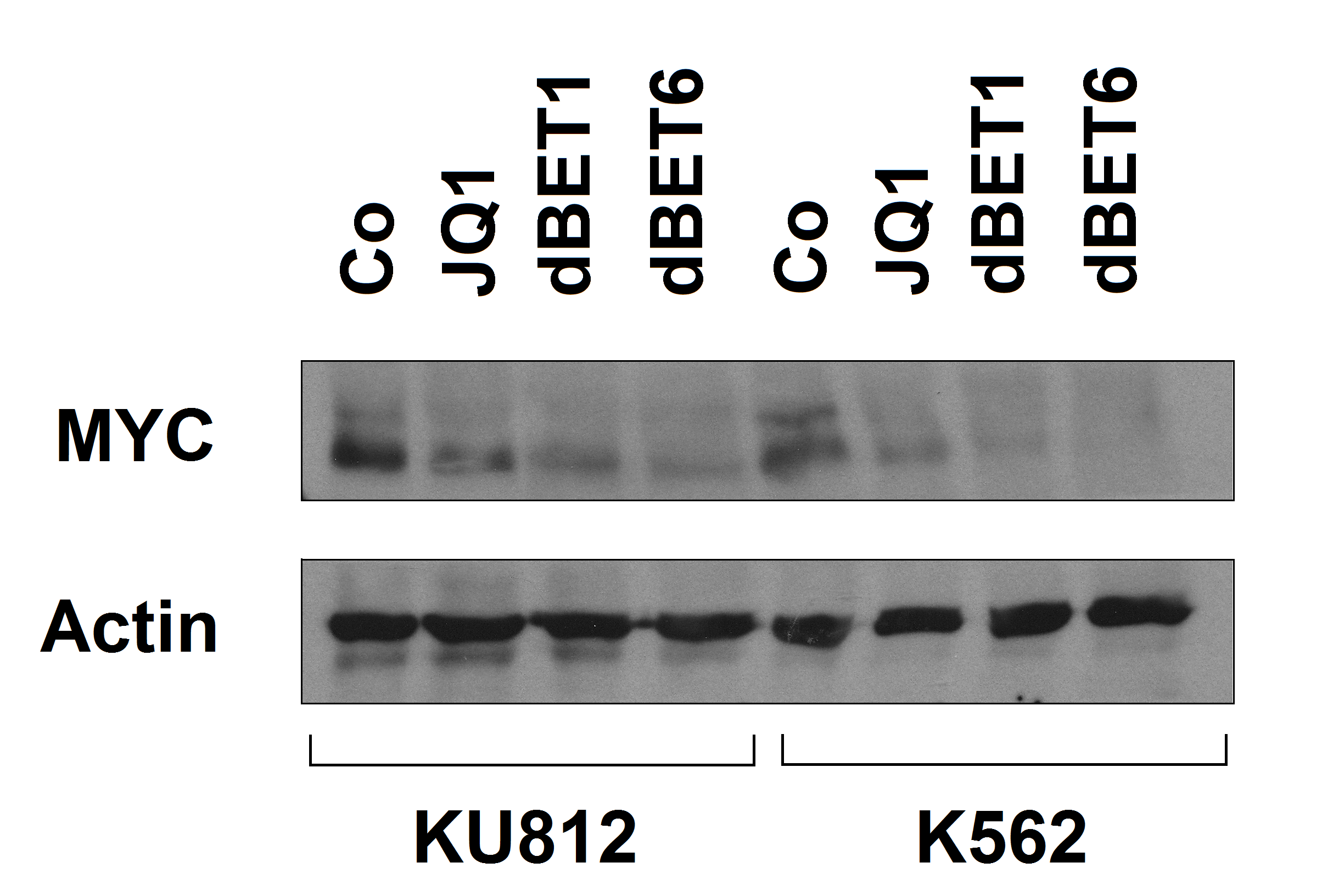

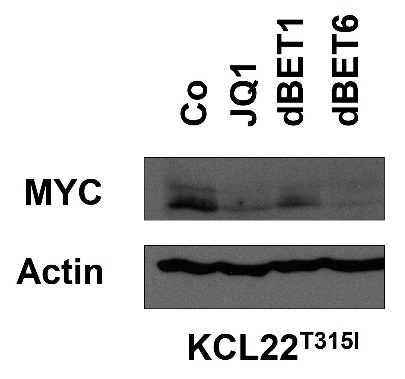

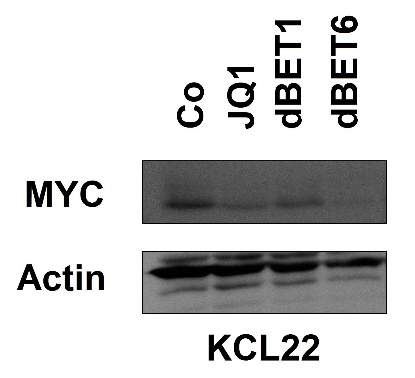


Peter et al Supplemental Figure S7C


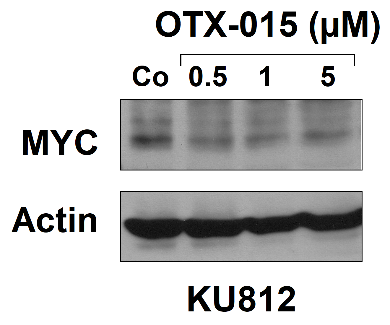

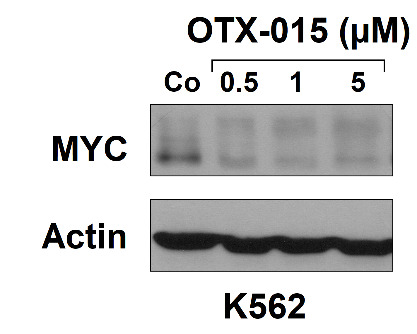


Peter et al Supplemental Figure S7D


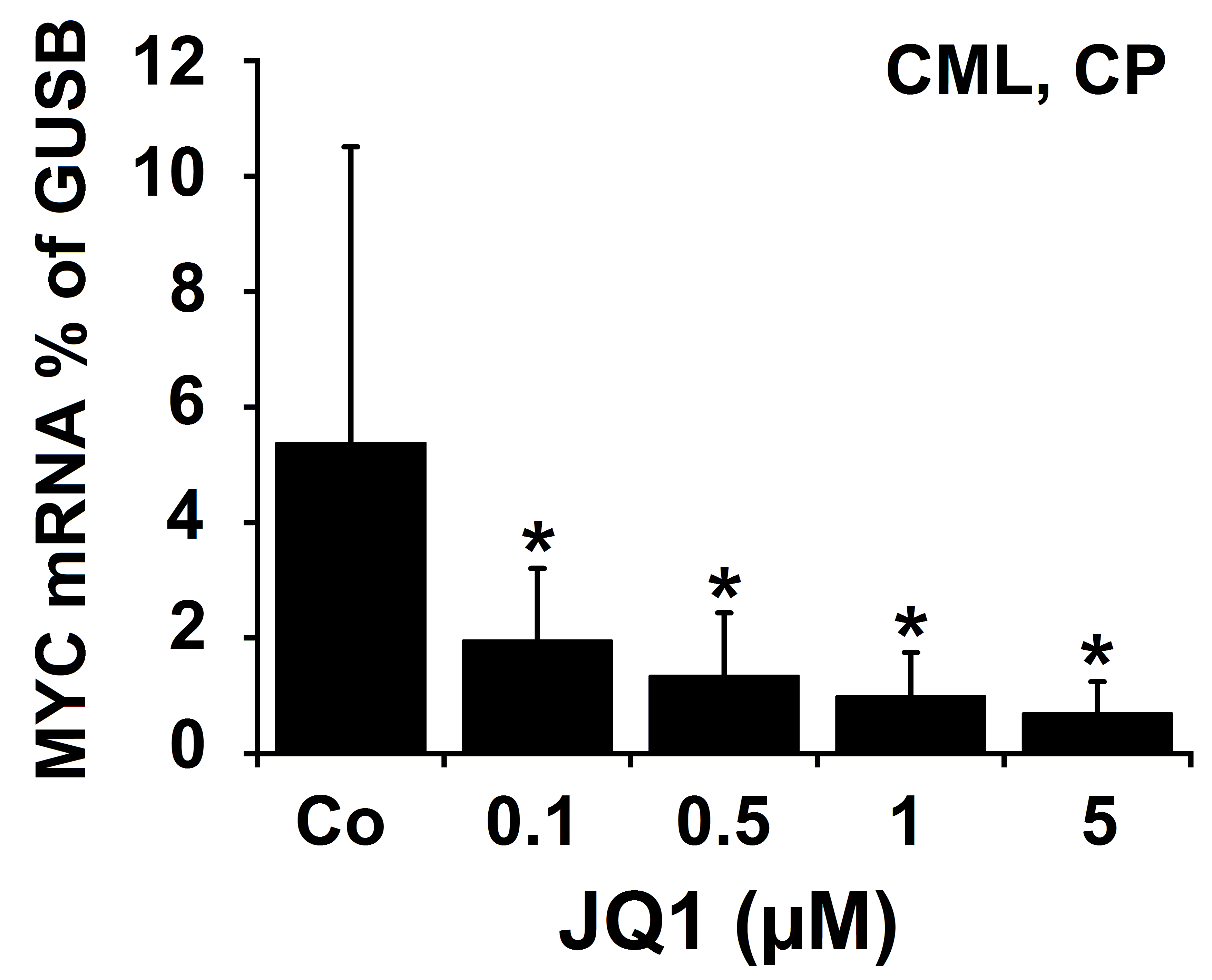


Peter et al. Supplemental Figure S7E


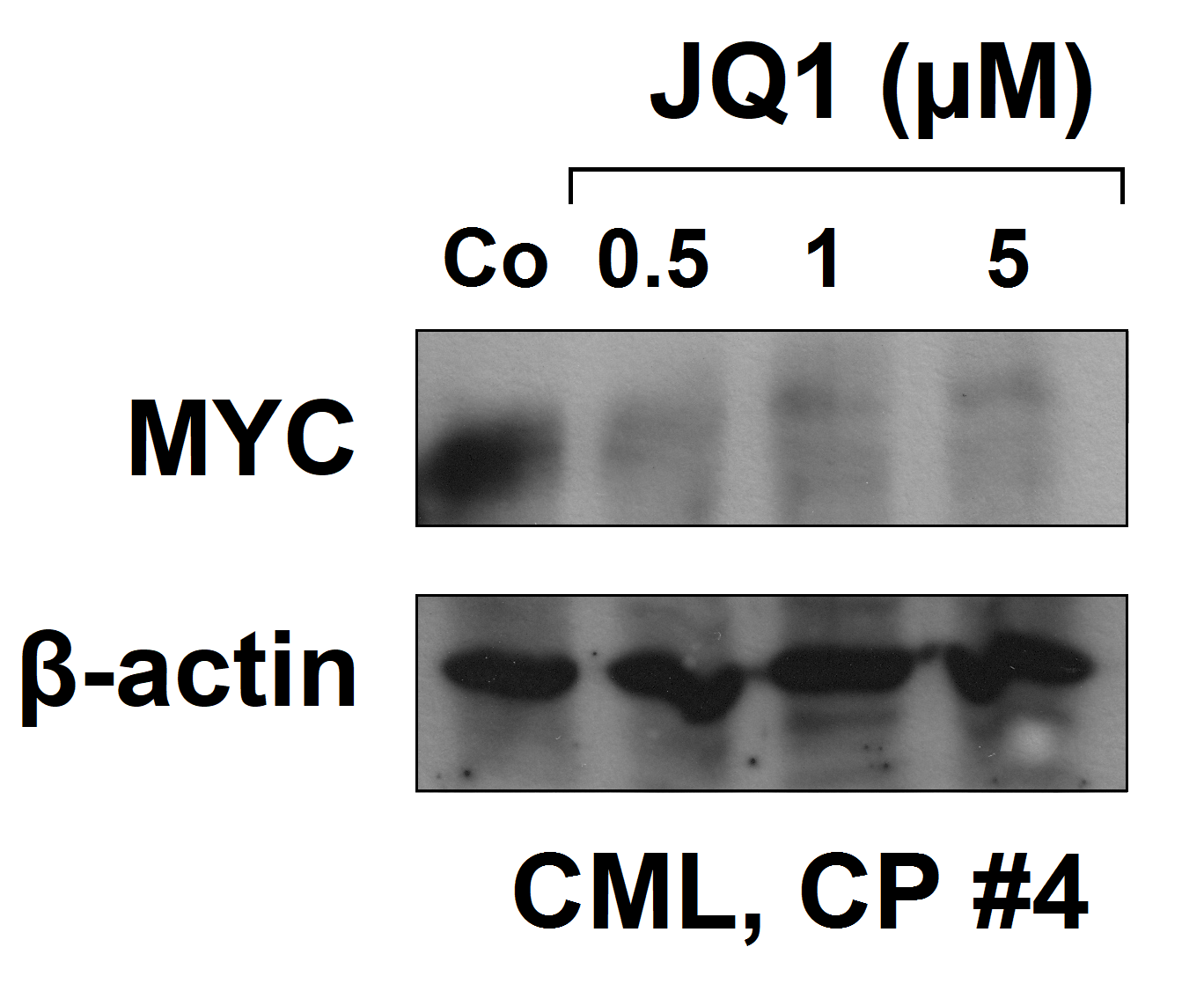


Peter et al., Supplemental Figure S7F

**Effects of BET inhibitors on MYC expression in CML cells**

A: KU812, K562, KCL22, and KCL22^T315I^ cells were incubated in medium (Co) or in medium containing various concentrations of JQ1, dBET1 or dBET6 as indicated at 37°C for 24 hours. Thereafter, cells were harvested and analyzed for MYC mRNA expression by qPCR as described. Results are expressed as MYC mRNA levels percent of GUSB mRNA levels and represent the mean±SD from 3 independent experiments. Asterisk: p<0.05 compared to control. B: For detection of MYC protein, Western blotting was performed using antibodies directed against MYC and β-actin (loading control). C: KU812, K562, KCL22, and KCL22^T315I^ cells were incubated in JQ1, dBET1, or dBET6 (each 1 µM) for 24 hours. Thereafter, Western blotting was performed using antibodies directed against MYC or Actin (loading control). D: KU812 cells and K562 cells were incubated with medium (Co) or medium containing various concentrations of OTX-015 as indicated at 37°C for 24 hours. Thereafter, cells were harvested and Western blotting was performed using antibodies against MYC and Actin. E: Primary CML MNC (CML CP, n=9) were incubated in medium (Co) or medium containing various concentrations of JQ1 as indicated at 37°C for 24 hours. Thereafter, cells were harvested and analyzed for MYC mRNA expression by qPCR as described. Results are expressed as MYC mRNA levels percent of GUSB mRNA levels and represent the mean±SD from 9 independent experiments. Asterisk: p<0.05 compared to control. F: Primary CML MNC (CML CP) were incubated in medium (Co) or medium containing various concentrations JQ1 for 24 hours. Western blotting was performed using antibodies directed against MYC and β-actin (loading control).

Supplemental Figure S8


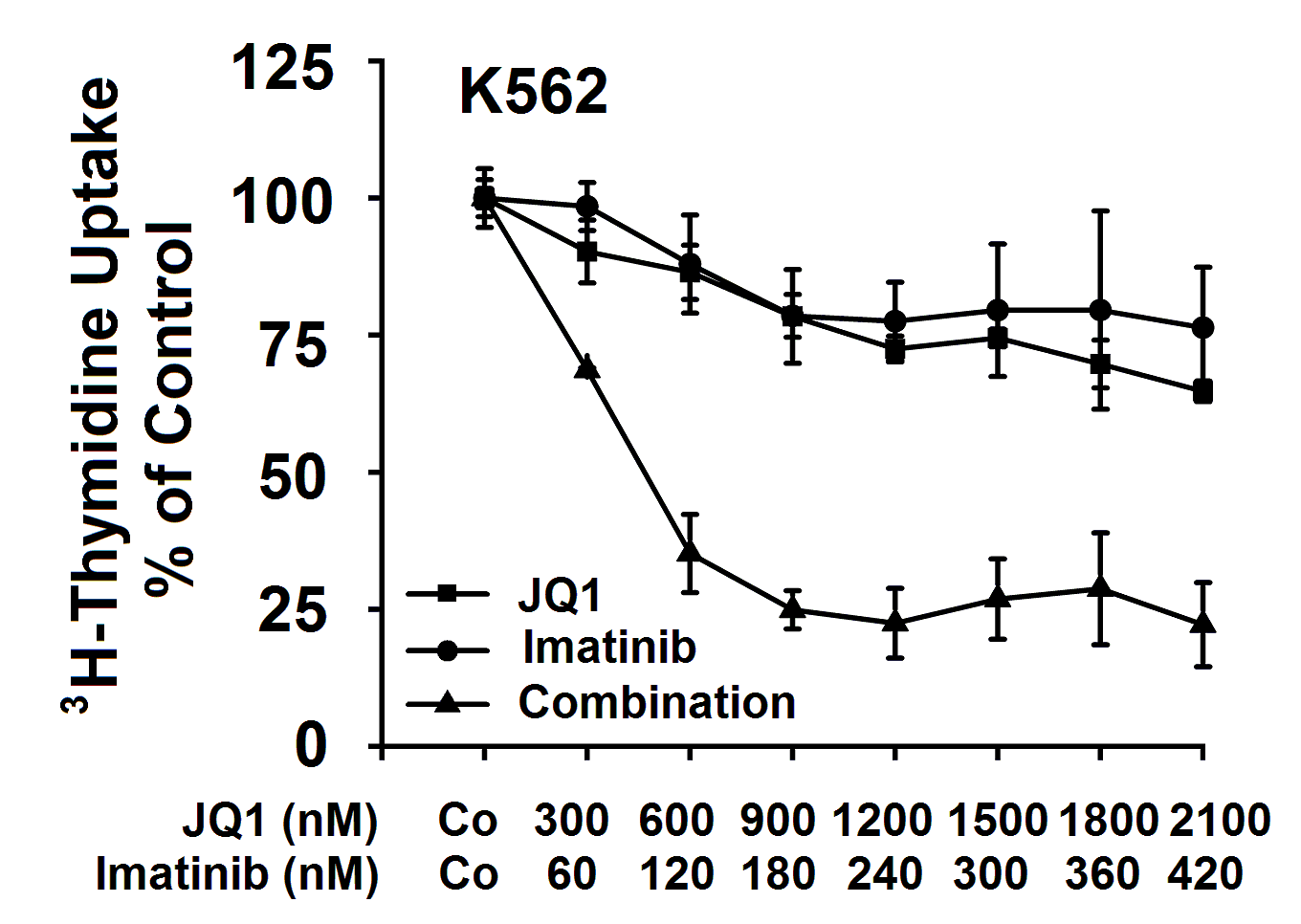

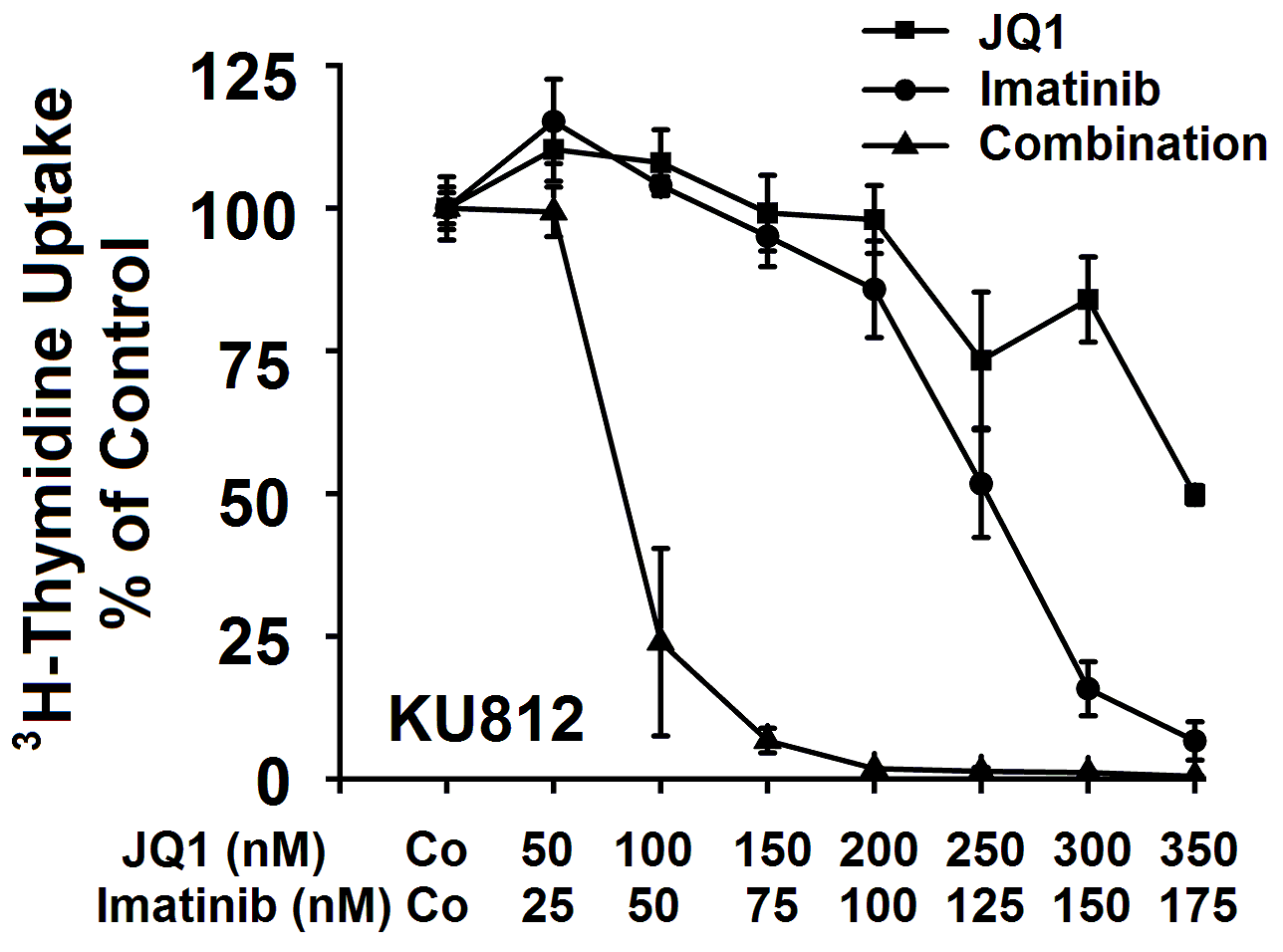


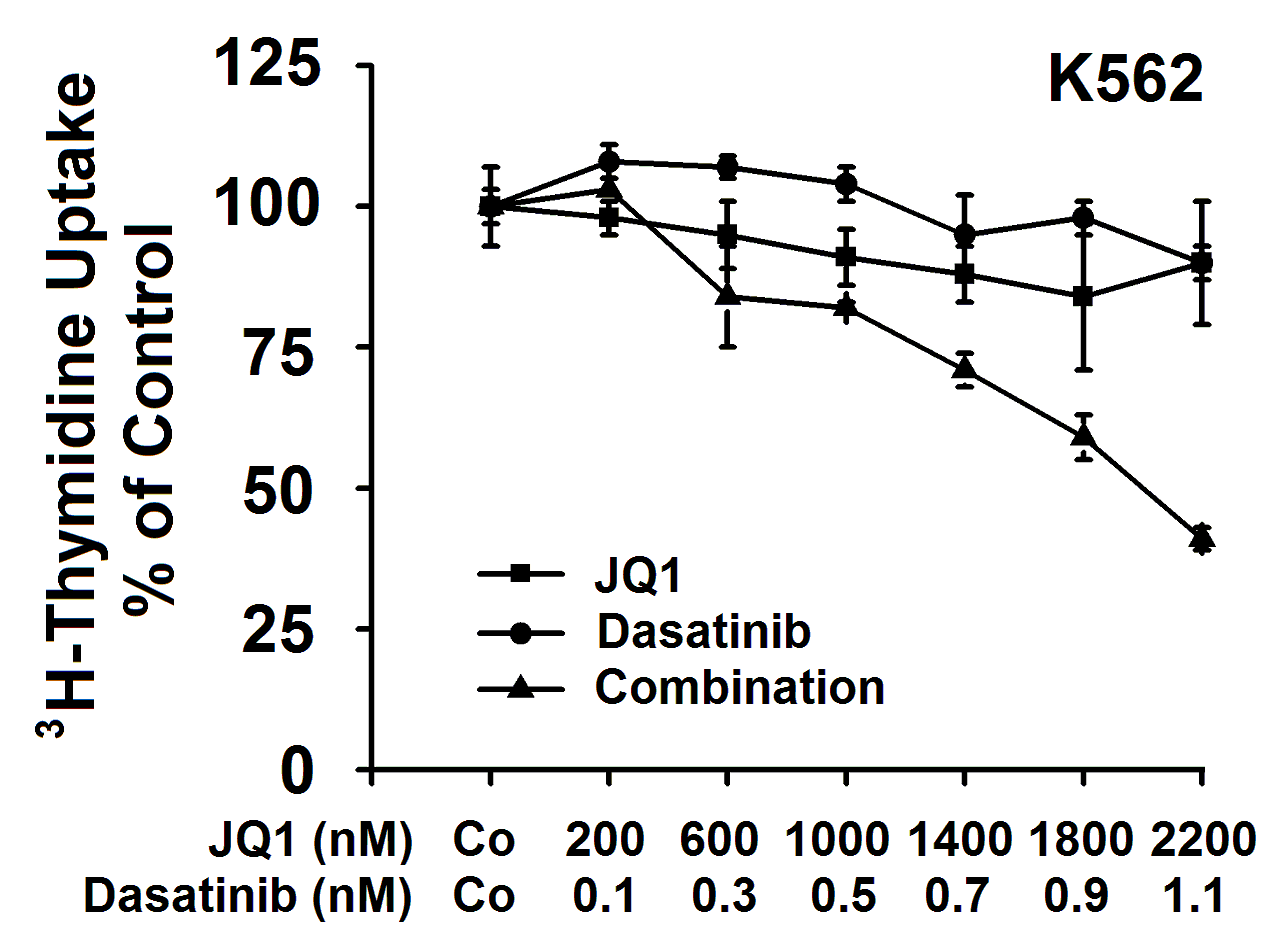


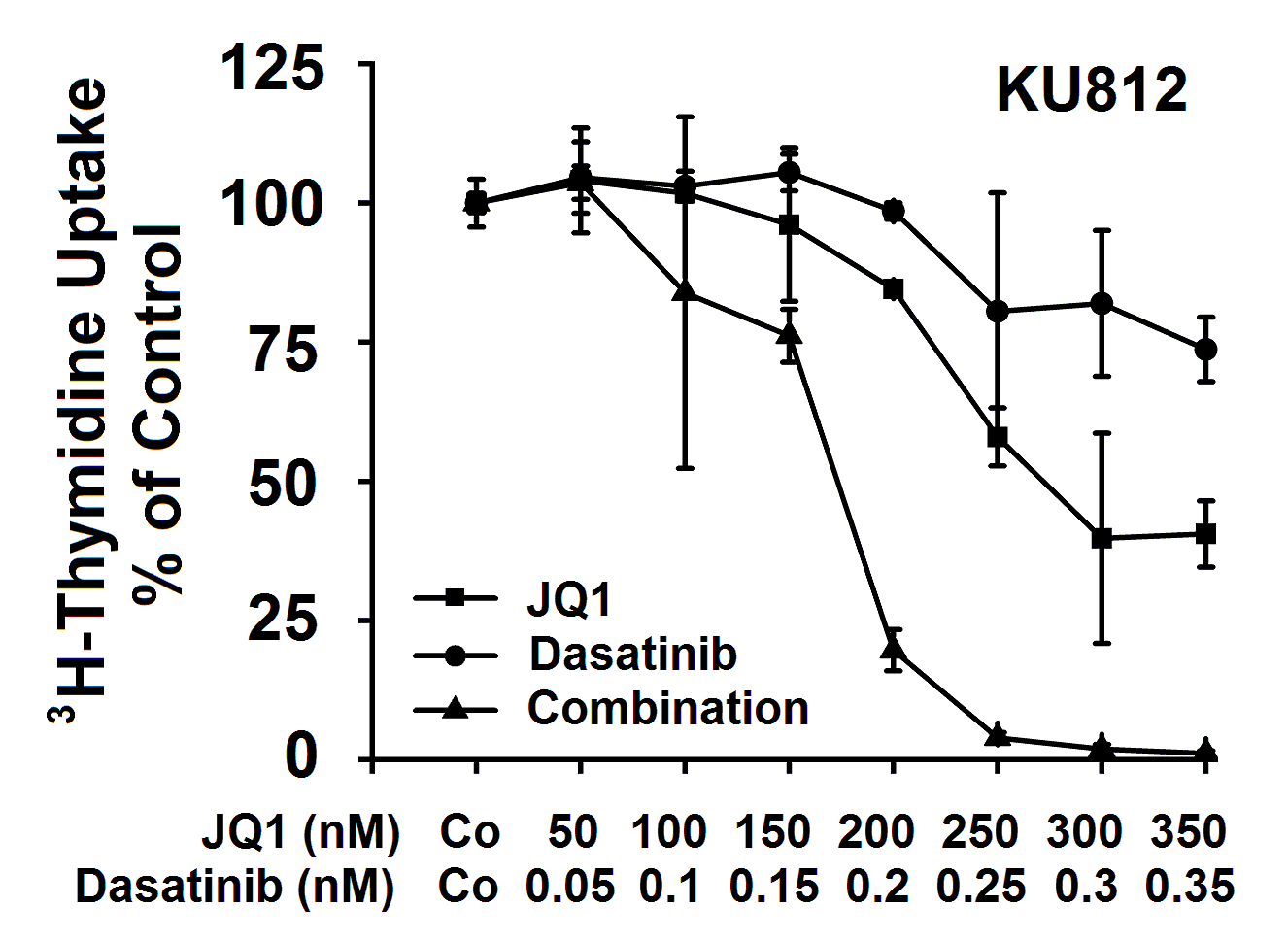


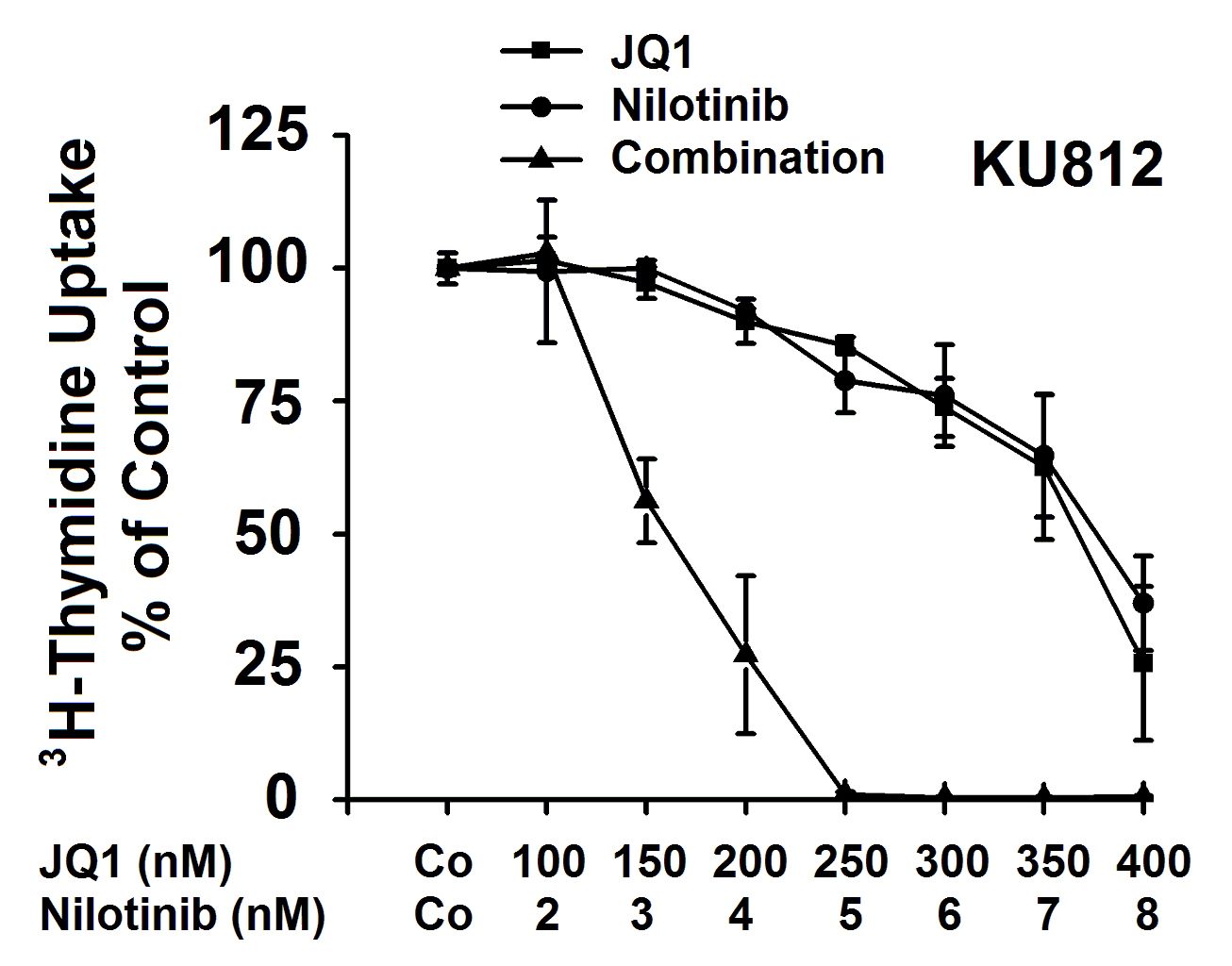


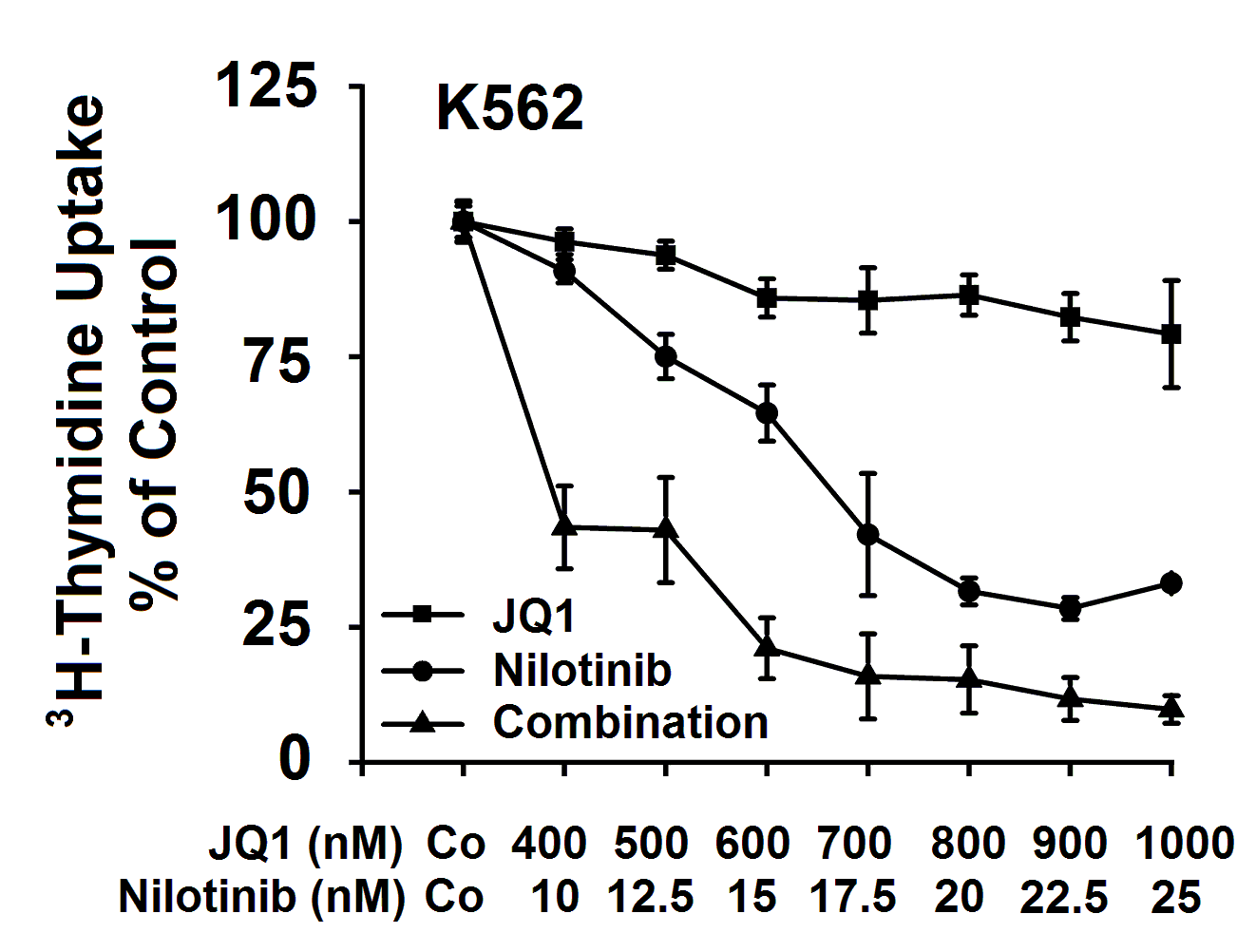


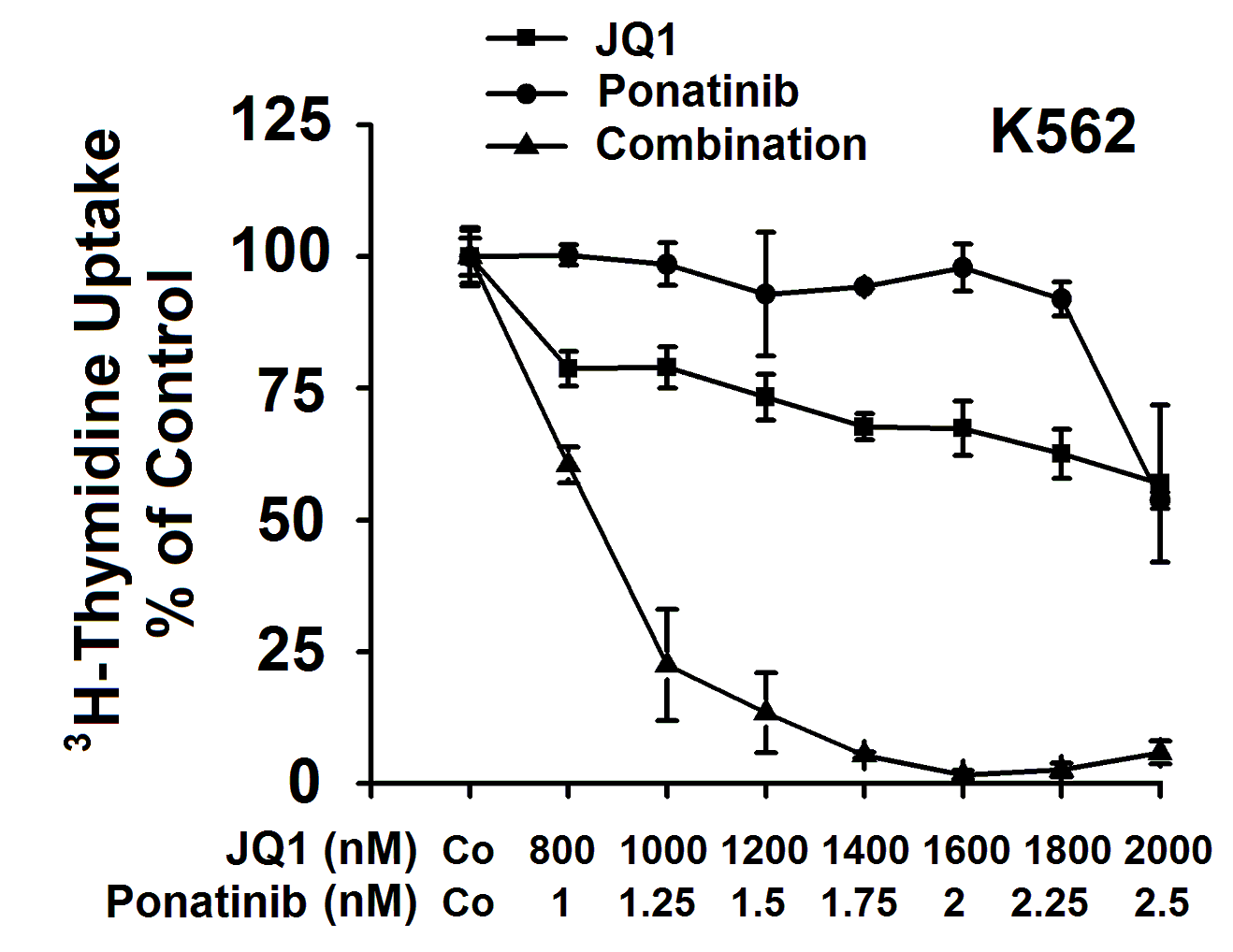

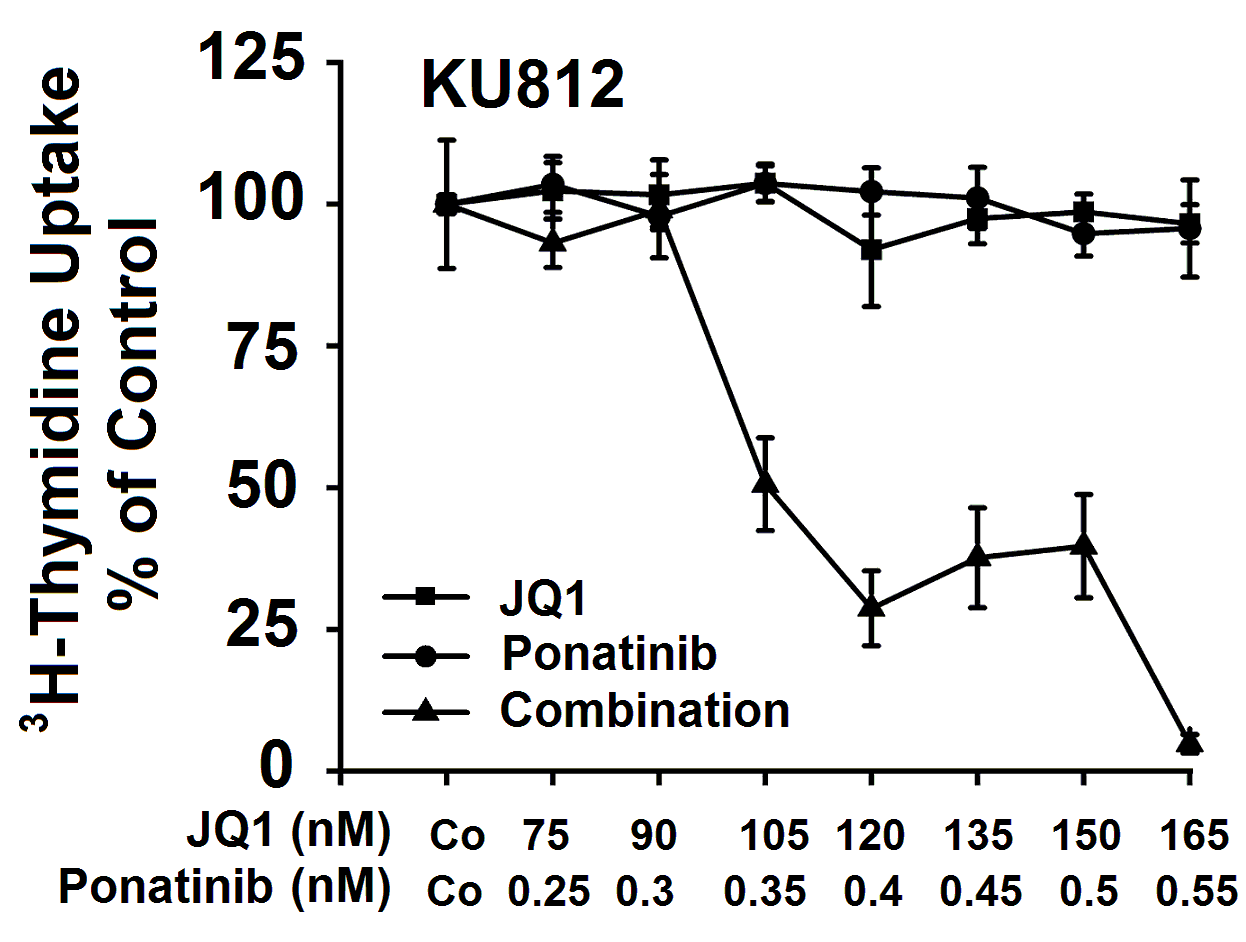


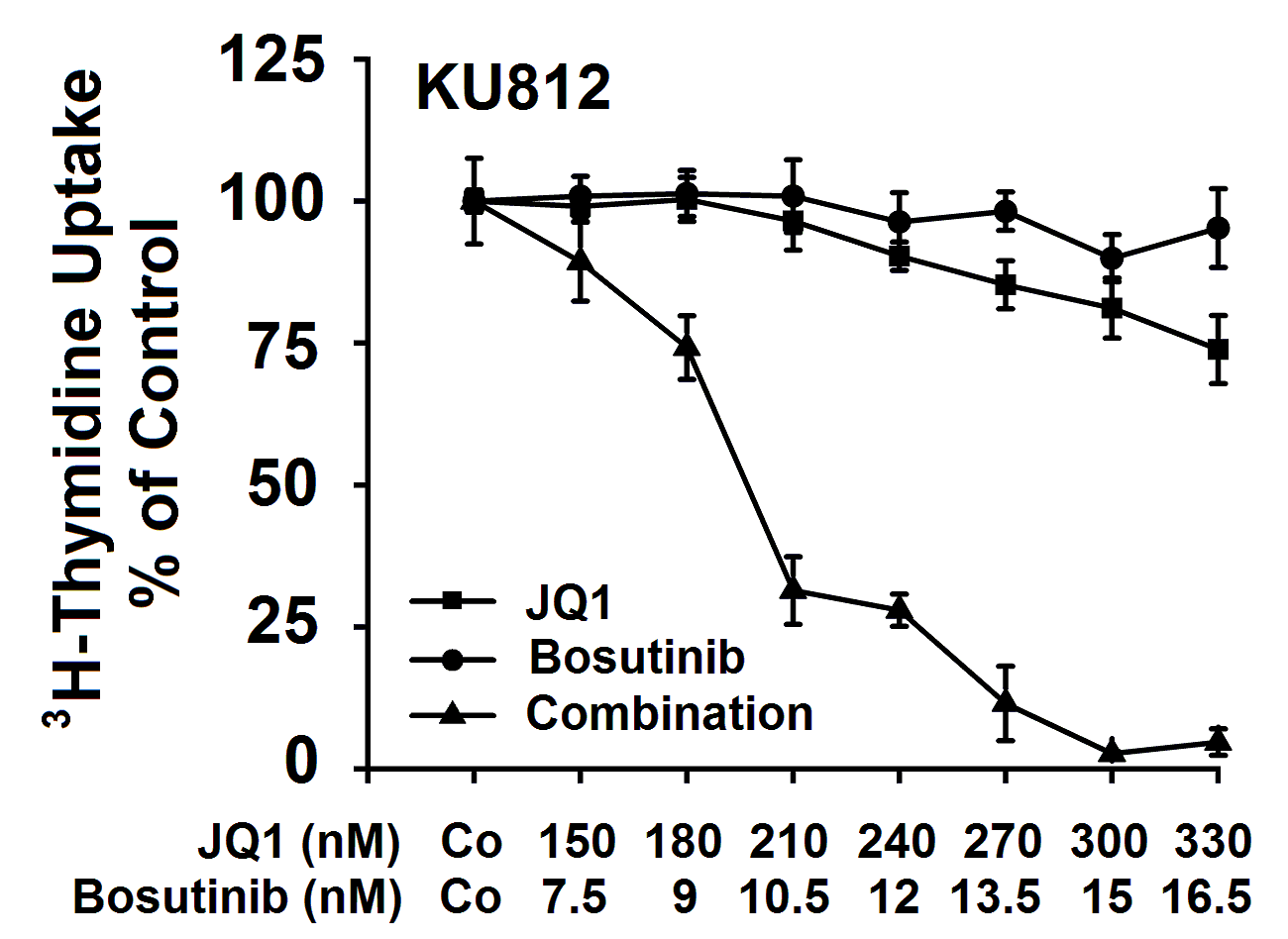


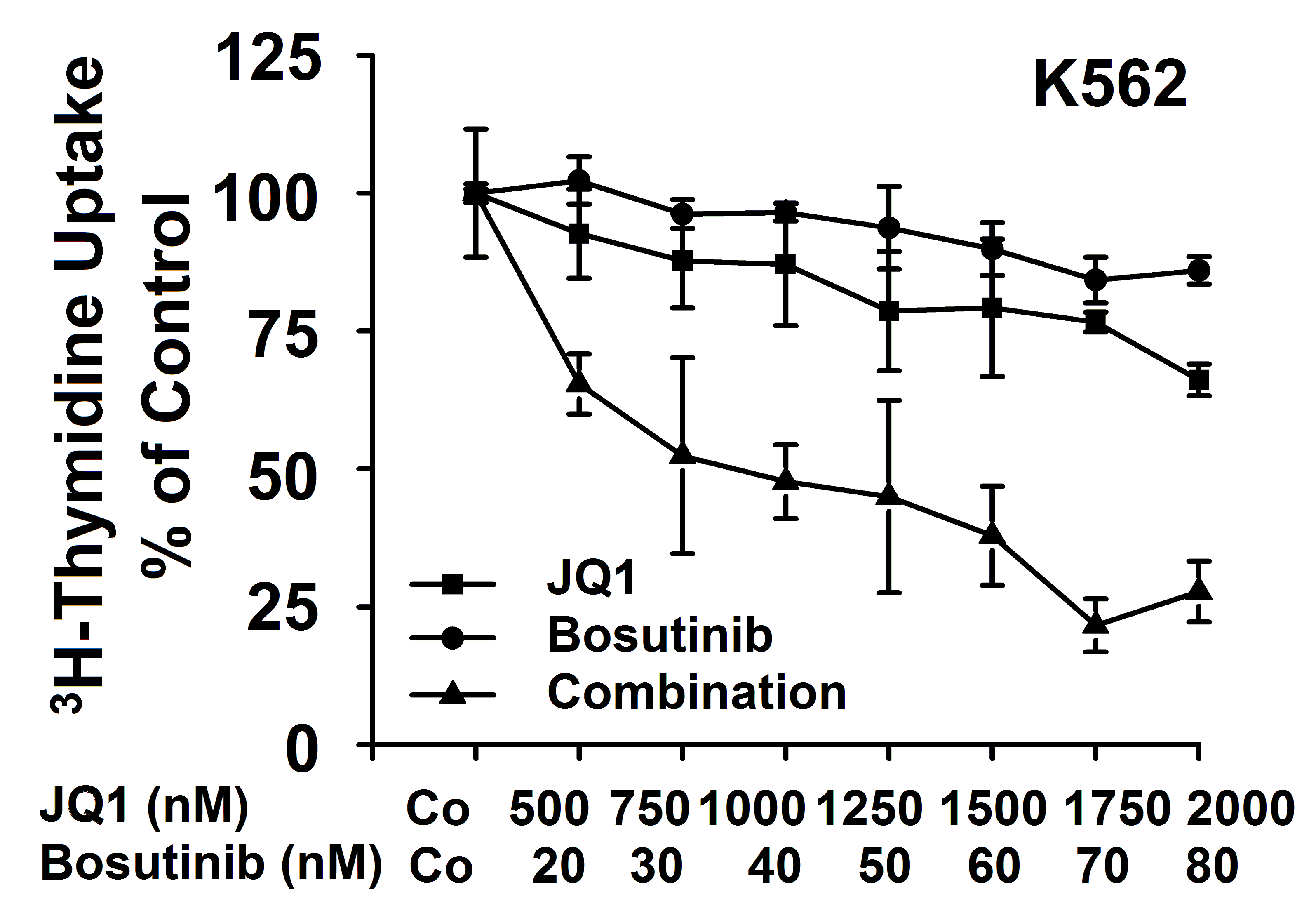


Peter et al., Supplemental Figure S8A


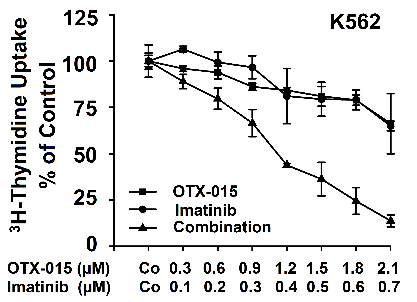

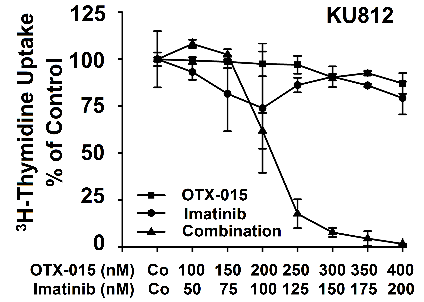


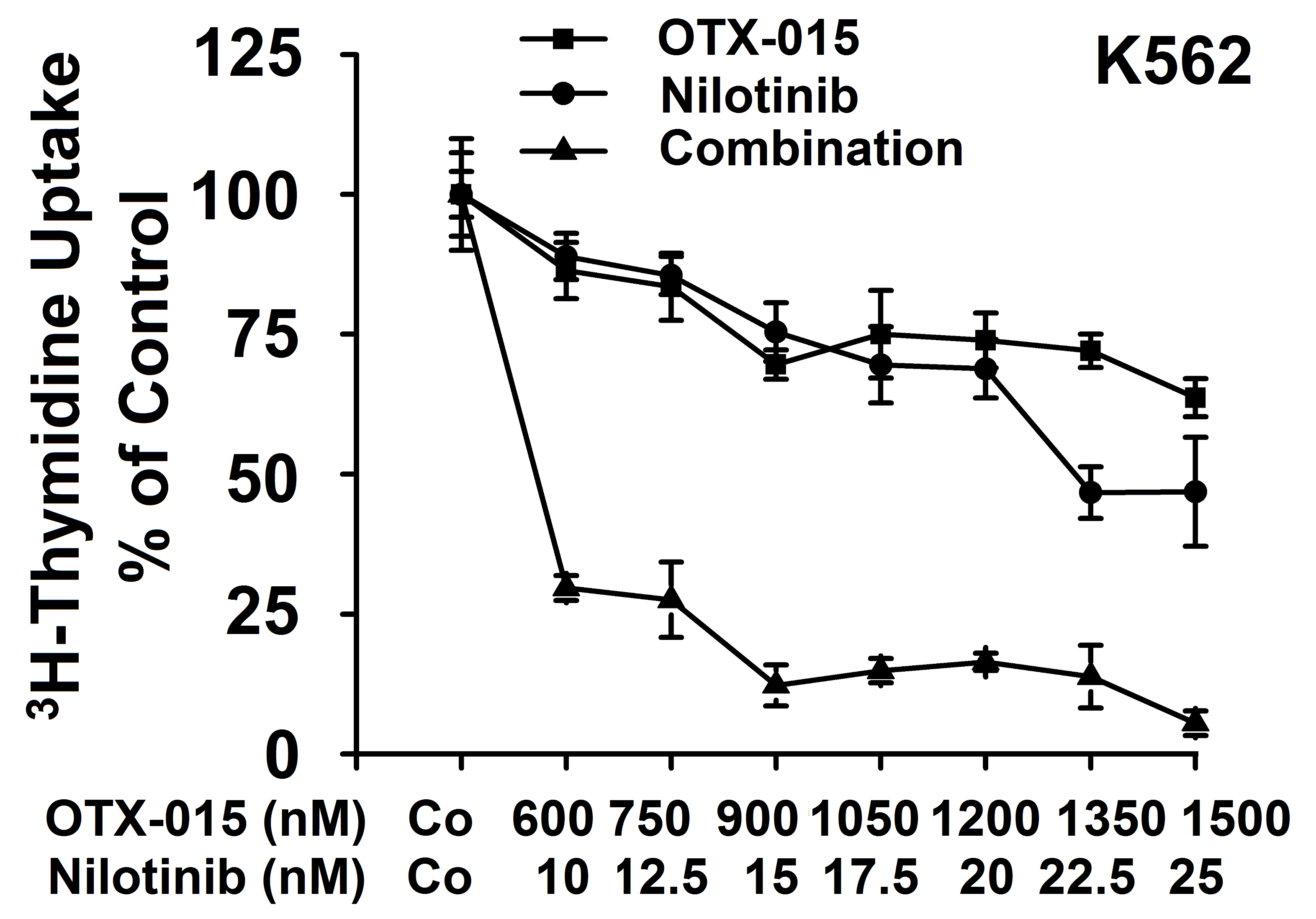

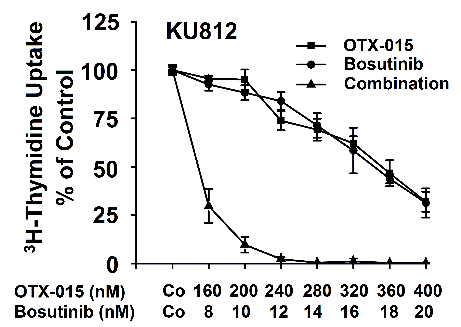


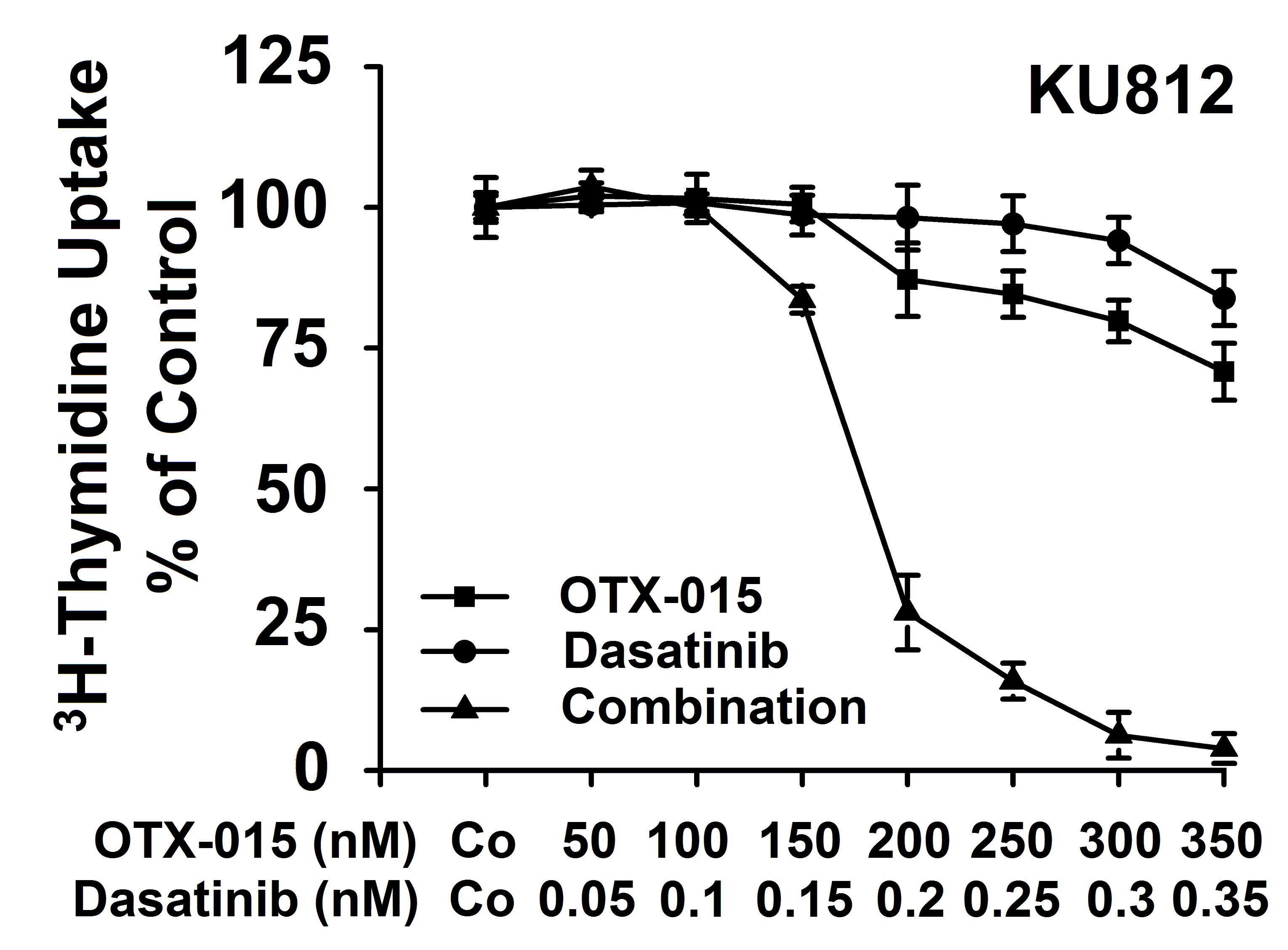

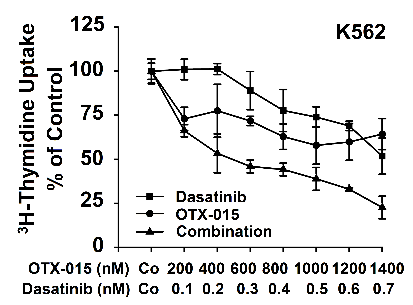


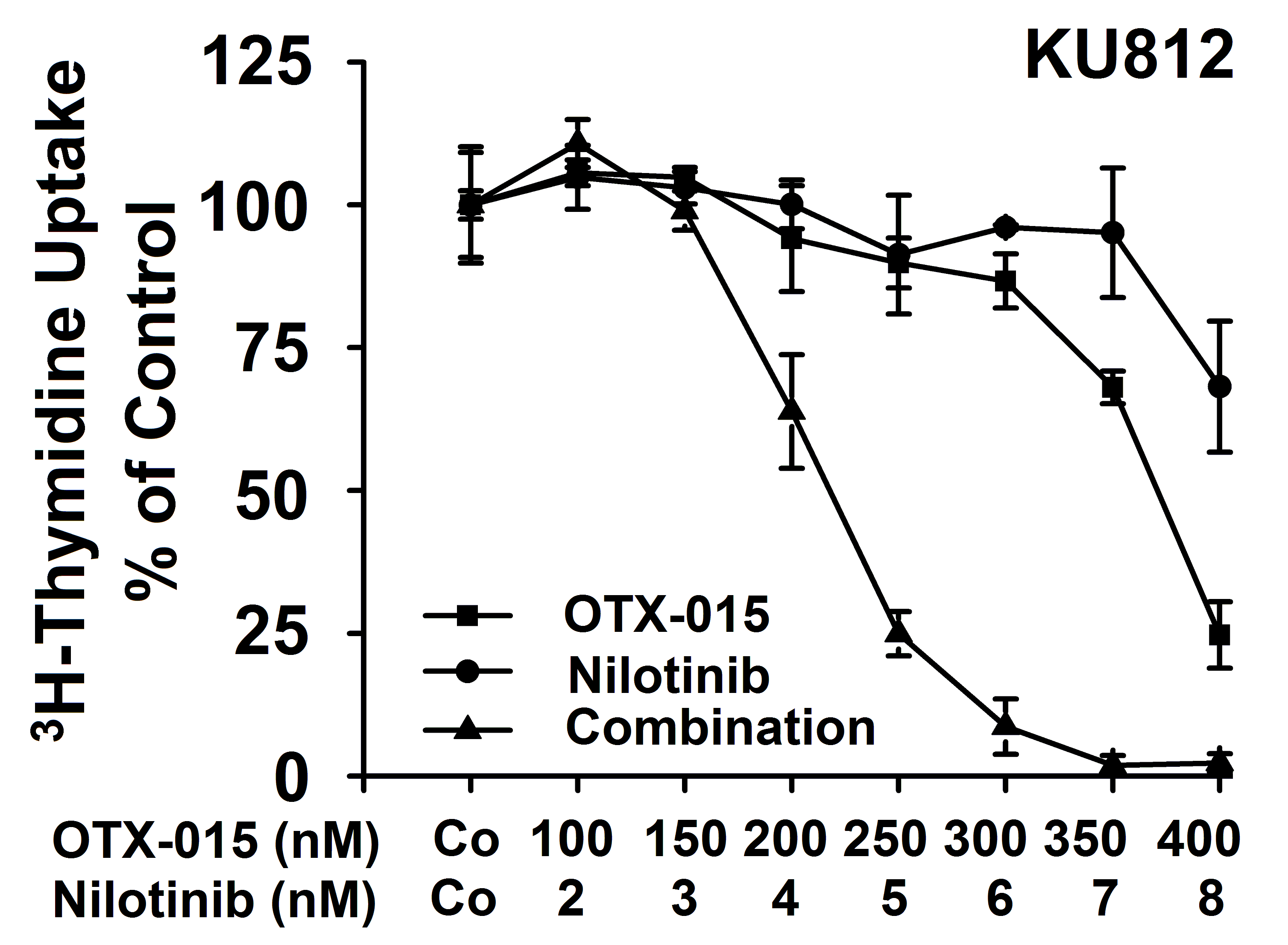


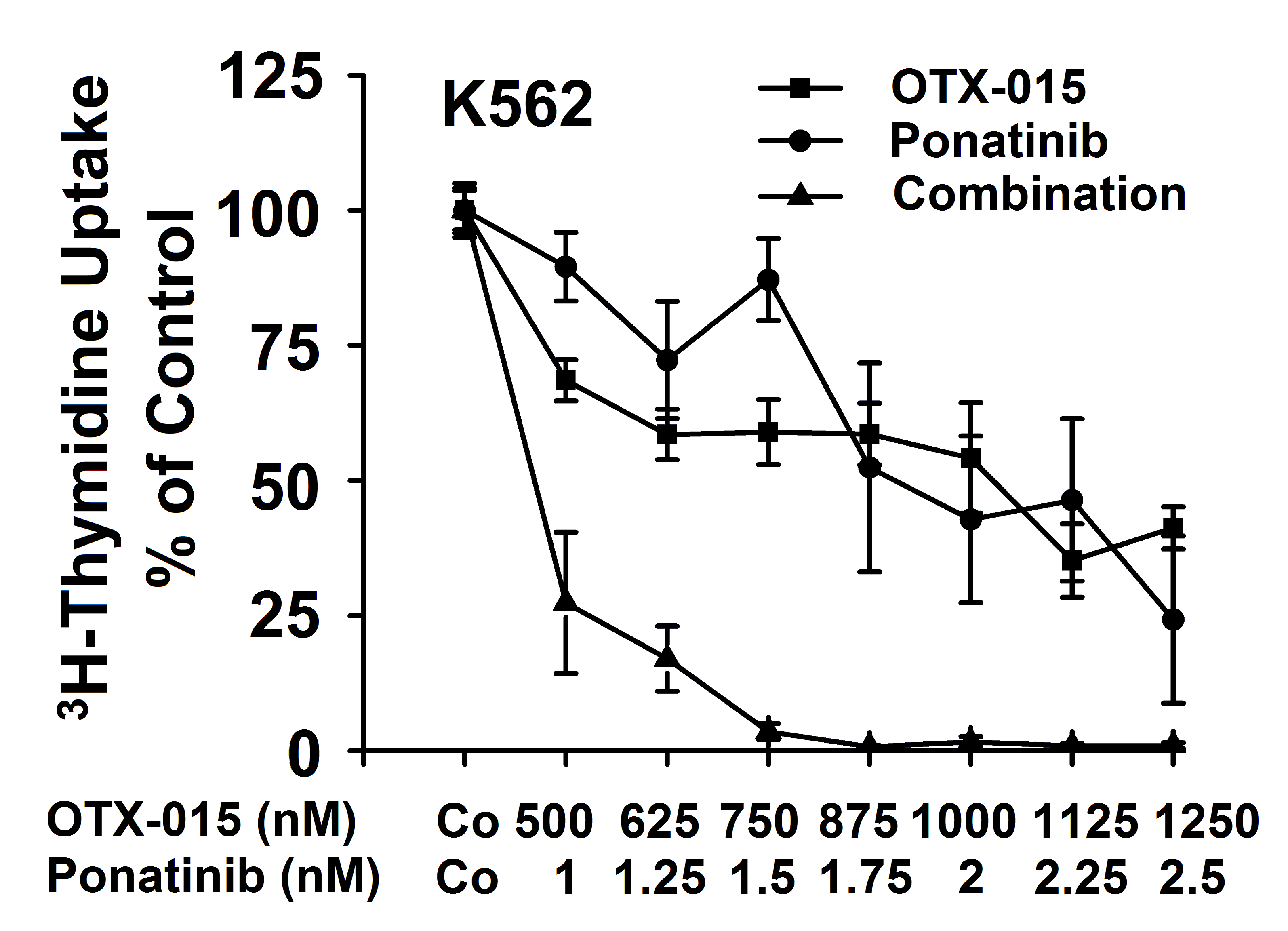

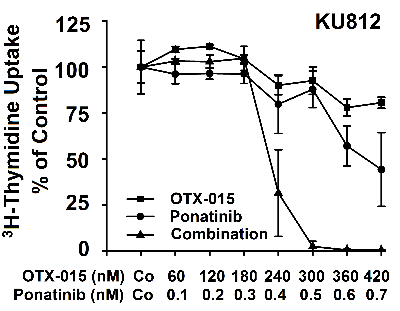


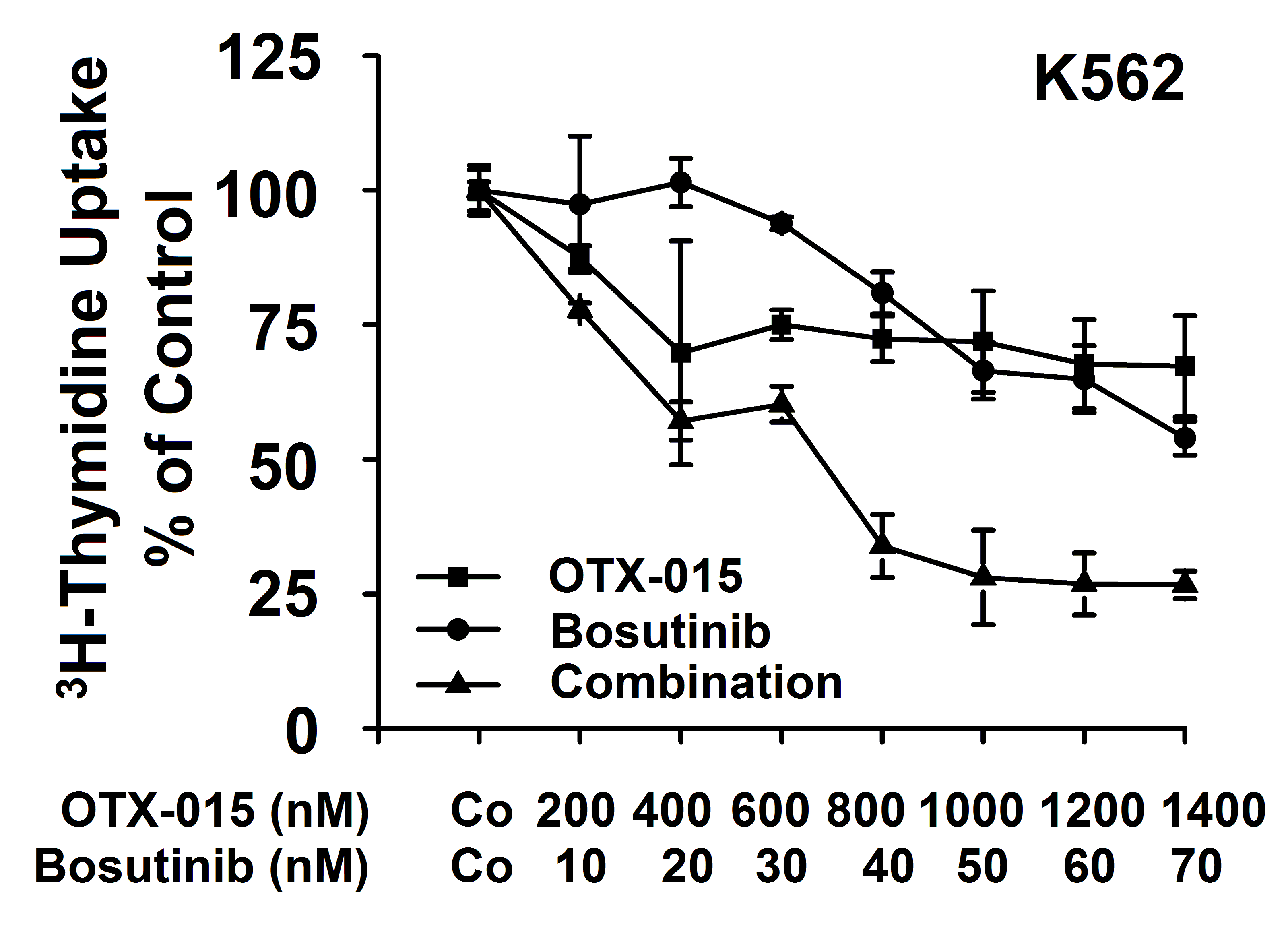


Peter et al., Supplemental Figure S8B


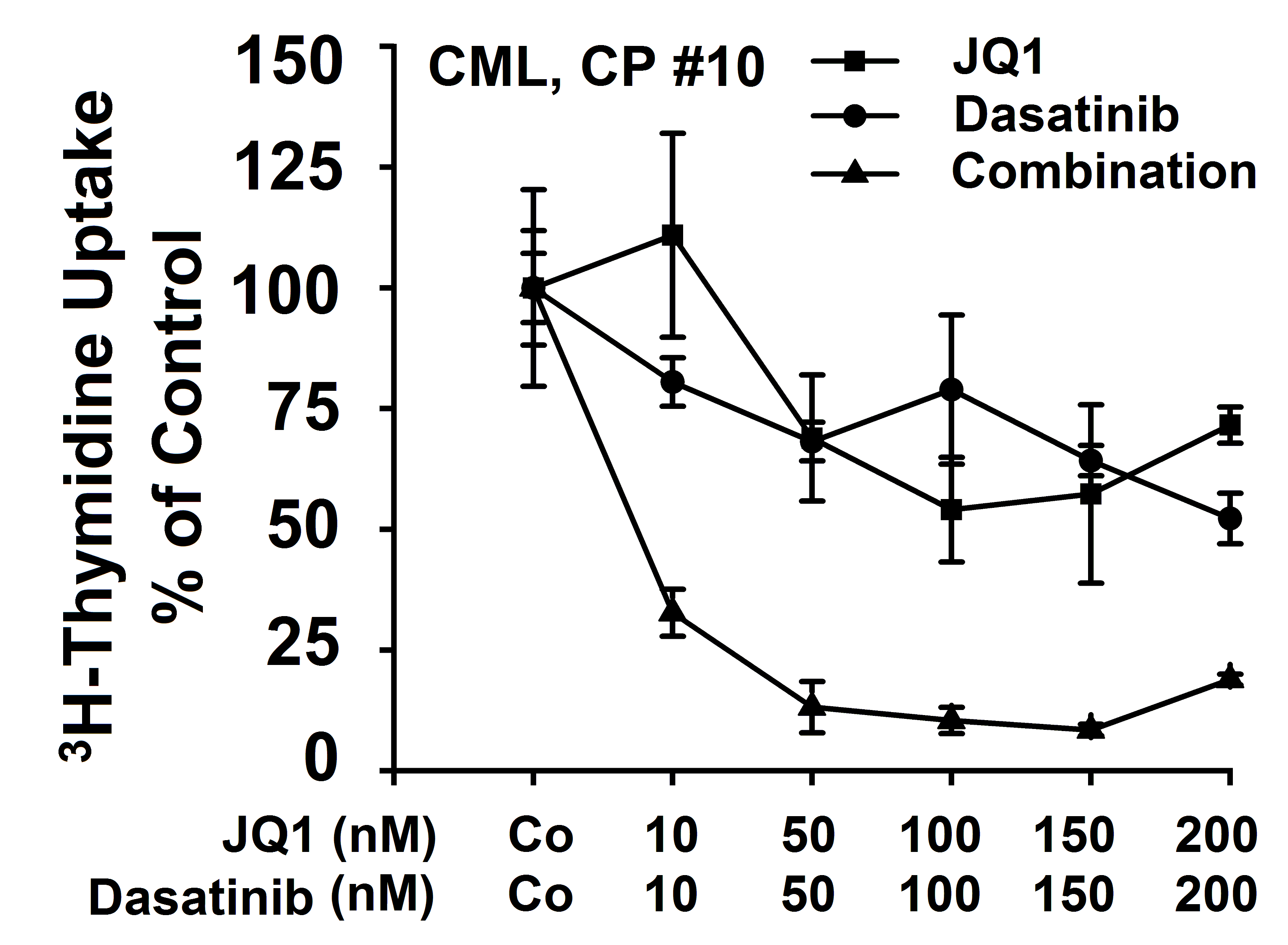

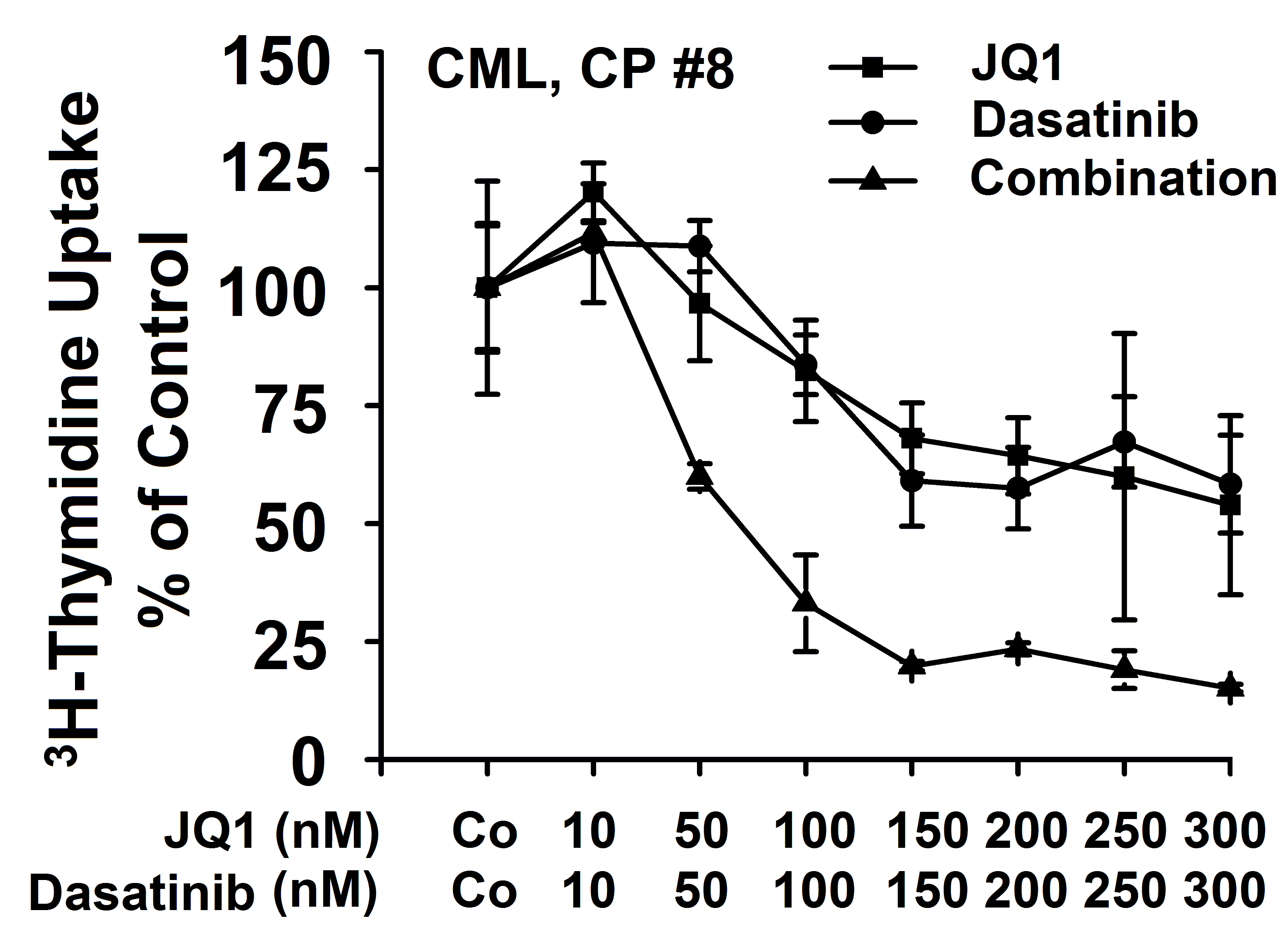


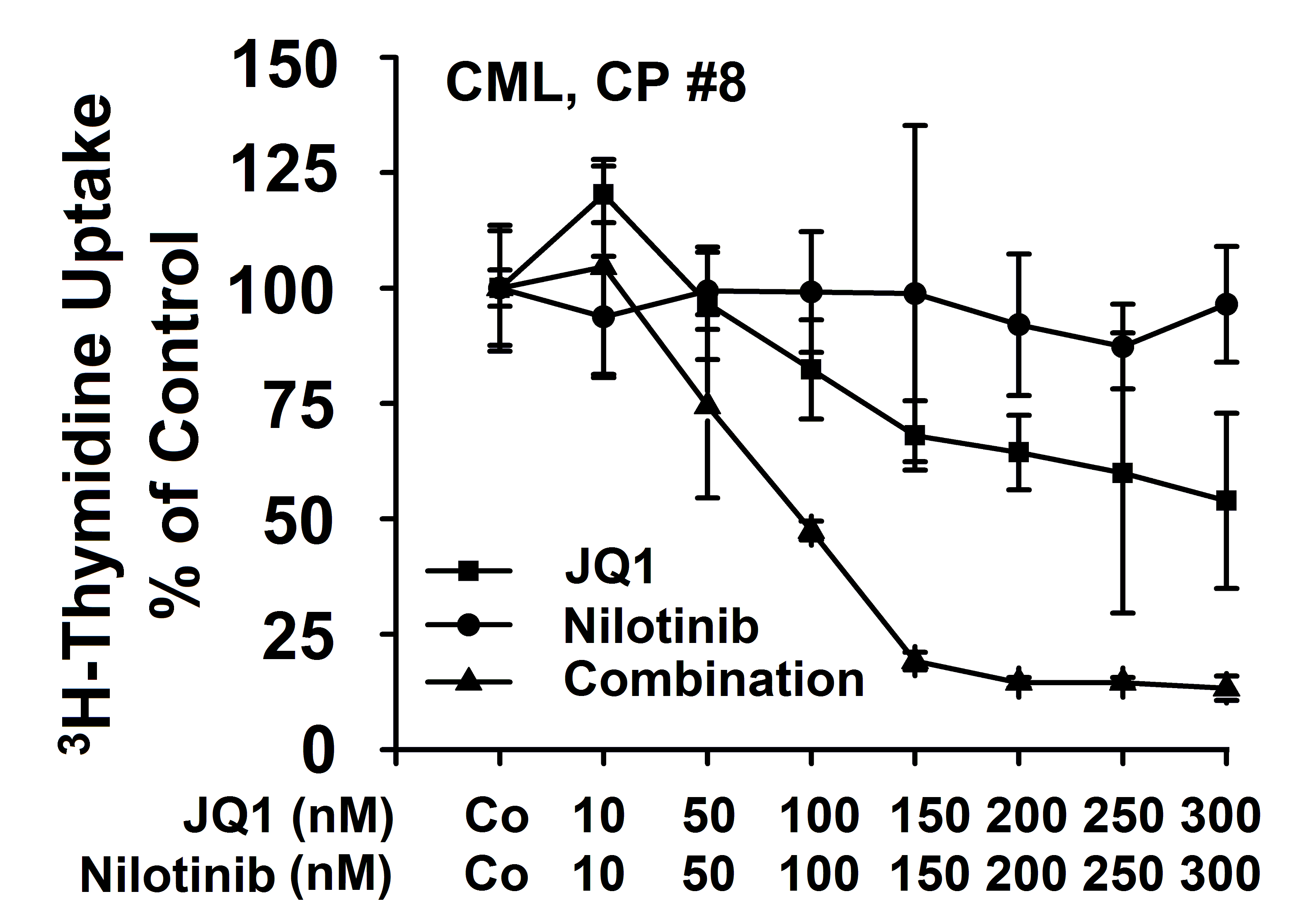

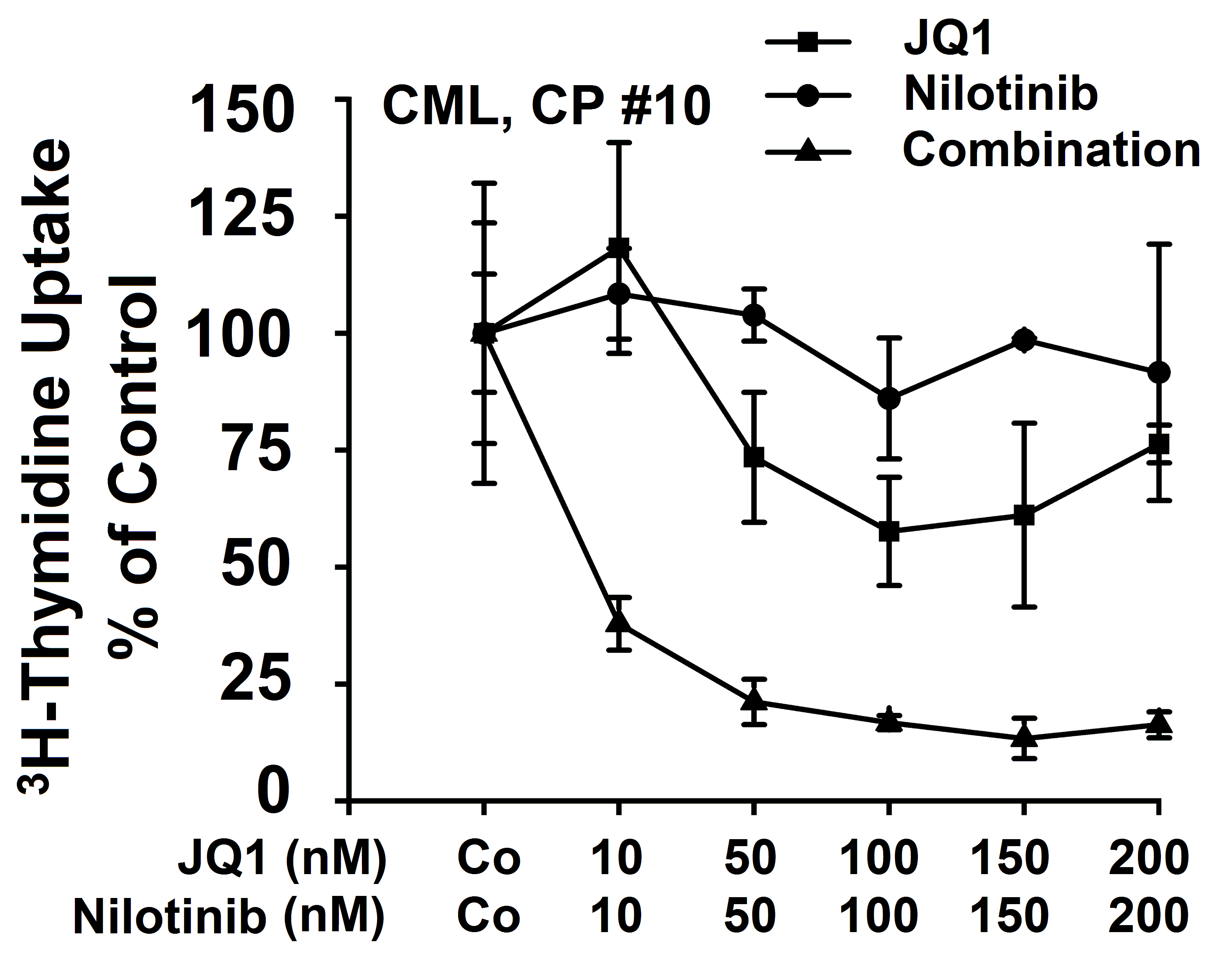


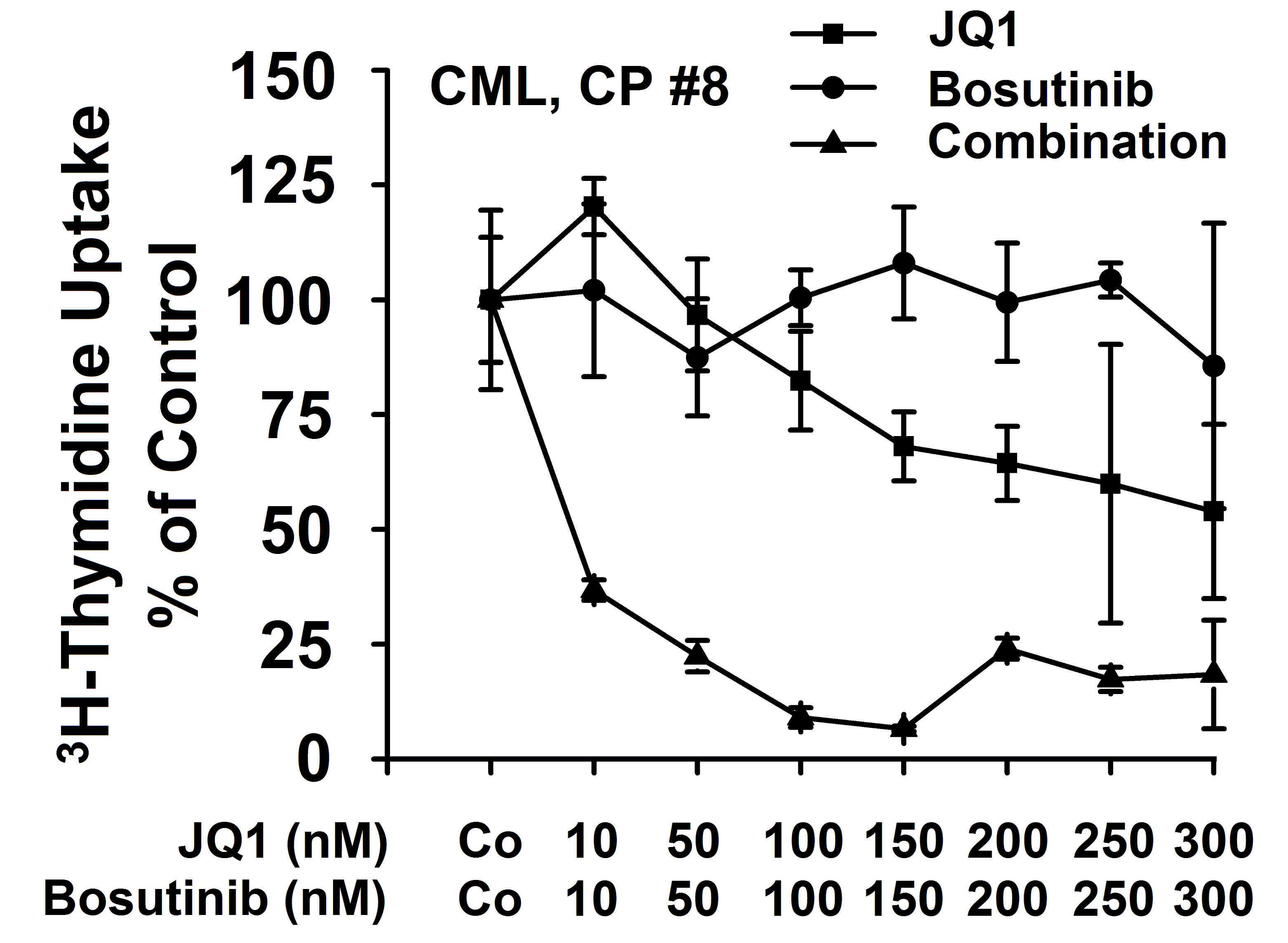

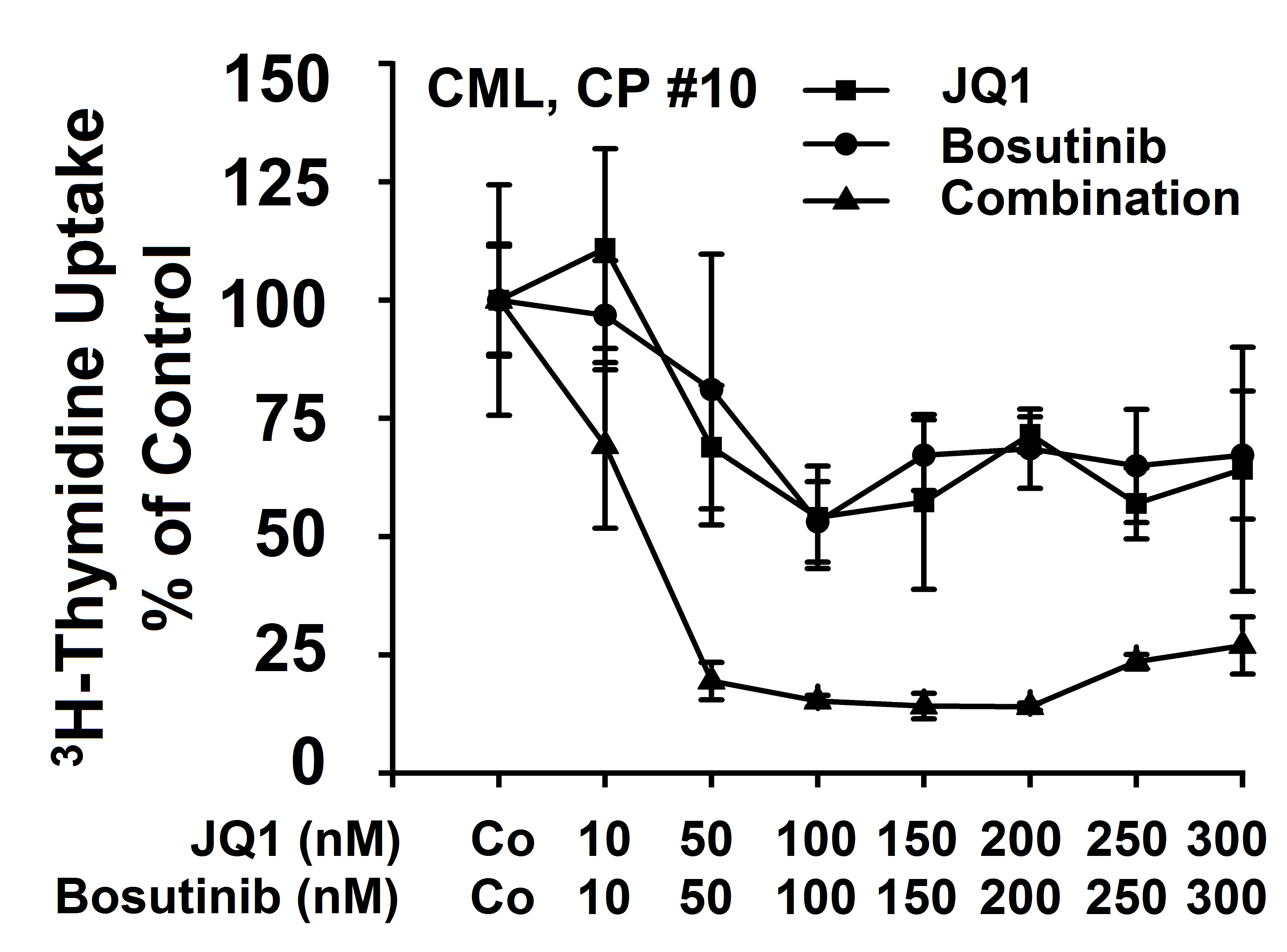


Peter et al., Supplemental Figure S8C


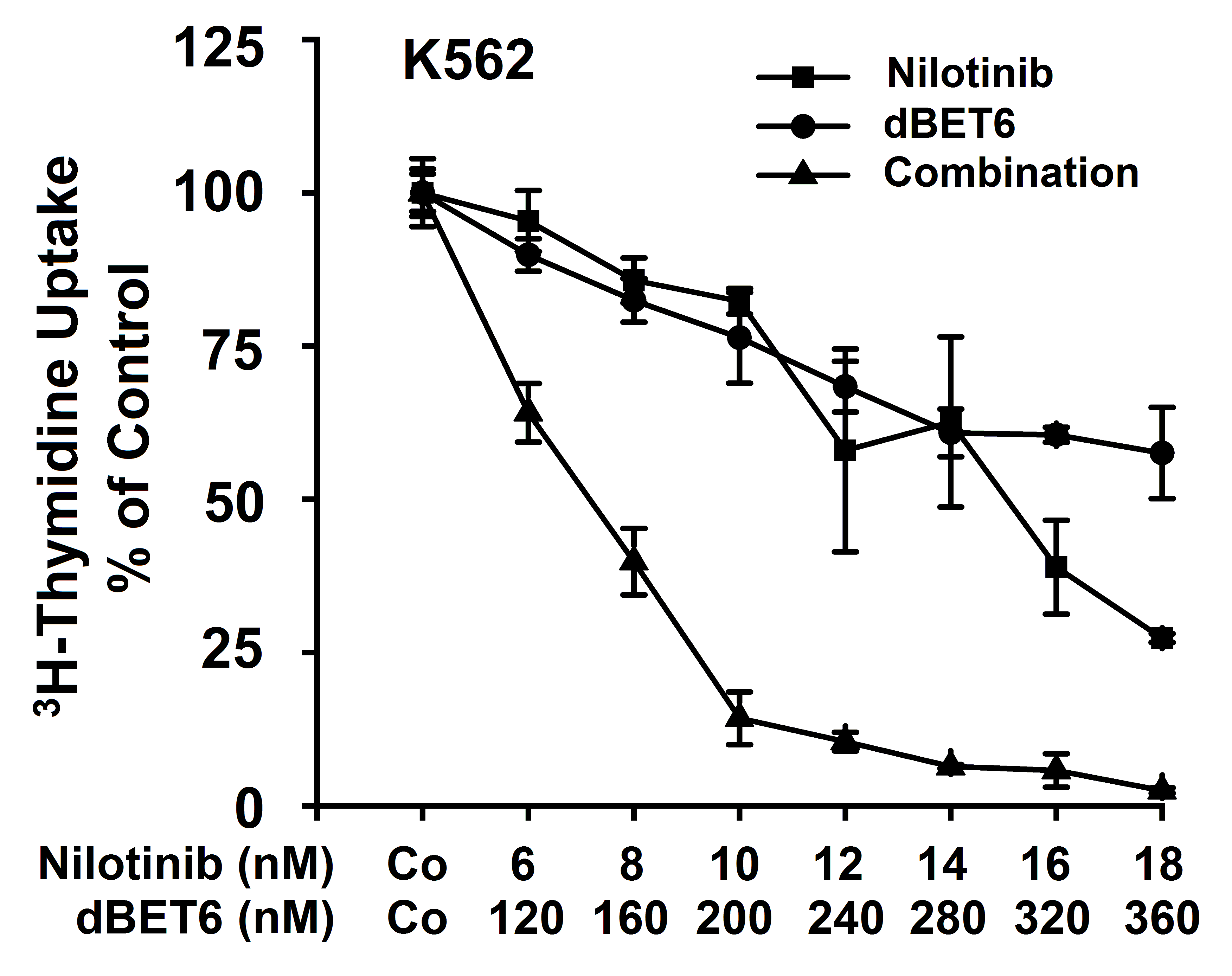

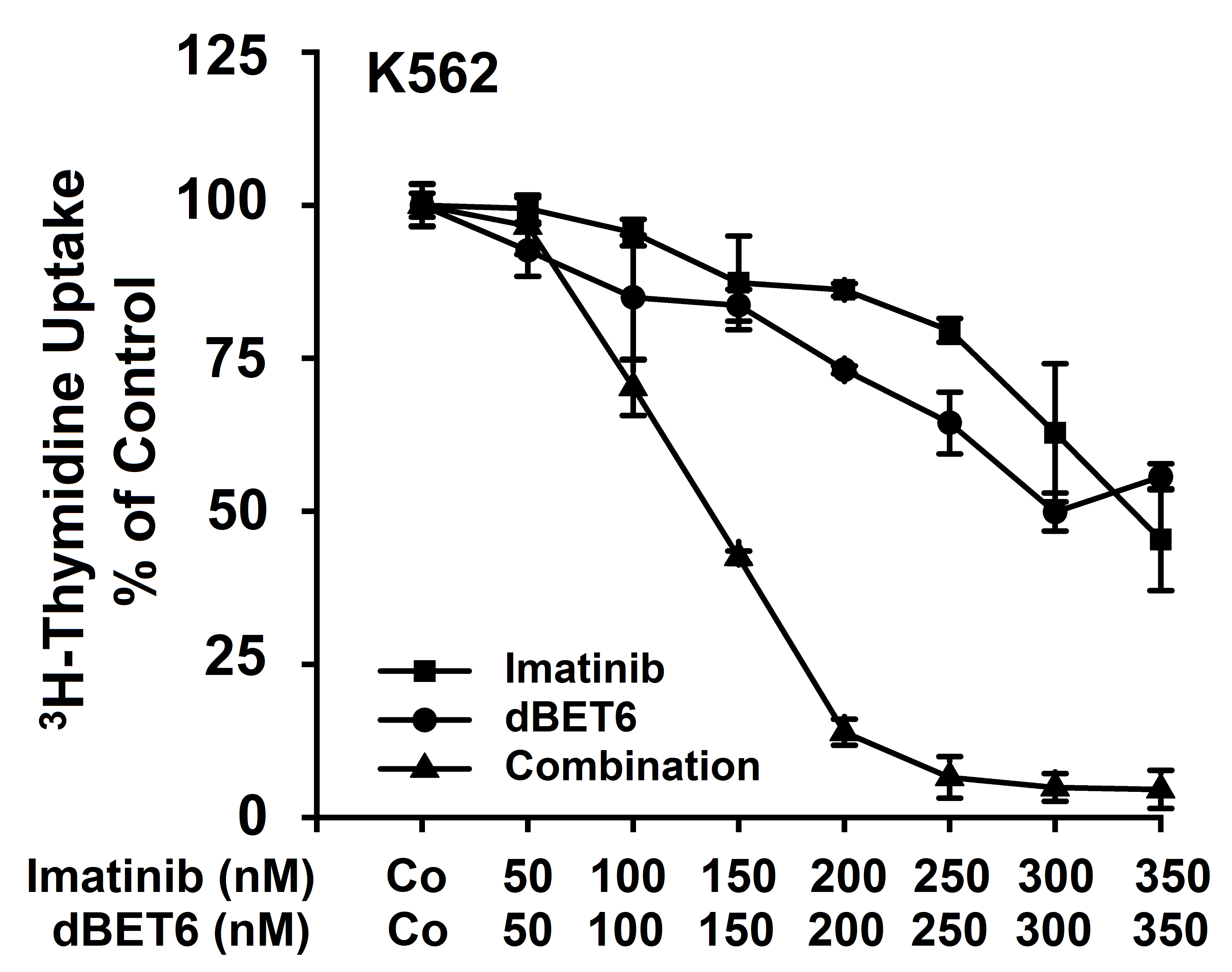

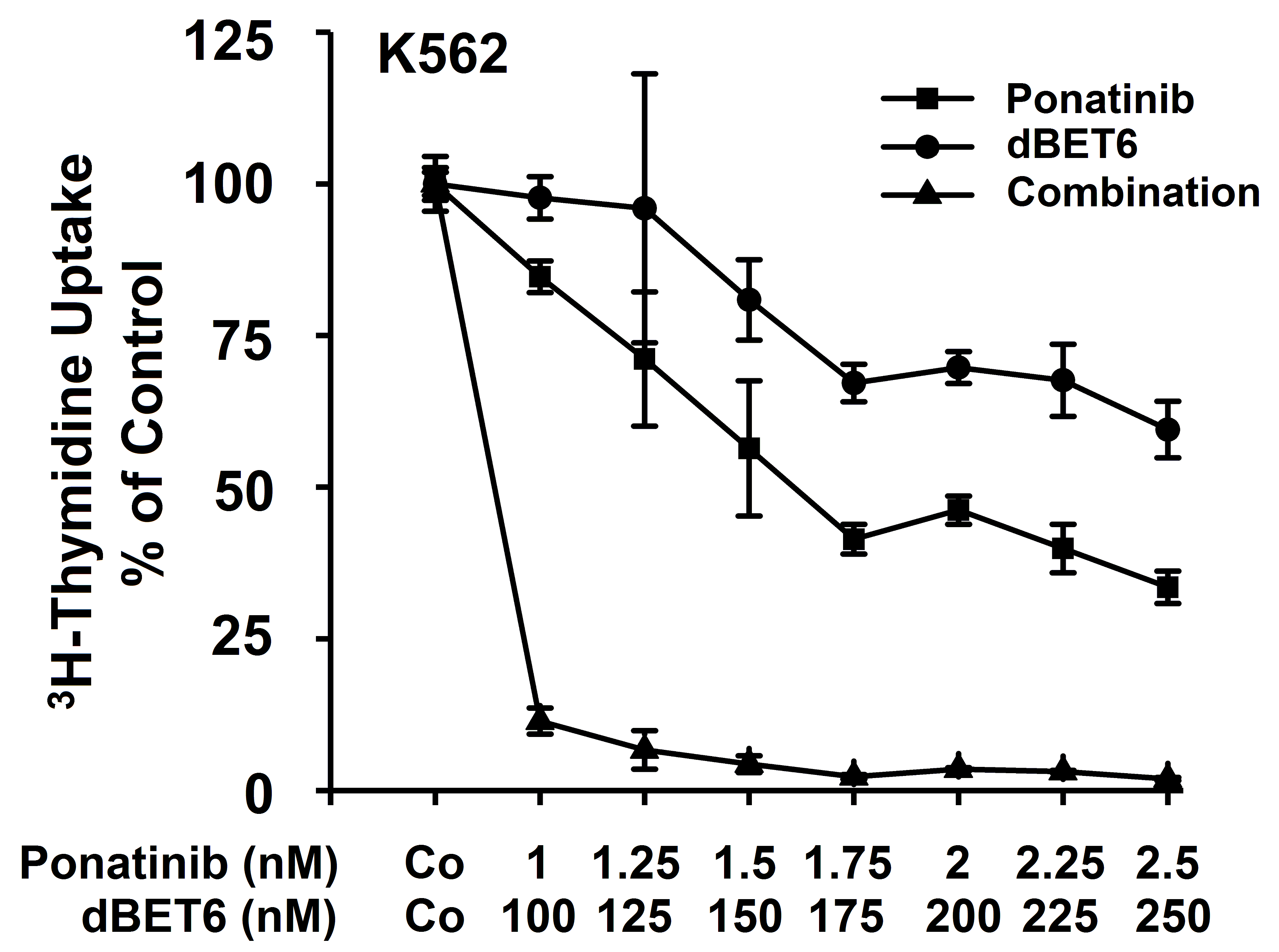

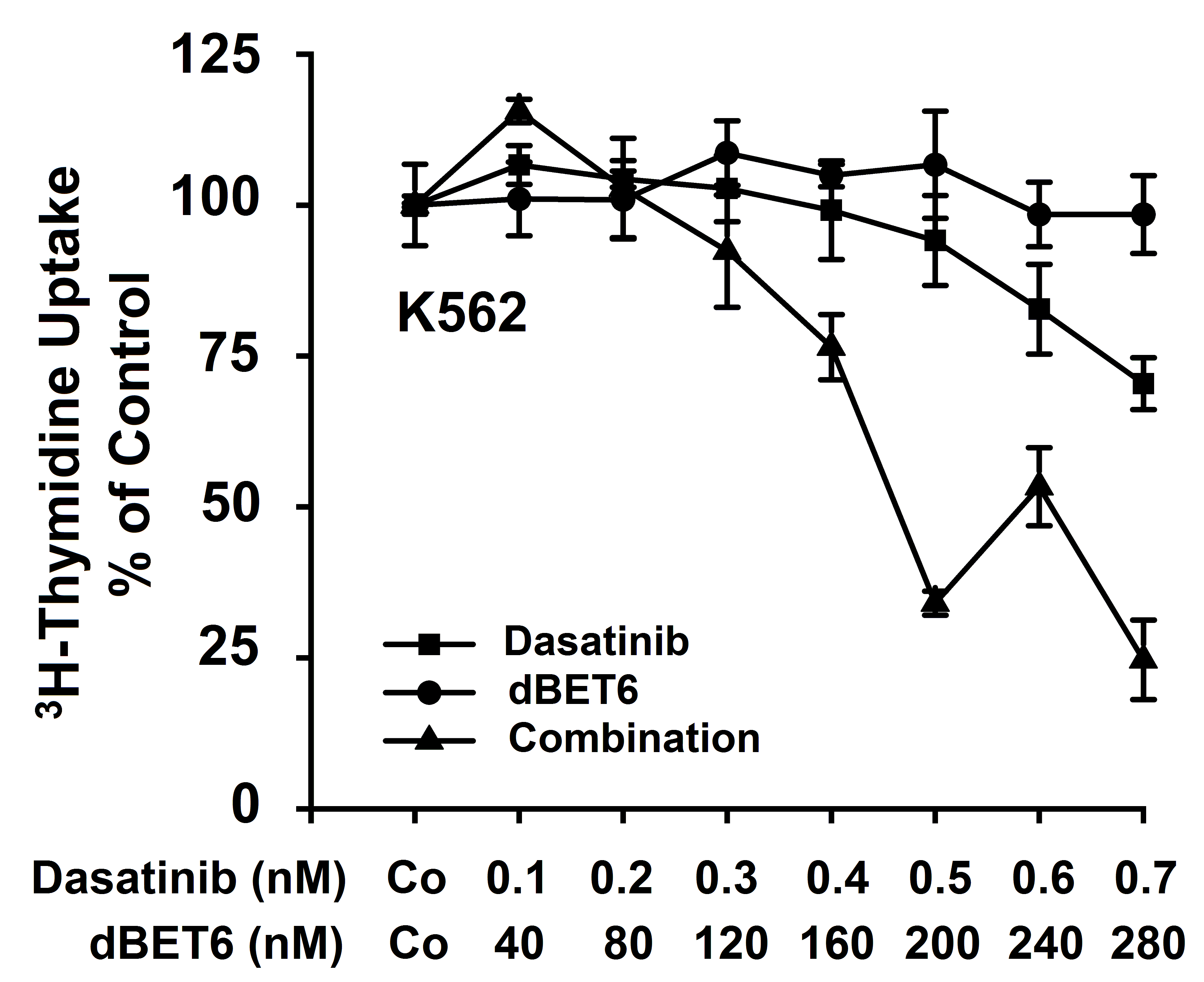

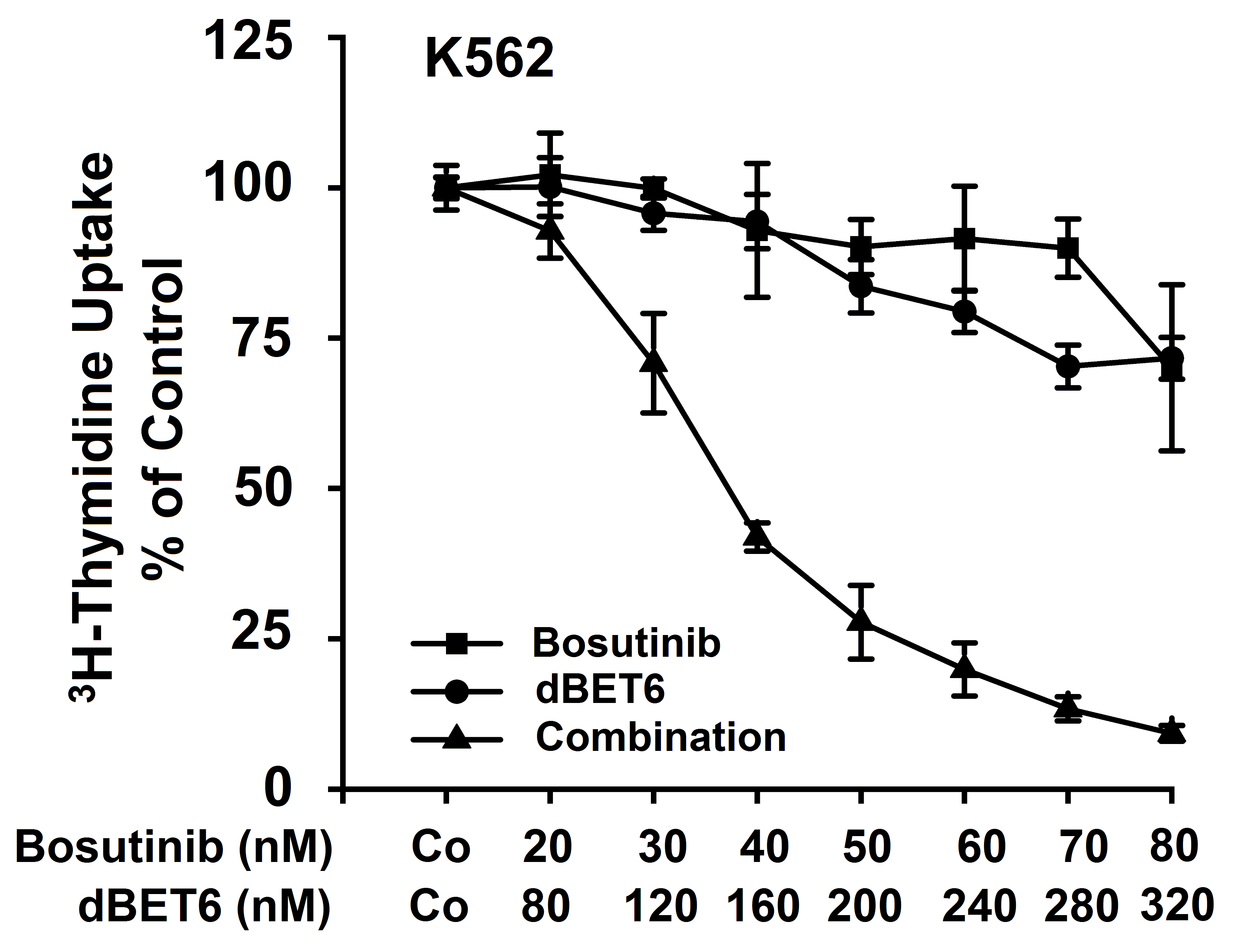


Peter et al., Supplemental Figure S8D


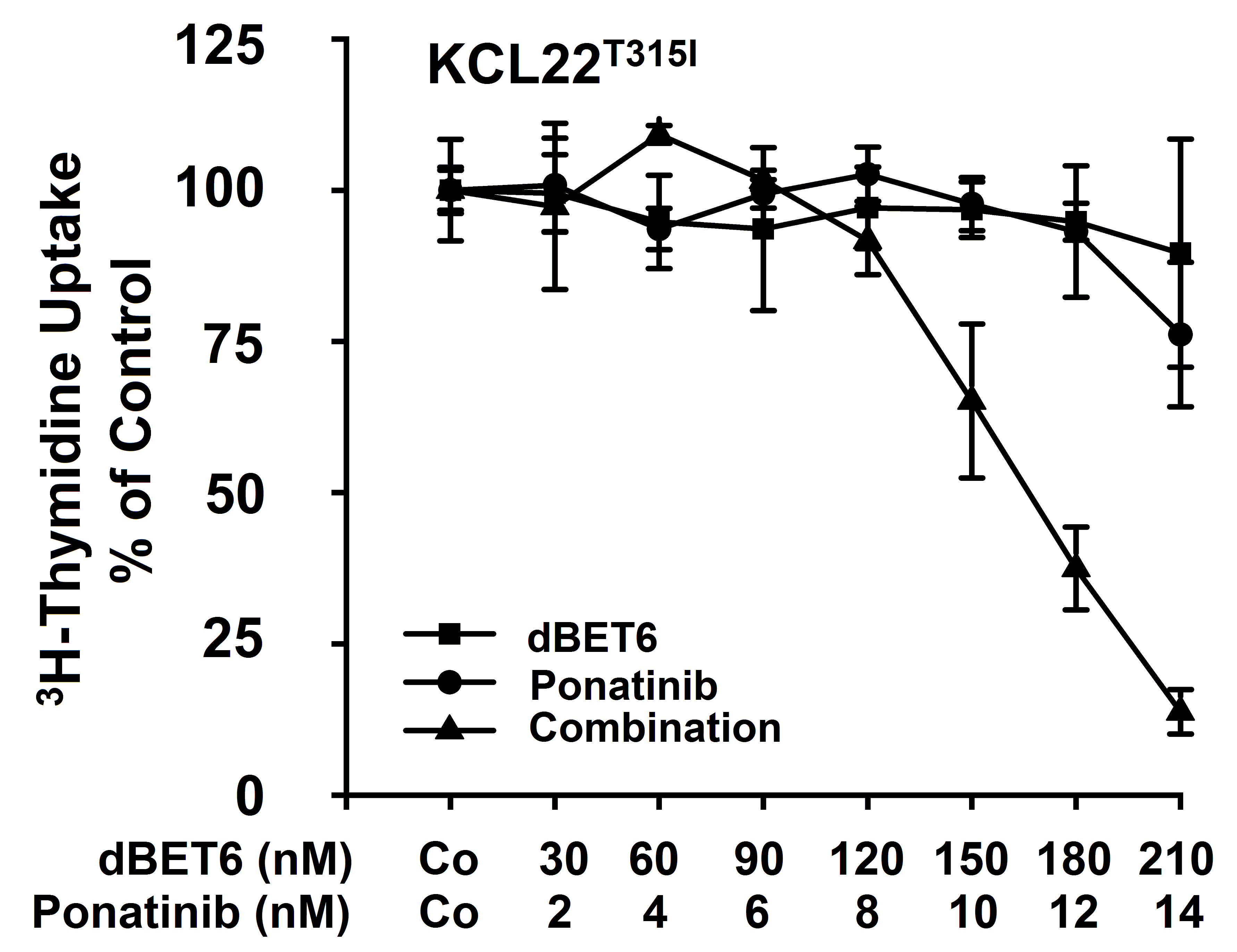


Peter et al., Supplemental Figure S8E

**Synergistic growth-inhibitory effects of BET-targeting drugs in combination with TKI**

A: KU812 cells (left panel) or K562 cells (right panel) were incubated with JQ1 alone, TKI alone or a combination of both drugs at a fixed ratio at 37°C for 48 hours. Then, ^3^H-thymidine was added and 16 hours later bound radioactivity was measured in a β-counter. Results are expressed as percent of control and represent mean±SD from triplicates. B: KU812 cells (left panel) or K562 cells (right panel) were incubated with OTX-O15 alone or TKI alone or a combination of both drugs at a fixed ratio at 37°C for 48 hours. Then, ^3^H-thymidine was added and 16 hours later bound radioactivity was measured in a β-counter. Results are expressed as percent of control and represent mean±SD from triplicates. C: Primary CML CP cells (MNC) were incubated with JQ1 alone, TKI alone or a combination of both drugs at a fixed ratio at 37°C for 48 hours. Results are expressed as percent of control and represent mean±SD from triplicates. D: K562 cells were incubated with dBET6 alone, TKI alone or a combination of both drugs at a fixed ratio at 37°C for 48 hours. Results are expressed as percent of control and represent mean±SD from triplicates. E: KCL22^T315I^ cells were incubated with dBET6 alone, ponatinib alone, or in a combination of both drugs at a fixed ratio at 37°C for 48 hours. Results are expressed as percent of control and represent the mean±SD from triplicates.

Supplemental Figure S9


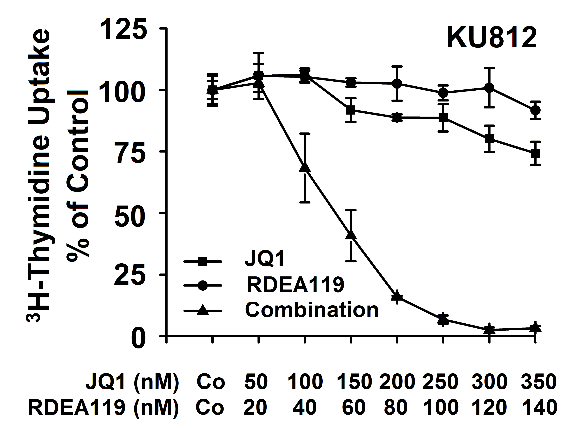

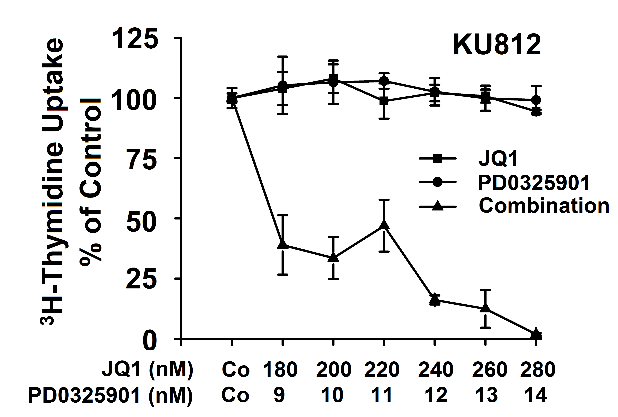


Peter et al., Supplemental Figure S9A


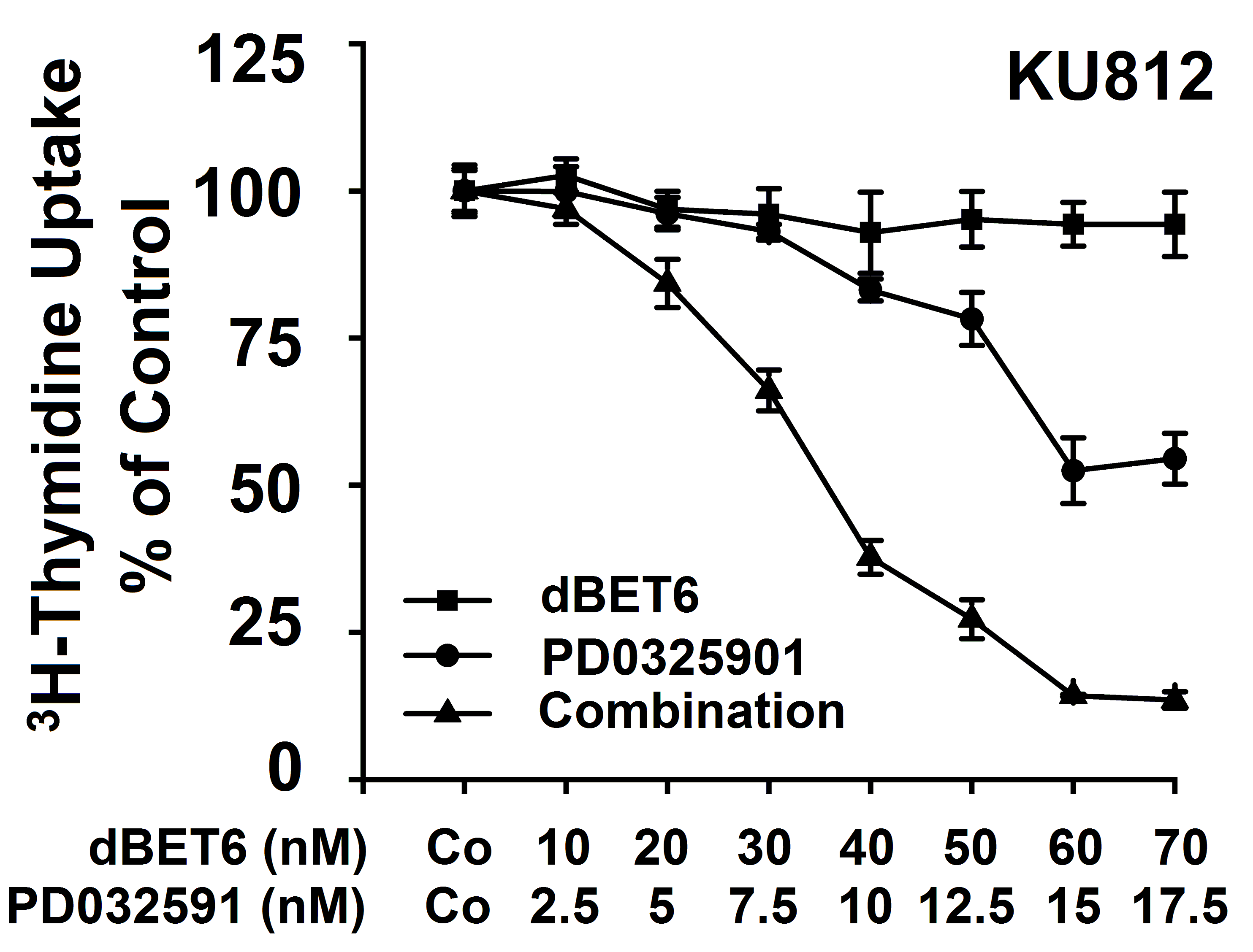

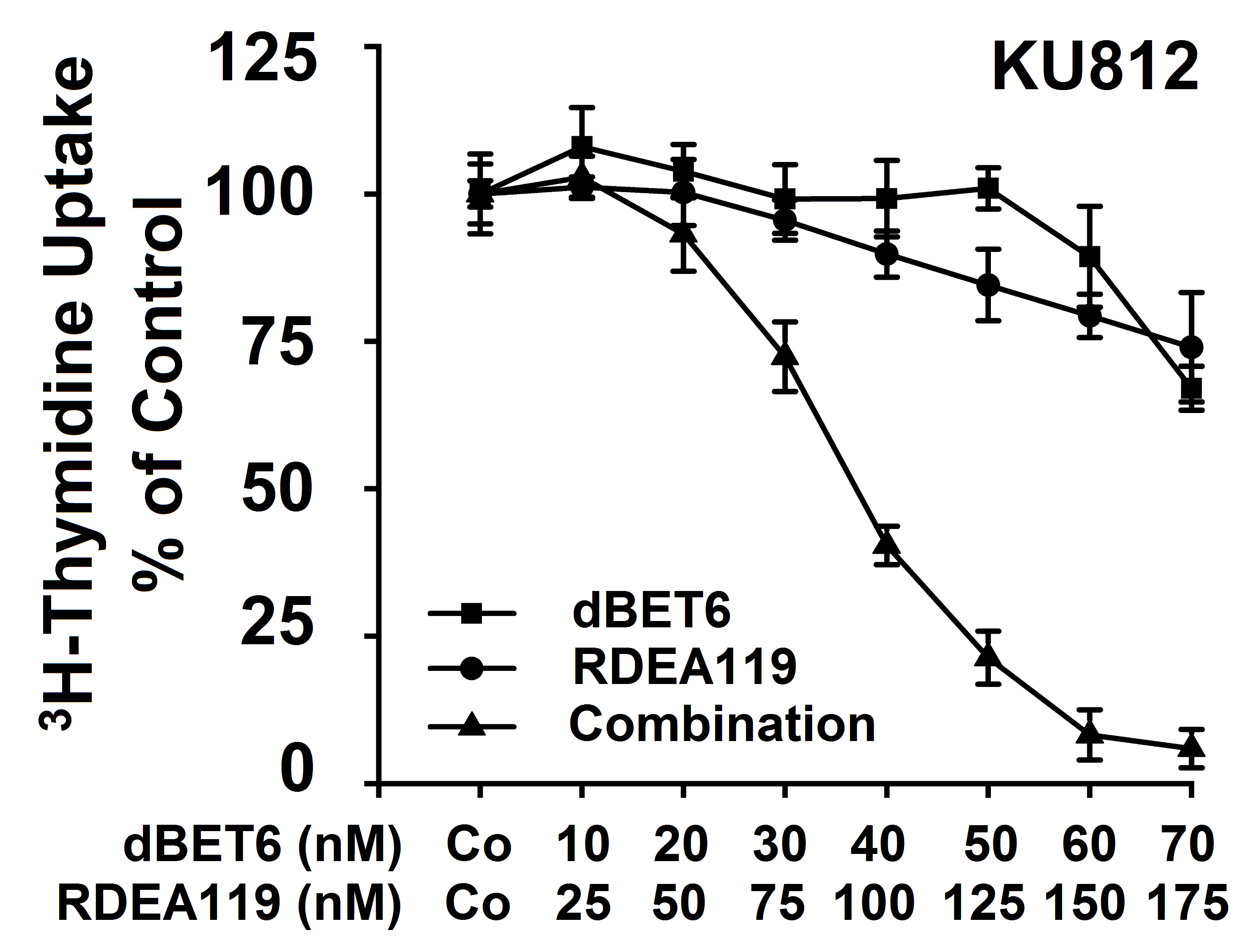


Peter et al., Supplemental Figure S9B

**Synergistic growth-inhibitory effects of JQ1 or dBET6 in combination with RDEA119 (refametinib) and PD0325901**

A: KU812 cells were incubated with the MEK inhibitors RDEA119 (refametinib) or PD0325901 alone or with JQ1 alone or a combination of both drugs at a fixed ratio at 37°C for 48 hours. Then, ^3^H-thymidine was added and 16 hours later bound radioactivity was measured in a β-counter. Results are expressed as percent of control and represent mean±SD from triplicates. B: KU812 cells were incubated with the MEK inhibitors RDEA119 (refametinib) or PD0325901 alone, dBET6 alone, or in a combination of both drugs at a fixed ratio at 37°C for 48 hours. Then, ^3^H-thymidine was added and 16 hours later bound radioactivity was measured in a β-counter. Results are expressed as percent of control and represent mean±SD from triplicates.

Supplemental Figure 10

Peter et al., Supplemental Figure S10A Peter et al., Supplemental Figure S10B

**Effects of dBET6 on viability and growth of CAL-72 cells**

A: CAL-72 cells were incubated in the presence (in co-culture) or absence (alone) of CML LSC with or without 100 nM dBET6 at 37°C for 48 hours. Thereafter, the percentages of Annexin V+ cells were measured among DAPI-negative cells by flow cytometry as described in the text. Results are expressed as Annexin V+ cells (%) and represent the mean±SD from 4 independent experiments. B: CAL-72 cells were incubated with medium (Co) or various concentrations of dBET6 as indicated at 37°C for 48 hours. Results are expressed as percent of control and represent the mean±SD from 3 independent experiments.

Supplemental Figure 11

**Expression of MYC, BRD4, HOXB4 and CCND2 in CML cell lines co-cultured with CAL-72**

KU812 cells (left panel) and K562 cells (right panel) were cultured in the absence or presence of CAL-72 cells at 37°C for 48 hours. Thereafter, cells were purified (separated from each other after co-culturing) by cell sorting as described in the text. qPCR was performed to analyze the expression of BRD4, MYC, HOXB4 and CCND2 mRNA in purified (sorted) KU812 cells and K562 cells obtained from the co-cultures. MYC, BRD4, HOXB4, and CCND2 mRNA levels are expressed as percent of GUSB mRNA levels and represent the mean±SD from 3 independent experiments.

Supplemental Figure S12

**Effects of IFN-G and BET degraders on CD47 and MDR-1 expression**

Primary CML CP MNC were incubated in medium or medium containing 200 U/ml IFN-G in the absence or presence of JQ1, dBET1 or dBET6 as indicated at 37°C for 24 hours. Thereafter CD34^+^/CD38^−^ LSC were analyzed for CD47 expression (left panel) and MDR-1 expression (right panel). Results are expressed as MFI (mean fluorescence intensity) percent of control (without IFN-G) and represent as mean±SD from 5 independent experiments. Abbreviations: MDR-1, multi-drug resistance gene-1; LSC, leukemic stem cells; IFN-G, interferon-gamma.
